# Transcriptome Analysis of *Cinnamomum chago*: A Revelation of Candidate Genes for Abiotic Stress Response and Terpenoid and Fatty Acid Biosyntheses

**DOI:** 10.3389/fgene.2018.00505

**Published:** 2018-11-05

**Authors:** Xue Zhang, Yue Zhang, Yue-Hua Wang, Shi-Kang Shen

**Affiliations:** School of Life Sciences, Yunnan University, Kunming, China

**Keywords:** Lauraceae, transcriptome, adaptation, molecular makers, terpenoid, abiotic stress

## Abstract

*Cinnamomum chago*, an endangered species endemic to Yunnan province, possesses large economic and phylogenetic values in Lauraceae. However, the genomic information of this species remains relatively unexplored. In this study, we used RNAseq technology to characterize and annotate the *C. chago* transcriptome and identify candidate genes involved in special metabolic pathways and gene-associated simple sequence repeats (SSRs) and single-nucleotide polymorphism (SNP). A total of 129,097 unigenes, with a mean length of 667 bp and an N50 length of 1,062 bp, were assembled. Among these genes, 56,887 (44.07%) unigenes were successfully annotated using at least one database. Furthermore, 47 and 46 candidate genes were identified in terpenoid biosynthesis and fatty acid biosynthesis, respectively. A total of 22 candidate genes participated in at least one abiotic stress response of *C. chago*. Additionally, a total of 25,654 SSRs and 640 SNPs were also identified. Based on these potential loci, 55 novel expressed sequence tag (EST)-SSR primers were successfully developed. This work provides comprehensive transcriptomic data that can be used to establish a valuable information platform for gene prediction, signaling pathway investigation, and molecular marker development for *C. chago* and other related species. Such a platform can facilitate further studies on germplasm conservation and utilization of Lauraceae species.

## Introduction

Genomic/transcriptomic techniques are information tools used to assess the physiological conditions of organisms in response to multiple stressors (Allendorf et al., 2010). Next-generation sequencing (NGS) is an inexpensive technology that can produce larger volumes of sequencing data than conventional methods, and NGS has revolutionized genomic and transcriptomic approaches in the field of biology (Metzker, 2010; Davey et al., 2011). With the advent of NGS and *de novo* assembly technology, RNA sequencing (RNA-seq) has been proven to be an efficient and cost-effective approach for studying the transcriptome of non-model organisms without using established genomic databases (Wang et al., 2009; Berdan et al., 2016). Also, RNA-seq can be used for the simultaneous study of nucleotide variations, gene expression levels across different tissue types, disparate time periods, and ecologically relevant variables in high resolution and large dynamic ranges (Wang et al., 2009; Harris et al., 2015). Therefore, this approach accelerates the examination of expression differences in ecologically important traits (Bonneaud et al., 2011; Schvartzman et al., 2018), phenotypic and behavioral plasticity (Aubin-Horth and Renn, 2009; Brawand et al., 2014), and genes with potential adaptive significance in changing environments for non-model species (Niu et al., 2015; Harris et al., 2015; Todd et al., 2016; Palma-Silva et al., 2016). This new sequencing tool is also valuable for the investigation, validation, and assessment of allelic information [e.g., single nucleotide polymorphisms (SNPs) and simple sequence repeats (SSRs)]. Furthermore, RNA-seq can be used to develop genetic markers for the population-level consequences of genetic variation (Davey et al., 2011; Zhang et al., 2017).

Species of Lauraceae are recognized for their significant ecological and economic value. Most of these species are not only conspicuous elements of tropical and subtropical evergreen broad-leaved forests but also important sources of camphor, spices, perfumes, nutritious fruits, phytomedicine, and high-quality wood (Ravindran et al., 2003; Wang et al., 2007; Huang et al., 2016). To date, the genome of Lauraceae species has not been completely sequenced. Scholars have obtained the transcriptome sequences of few economic species through RNA-seq. These species include *Lindera glauca* (Niu et al., 2015), *Litsea cubeba* (Han et al., 2013), *Neolitsea sericea* (Chen et al., 2015), and *Persea americana* (Chanderbali et al., 2009). Thus, the conversion and utilization of Lauraceae are complicated and restricted because of insufficiently systematic and in-depth research on the genomic/transcriptomic information of specific compounds and on the adaptability of these plants to a heterogeneous environment.

To date, transcriptome studies on Lauraceae have focused on the biosynthesis of terpenoids (Niogret et al., 2013; Niu et al., 2015; Yan et al., 2017) and fatty acids (FAs; Kilaru et al., 2015; Ibarra-Laclette et al., 2015). Few works have investigated the potential genes associated with the responses of laurel plants to multiple stresses under changing environments. Similar to other living organisms, plants are subjected simultaneously to different abiotic stresses, such as high irradiance, extreme temperature, high salinity, and water deprivation (Rizhsky et al., 2004). These stresses can disturb cellular homeostasis and lead to severe retardation in growth and development and death (Kotak et al., 2007). Therefore, the complex regulatory networks and metabolic pathways of plants in response to single and multiple concurrent abiotic stresses must be delineated to promote plant growth and development (Rasmussen et al., 2013). Elucidation of the transcriptome-level responses of plants to abiotic stresses offers a considerable opportunity to assess genes involved in the process of adaptation to environmental changes (Alvarez et al., 2015). Some studies described transcriptome changes in response to abiotic stresses in plants, such as *Arabidopsis thaliana* (Coolen et al., 2016), *Cucumis sativus* (Wang et al., 2014), *Tamarix hispida* (Yang et al., 2014), and *Solanum dulcamara* (Lortzing et al., 2017). These results revealed related genes or metabolism pathways that mediate stress tolerance.

*Cinnamomum chago*, an endangered Lauraceae species, was first reported by Sun and Zhao (1991). *C. chago* possesses evident morphological features similar to the two Asian *Cinnamomum* sections (Sect. Camphora Meissn. and Sect. *Cinnamomum*) (Sun and Zhao, 1991; Dong et al., 2016; Huang et al., 2016). Hence, *C. chago* plays an important role in the phylogeny and evolution of *Cinnamomum*. Furthermore, *C. chago* is a potential source of timber and oil. Dong et al. (2016) detected that the *C. chago* seeds contain higher protein content (14.5%) and lower fat content (45.66%) than most nuts. However, this perennial species presents a restricted and fragmented distribution along the mountains upstream of the tributaries of Lancang River of Yunlong County, Yunnan province (Sun and Zhao, 1991; Dong et al., 2016). The transcriptomic information of *C. chago* is indispensable for identifying and understanding the biological processes involved in the accumulation of terpenoid and FA in this plant and its environmental adaptation.

Considering the endangered status and the large ecological value of *C. chago* in Lauraceae, we performed the transcriptome sequencing of *C. chago* leaves based on an Illumina HiSeq 4000 sequencing platform. This study aims to (1) characterize and analyze the functional annotation of the *C. chago* transcriptome, (2) identify candidate genes involved in terpenoid biosynthesis, FA biosynthesis, and response to abiotic stress, and (3) identify and validate gene-associated SSRs and SNPs. To the best of our knowledge, this study is the first to report a *de novo* transcriptome analysis on *C. chago*. Characterization of the genetic elements in terpenoid biosynthesis, FA biosynthesis, and response to abiotic stress and the development of gene-associated molecular markers will facilitate the conservation and sustainable utilization of the germplasm resources of *C. chago* and other Lauraceae species.

## Materials and Methods

### Plant Materials and RNA Extraction

Leaf samples were obtained from three *C. chago* seedlings that grew for 1 year in a greenhouse. The samples were dissected, immediately frozen in liquid nitrogen, and stored at −80°C until RNA extraction. According to the manufacturer’s instructions, total RNA was isolated using the TRIzol^®^ Reagent (Invitrogen, CA, United States) and purified using the Plant RNA Purification Reagent (Invitrogen, CA, United States) to remove the genomic DNA contamination. The RNA integrity was evaluated by agarose gel electrophoresis. The yield and purity of RNA were evaluated using a NanoDrop 2000 spectrophotometer (Thermo, MA, United States), with concentration higher than 50 ng/μL and 28S:18S higher than 1.8. The RNA integrity numbers were analyzed by Agilent 2100 (Agilent Technologies, CA, United States). Qualified RNA was subsequently used in the preparation of cDNA library and for Illumina deep sequencing.

### cDNA Library Construction and Sequencing

The mRNA-seq library was constructed using the TruseqTM RNA Sample Prep Kit (Illumina, CA, United States). The poly (A) mRNA was isolated using poly-T oligo-attached magnetic beads, mixed with the fragmentation buffer, and randomly broken into small pieces of 300 base pair (bp) by divalent cations under increased temperatures. The first-strand cDNA was synthesized by a random hexamer primer and reverse transcriptase. The second-strand cDNA was synthesized by RNase H and DNA polymerase I. Subsequently, the synthesized cDNA with cohesive terminus was resolved with EB buffer for end reparation, poly (A) addition, and sequencing adapter ligation. The obtained fragments were separated by agarose gel electrophoresis. Suitable sequencing templates were selected to generate cDNA libraries through polymerase chain reaction (PCR) amplification. Finally, the libraries were sequenced at the Major Company (Shanghai, China) using the Illumina HiSeq4000 sequencing platform to obtain 2 × 150 bp paired-end reads. The raw sequencing data and the assembled data were deposited in the NCBI Sequence Read Archive (SRA) database and the Transcriptome Shotgun Assembly (TSA) sequence database, respectively.

### Sequence Assembly and Annotation

Raw sequencing data were filtered by discarding contaminated adaptors, reads with excessive poly-N, reads of < 30 bp in length, empty reads, and reads with *Q* < 20 [*Q* = −10log10(E), E represents the sequencing error rate] using the SeqPrep program^1^ to screen high-quality clean read data for *de novo* assembly. The obtained clean data were *de novo* assembled using the Trinity program^2^ by default parameters, and the assembled contigs were further filtered and optimized using the TransRate^3^ and CD-HIT-EST^4^ programs. In addition, the Q20 and Q30 values, GC-content, and sequence duplication level of the clean data were calculated (Grabherr et al., 2011). The fragments per kilobase per million reads (FPKM) values were generated using RSEM v.1.2.9^5^.

The assembled unigenes were aligned by BLASTX against public databases (*E*-value < 1e-5), including Nr, String, Swiss-Prot, Kyoto Encyclopedia of Genes and Genomes (KEGG), and Pfam, to obtain the protein functional annotation and classification information (Camacho et al., 2009). The Gene Ontology (GO) annotation information of these unigenes was obtained and categorized with respect to biological process, molecular function, and cellular component by the Blast2GO program (Conesa et al., 2005). We also classified and analyzed these unigenes in the COG database^6^ according to the COG number obtained from String. The WEGO program (Ye et al., 2006) was used to classify all of the unigenes based on the GO annotation information. For removing possible contaminants from the transcriptome, we firstly filtered the contaminant sequences by BLAST searches and removed the non-green plant sequences (microbial and human contamination) based on the annotated categories in the KEGG and GO databases (Gray et al., 2010; Casseb et al., 2012). Secondly, we detected the candidate genes involved in terpenoid biosynthesis, FA biosynthesis, and response to abiotic stress, which are expressed in three of the libraries.

### Identification of SSRs and SNPs

Potential SSRs were detected using the MISA version 1.0 program^7^. The SSR parameters were designed to contain mononucleotides, dinucleotides, trinucleotides, tetranucleotides, pentanucleotides, and hexanucleotides with minimum repeat numbers of 10, 6, 5, 5, 5, and 5, respectively. The expressed sequence tag (EST)-SSR primers were designed using Primer3^8^ with default parameters. Later, the designed EST-SSR primers were synthesized by Sangon Company (Shanghai, China).

Candidate SNPs were detected using Samtools^9^ and VarScan V.2.2.7^10^ based on the following criteria: (1) minimum coverage of ten reads, (2) base quality where base calls show low Phred quality (<30), and (3) the frequency of mutated bases is higher than 30% among all reads covering the position.

Five accessions (each accession contains three individual plants) of *C. chago* obtained from Yunnan Province were selected to validate the putative SSRs and evaluate the polymorphism of related markers. The intact genomic DNA of each individual was extracted from dried leaves with the modified CTAB method (Doyle, 1991). The PCR reactions were referenced and modified into a total reaction volume of 25 μL, which contained DNA (15 ng), 10 × PCR buffer (2.5 μL), dNTPs (10 mM, 2 μL), primer (0.5 μL each), Taq DNA polymerase (5 U/μL, 0.5 μL; Takara, Shiga, Japan), and double-distilled water (18.5 μL). For each reaction, we used the following conditions: initial 5 min of denaturation at 94°C, 35 cycles of 30 s at 94°C, 30 s of annealing at Tm with different primers, 15 s of extension at 72°C, and a final extension for 7 min at 72°C. All purified PCR products of the EST-SSR marker were visualized on an ABI 3730xl Capillary DNA Analyzer (Sangon, Shanghai, China). The number of alleles and the observed heterozygosity (*Ho*) and expected heterozygosity (*He*) of all microsatellite loci were calculated by GenAlEx version 6.3 (Peakall and Smouse, 2006). The polymorphism information content (PIC) values were calculated by PIC_CALC version 0.6 (Nagy et al., 2012). The Hardy–Weinberg equilibrium (HWE) of all loci was determined by POPGENE version 1.31 (Yeh et al., 1997).

## Results and Discussion

### Sequence Analysis and *de novo* Assembly

To characterize the first transcriptome of *C. chago*, this study generated 56,891,547 raw reads from the constructed cDNA libraries (Supplementary Table S1). After a rigorous quality check and data filtering, 55,525,751 single clean reads with 98.29% Q20 bases (qualities that are larger than 20 for every base) were obtained, and these reads contained a large base number of 8,235,167,620 bp. The GC percentages for these clean reads were 48.57%. A total of 179,491 high-quality transcripts were assembled from clean reads, with a mean length of 628 bp and a N50 length of 1,025 bp (Table 1). Subsequently, these transcripts were attributed to alternative splicing of 129,097 unigenes with a mean length of 667 bp and a N50 length of 1,062 bp (Table 1). The lengths of the unigenes of *C. chago* are less than those of *N*. *sericea* (733 bp, Chen et al., 2015), *L*. *cubeba* (834 bp, Han et al., 2013), and *C. camphora* (680 bp, Shi et al., 2016b). This result may be due to the fact that most of the unigenes (90,287, 69.95%) exhibit lengths ranging from 1 to 500 bp (Supplementary Figure S1) and due to the absence of a well-assembled reference genome in *C. chago* (Zhou et al., 2016). This result provided sufficient and high-quality unigenes for the investigation of potential genes and identification of SSRs and SNPs in *C. chago*. Abundant candidate genes involved in a specific metabolic pathway and response to abiotic stress were identified. These transcriptome data will provide a basis for future studies on molecular biology, molecular breeding, physiology, and biochemistry, thereby, facilitating the protection and utilization of *C. chago* and other Lauraceae species.

**Table 1:** Summary statistics of *de novo* assembled transcriptome for *C. chago*.

Category	Items	Number
Raw data	Total raw reads	56,891,547
(Average)	Total raw base(bp)	8,590,623,647
Clean data	Total clean reads	55,525,751
(Average)	Total clean bases(bp)	8,235,167,620
	Error%	0.01
	Q20%	98.29
	Q30%	94.84
	GC%	48.57
Transcripts	Total number	179,491
	Smallest length(bp)	201
	Largest length(bp)	61,022
	Mean length (bp)	628
	N50 (bp)	1,025
Unigenes	Total number	129,097
	Smallest length(bp)	201
	Largest length(bp)	61,022
	Mean length (bp)	667
	N50 (bp)	1062

### Sequence Functional Annotation

**FIGURE 1:**
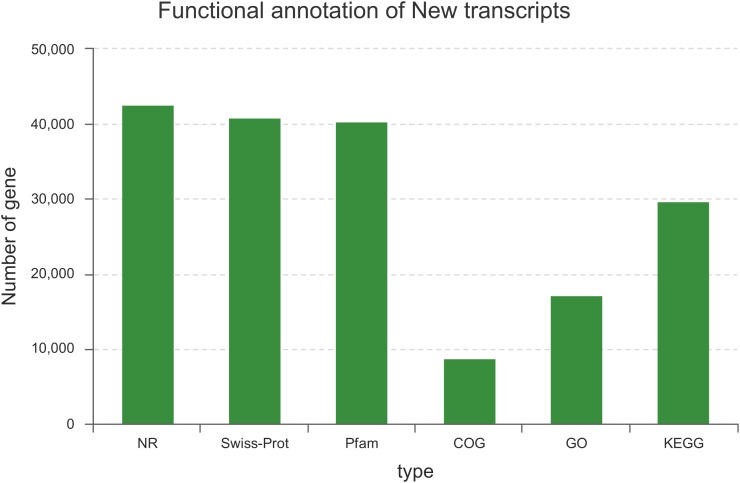
Summary statistics of functional annotations for the *C. chago* transcriptome in public databases.

Among the 129,097 unigenes, 56,887 (44.07%) unigenes were successfully annotated to at least one database (Figure 1). This ratio is comparable to those of other non-model organisms of Lauraceae, in which the percentage of annotated unigenes ranges from 38 to 56% (Han et al., 2013; Chen et al., 2015; Niu et al., 2015; Shi et al., 2016b). This result may be due to the fact that the unannotated unigenes represent untranslated mRNA regions, chimeric transcript sequences, non-conserved protein genes, assembly errors or specific novel genes to the species, and the different environmental stresses experienced by this species (Qiu et al., 2017; Horn et al., 2017).

### Nr Annotation

A total of 42,549 (74.79%) unigenes were annotated to the Nr protein database. Among them, the *E*-value distribution of the top hits in the Nr database revealed that 21,084 (49.55%) of the mapped sequences showed significant homology (less than 10^−30^) and 7,698 (18.09%) of the sequences with similarities between 10^−5^ and 10^−30^ (Supplementary Figure S2). For the species distribution, about 9,994 (23.78%), 3,349 (7.97%), and 2,616 (6.22%) unigenes were matched with *Nelumbo nucifera* (Nymphaeaceae), *Vitis vinifer*a (Vitaceae), and *Ricinus communis* (Euphorbiaceae), respectively. (Supplementary Figure S3).

### GO Classification

**FIGURE 2:**
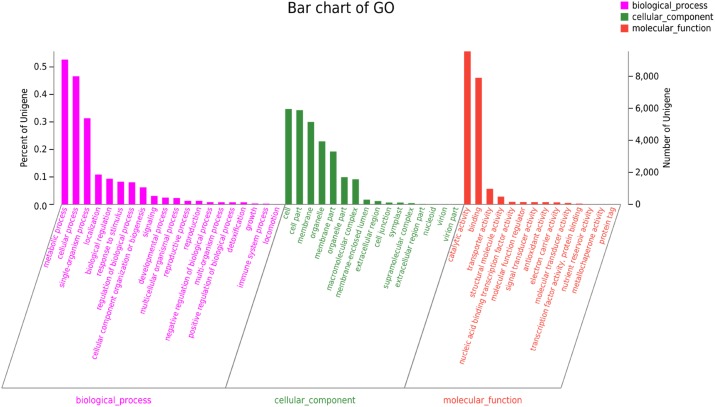
GO classification of the *C. chago* transcriptome.

The GO annotation was used to categorize the functions of the predicted *C. chago* unigenes (Gene Ontology Consortium, 2004). We categorized 17,166 (30.18%) unigenes to at least one main GO category, and these unigenes included 8,004 unigenes in cellular component, 9,043 unigenes in molecular function, and 32,193 unigenes in biological process (Figure 2). The distribution of the ontology categories was consistent with the transcriptomes of other plant species, such as *Tetrix japonica* (Qiu et al., 2017), *N*. *sericea* (Chen et al., 2015), *Vaccinium cyanococcus* (Rowland et al., 2012), and *Salix integra* (Shi et al., 2016a). This result demonstrated that most of the sequenced unigenes were responsible for the fundamental biological metabolism and composition of *C. chago* (Niu et al., 2015), such as cell, cell part, and membrane. Given that abundant unigenes were assigned with more than one GO term, the total number of assigned categories was larger than the total number of annotated unigenes (Qiu et al., 2017). Subsequently, these annotated unigenes were further grouped into 50 subcategories. For the molecular function, a majority of the annotated unique sequences were assigned to catalytic activity (9,560, 55.69%, GO: 0003824), binding (7,907, 46.06%, GO: 0005488) and transporter activity (973, 5.67%, GO: 0005215). For the cellular component, cell (5,955, 34.69%, GO: 0005623), cell part (5,888, 34.30%, GO: 0044464), and membrane (5150, 30.00%, GO: 0016020) were the most represented GO terms. In the biological process category, metabolic process (9,043, 52.68%, GO: 0008152), cellular process (8004, 46.63%, GO: 0009987), and single-organism process (5384, 31.36%, GO: 0044699) were the most represented categories. In addition, analysis of the top 20 subcategories of GO annotations showed that nine terms were from the biological process category, and seven terms were from the cellular component category (Figure 2).

### Complete Genome (COG) Classification

**FIGURE 3:**
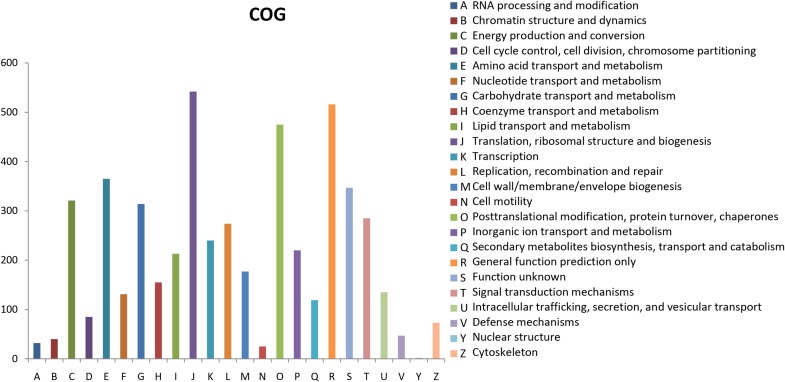
COG functional classification of the *C. chago* transcriptome.

The Clusters of Orthologous Groups of proteins (COGs) database for eukaryotes is used to classify genes based on orthologous relationships (Tatusov et al., 2003). A total of 8,701 (50.69%) unigenes were classified into 24 COG categories (Figure 3). Translation, ribosomal structure, and biogenesis (542, 6.23%); general function prediction only (516, 5.93%); and post-translational modification, protein turnover, and chaperones (475, 5.46%) were the top three terms among these categories. Nuclear structure term was the smallest classified group, which consisted of only two unigenes. The functional annotations correlated with cellular processes and signaling were identified; these annotations included defense mechanisms (47, 0.54%) and signal transduction mechanisms (285, 3.28%). For metabolism, the annotations were nucleotide transport and metabolism (131, 1.50%); coenzyme transport and metabolism (155, 1.78%); secondary metabolites biosynthesis, transport, and catabolism (119, 1.37%); inorganic ion transport and metabolism (220, 2.53%); lipid transport and metabolism (213, 2.45%); amino acid transport and metabolism (365, 4.19%); and carbohydrate transport and metabolism (314, 3.61%).

### KEGG Pathway

**FIGURE 4:**
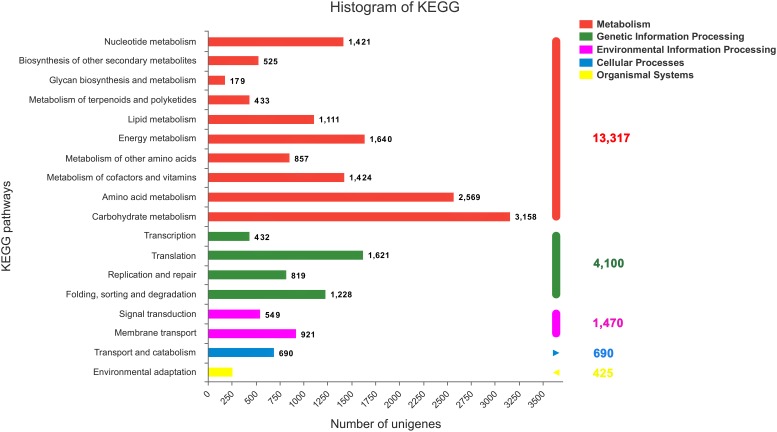
KEGG classification of the *C. chago* transcriptome (red) Metabolism, (green) Genetic Information Processing (pink) Environmental Information Processing (blue) Cellular Processes (yellow) Organismal Systems.

All the annotated sequences were mapped to the reference canonical pathways in the KEGG database to further identify the biological functions and interactions of genes in *C. chago* (Kanehisa and Goto, 2000). Consequently, a total of 29,604 (52.04%) unigenes were successfully mapped to 383 KEGG pathways. As shown in Figure 4, 13,317 (44.98%), 4,100 (13.85%), 1,470 (4.97%), 690 (2.33%), and 425 (1.44%) unigenes were classified to metabolism, genetic information processing, environmental information processing, cellular processes, and organismal systems, respectively. Carbohydrate metabolism (3,158, 23.71%), amino acid metabolism (2,569, 19.29%), and energy metabolism (1,640, 12.32%) were the predominant pathways in metabolism (Figure 4). In addition, purine metabolism (1,172, 3.95%, ko00230), ABC transporters (988, 3.34%, ko02010), ribosome (668, 2.26%, o03010), pyruvate metabolism (624, 2.11%, ko00620), pyrimidine metabolism (605, 2.04%, ko00240), oxidative phosphorylation (596, 2.01%, ko00190), and glycolysis/gluconeogenesis (590, 1.99%, ko00010) were the most abundant groups that were represented sequentially (Supplementary Table S2).

### Candidate Genes Related to Terpenoid Biosynthesis

Terpenoids are the largest secondary metabolites, and approximately 50,000 of them are structurally identified (Dixon, 2001). These metabolites are classified as monoterpenoids, sesquiterpenoids, diterpenoids, and triterpenoids according to the numbers of five-carbon precursors [isopentenyl diphosphate (IPP) and dimethylallyl pyrophosphate (DMAPP)] (Bohlmann et al., 1998). In *C. chago*, the putative genes identified in terpenoid biosynthesis were grouped into four parts: terpenoid backbone biosynthesis, sesquiterpenoid and triterpenoid biosyntheses, diterpenoid biosynthesis, and monoterpenoid biosynthesis. A total of 77 putative unigenes were homologous with 30 known genes in terpenoid backbone biosynthesis (Supplementary Table S3). Among them, a total of 10 candidate unigenes were annotated to code the key enzymes 1-deoxy-D-xylulose-5-phosphate synthase (K01662) and 1-deoxy-D-xylulose-5-phosphate reductoisomerase (K00099) in the MEP (methylerythritol 4-phosphate pathway), and the maximum mean FPKM values were 56.48 and 49.4 (Supplementary Table S4). Only five candidate unigenes were annotated to code the key enzymes hydroxymethylglutaryl-CoA synthase (K01641) and hydroxymethylglutaryl-CoA reductase (NAD^+^) (K00021) in cytoplasmic MVA (mevalonate pathway), and the maximum mean FPKM values were 29.39 and 18.16 (Supplementary Table S4). This result may indicate a more active MEP pathway than MVA pathway for terpenoid biosynthesis in *C. chago*.

Monoterpenes are the most dominant component of aromatic essential oil in Lauraceae species (Joshi et al., 2009; Han et al., 2013; Niogret et al., 2013). These compounds have been widely used as raw materials for cosmetics, pesticides, food additives, and biodiesel fuel (Kendra et al., 2014; Qiu et al., 2017). In the present study, six unigenes were annotated as homologous with three enzymes in monoterpenoid biosynthesis, including 3, 1, and 2 homologies of (-)-alpha-terpineol synthase (TES, K18108), (3S)-linalool synthase (LIS, K15086), and ( + )-neomenthol dehydrogenase (ND, K15095), respectively (Supplementary Table S3). These three enzymes were also detected in *L. glauca*, a species widely applied in essence and perfume, medicine, and chemical industry (Niu et al., 2015). Among them, TES and LIS catalyze the formations of linalool and alpha-terpineol, respectively (Aubourg et al., 2002). Neomenthol dehydrogenase plays an important role in resistance against microbial pathogens (e.g., bacteria, fungi, molds, or yeasts) in plants (Choi et al., 2008; Niu et al., 2015). Moreover, the sesquiterpenoid and triterpenoid biosyntheses and diterpenoid biosynthesis contained 13 and 20 unigenes that were homologous with six and eight known enzymes, respectively (Supplementary Table S3).

Terpenoids are important raw materials for flavors, fragrances, spices and as medicines against cancer, malaria, inflammation, and a variety of infectious diseases (Wang et al., 2005; Zhao et al., 2014). All the obtained candidate genes serve as an important basis for further exploration of the regulatory mechanism of *C. chago* terpenoid biosynthesis and provide references for the investigation of other aromatic plants.

### Candidate Genes Related to FA Biosynthesis

Plants represent a large reservoir of FA diversity and synthesize at least 200 different types of FAs (Van De Loo et al., 1995). Currently, most plant oils constituting FAs are used primarily as edible oils and as a renewable and easily extracted resource for a variety of industrial applications (e.g., biodiesel, paints, lubricants, coatings, or inks) (Lee et al., 1998; Simopoulos, 2001; He et al., 2013). Therefore, the factors limiting the accumulation of unusual FA in plants should be further understood (Thelen and Ohlrogge, 2002).

Most enzymes involved in lipid biosynthesis were identified in the *C. chago* transcriptome. A total of 65, 38, 21, and 60 unigenes were homologous with 15, 12, 4, and 15 known enzymes in FA biosynthesis, biosynthesis of unsaturated FAs (UFAs), linoleic acid (LA) metabolism, and alpha-linolenic acid (ALA) metabolism, respectively (Supplementary Table S5). Among these unigenes, one unigene (FPKM value was 5.02) was identified to encode the acetyl-CoA carboxylase (K11262; Supplementary Table S6), which is a regulatory enzyme controlling the rate of FA synthesis and catalyzing the first reaction to generate an intermediate malonyl-CoA (Li et al., 2015). This enzyme was also detected in *P. americana* and oil palm (Dussert et al., 2013; Kilaru et al., 2015).

Furthermore, desaturation steps are considered the rate-limiting steps for the biosynthesis of UFAs (Leonard et al., 2004). Fatty acid desaturase (FAD) is responsible for the sequential modification in this step to generate LA (C18:2n-6) and ALA (C18:3n-3) (Leonard et al., 2004; Li et al., 2015). In *C. chago*, we detected four FADs, which contained acyl-[acyl-carrier-protein] desaturase (DESA1, four unigenes, maximum mean FPKM = 188.99, K03921), acyl-lipid omega-3 desaturase (FAD8/desB, four unigenes, maximum mean FPKM = 6.29, K10257), delta-12 desaturase (FAD2, two unigenes, maximum mean FPKM = 22.51, K10256), and acyl-lipid omega-6 desaturase/delta-12 desaturase (FAD6/desA, one unigene, mean FPKM = 61.47, K10255; Supplementary Tables S5, S6). Among them, FAD2 and FAD8 have been reported to exhibit a higher expression level in fast oil accumulation stage than that in the initial stage of seed development in tree peony seeds (Ohlrogge and Browse, 1995; Li et al., 2015). In addition, 21 unigenes were identified to encode long-chain acyl-CoA synthetases (ACSL/fadD, maximum mean FPKM = 42.67, K01897), which catalyze the initial condensation step to generate the endoplasmic reticulum (ER) acyl-CoA pool (Leonard et al., 2004; Li et al., 2015).

In conclusion, results showed that an active FA biosynthesis pathway caused by abundant key related genes was detected in *C. chago*. Considering the crucial role of the obtained candidate genes, further studies focusing on the identification of factors and encoding functional genes would probably reveal the molecular mechanisms underlying FA biosynthesis in *C*. *chago* and other related species.

### Candidate Genes Related to Abiotic Stress

In this study, abundant potential unigenes were homologous with known genes in response to water deprivation (387 unigenes, 40 genes), cold (498 unigenes, 72 genes), and heat (317 unigenes, 46 genes; Supplementary Tables S7, S9, S11). In response to water deprivation, aquaporin PIP (PIP, c125607_g1_i1, mean FPKM = 864.43, K00799), glutathione S-transferase (Gst, c100118_g1_i1, mean FPKM = 732.86, K14638), and EREBP-like factor (c100057_g1_i1, mean FPKM = 400.68, K09286) were the predominant enzymes that were detected sequentially (Supplementary Table S8). In response to cold, the most representative enzyme was ribulose-bisphosphate carboxylase small chain (c62606_g2_i1, mean FPKM = 12247.08, K09286), sequentially followed by carbonic anhydrase (c90492_g1_i1, mean FPKM = 2002.51, K01673) and thiamine thiazole synthase (c81250_g1_i1, FPKM = 1904.41, K03146) (Supplementary Table S10). In response to heat, the top four identified enzymes were glyceraldehyde 3-phosphate dehydrogenase (c92588_g1_i1, mean FPKM = 1442.93, K00134), DnaJ homolog subfamily A member 2 (c87266_g1_i1, mean FPKM = 575.69, K09503), EREBP-like factor (c100057_g1_i1, mean FPKM = 400.68, K09286), and GDP-L-galactose phosphorylase (c62967_g1_i1, mean FPKM = 389.79, K14190; Supplementary Table S12). We also identified a few unigenes that responded to other abiotic stress, and these unigenes included 2, 2, 18, 37, and 3 unigenes that responded to herbicide, nitrosative stress, red or far-red light, pH, and anoxia, respectively (Supplementary Tables S13, S14). Additionally, many unigenes were homologous to the same few genes, such as Gst (28 unigenes), EREBP (32 unigenes), CML (33 unigenes), SLC15A3_4/PHT (36 unigenes), heat shock 70kDa protein 1/8 (32 unigenes), etc. These results may be caused by the sequenced fragmented transcripts that are typically distributed in the range of 0–500 bp (Supplementary Figure S1; Mantione et al., 2014).

We also discovered that 22 identified genes existed in multiple-stress responses in *C. chago* (Table 2). The EREBP-like factor (EREBP, K09286), heat shock protein 90kDa beta (HSP90B/TRA1, K09487), and annexin A7/11 (ANXA7_11, K17095) were implicated in water deprivation, cold, and heat simultaneously. A total of 12 genes were both assigned to water deprivation and cold stress response, of which Gst, phospholipase D1/2 (K01115), and sucrose synthase (K00695) were the most abundant enzymes. Moreover, five genes synchronously participated in cold and heat stress responses. The heat shock 70kDa protein 1/8, calcium-binding protein CML, and chaperonin GroEL (groEL/HSPD1, K04077) were the top three genes. The solute carrier family 15 (peptide/histidine transporter), member 3/4 (SLC15A3_4/PHT) was the only gene that concurrently existed in the process of water deprivation and pH stress response (Table 2).

**Table 2:** Candidate genes of the *C. chago* transcriptome simultaneously involved in the response to abiotic stress.

KO ID	Gene	KEGG Annotation	Numbers unigenes	Abiotic stress
K00799	GST, gst	Glutathione S-transferase	28	Cold, water deprivation
K14638	SLC15A3_4, PHT	Solute carrier family 15 (peptide/histidine transporter), member 3/4	34	Water deprivation, pH
K03283	HSPA1_8	Heat shock 70 kDa protein 1/8	32	Cold, heat
K13448	CML	Calcium-binding protein CML	33	Cold, heat
K09286	EREBP	EREBP-like factor	32	Water deprivation, cold, heat
K04077	groEL, HSPD1	Chaperonin GroEL	16	Cold, heat
K01115	PLD1_2	Phospholipase D1/2	19	Water deprivation, cold
K00695	E2.4.1.13	Sucrose synthase (SUS)	9	Water deprivation, cold
K09487	HSP90B, TRA1	Heat shock protein 90kDa beta	12	Water deprivation, cold, heat
K17279	REEP5_6	Receptor expression-enhancing protein 5/6	9	Water deprivation, cold
K01177	E3.2.1.2	Beta-amylase	8	Water deprivation, cold
K17095	ANXA7_11	Annexin A7/11	8	Water deprivation, cold, heat
K14803	PTC2_3	Protein phosphatase PTC2/3	10	Water deprivation, cold
K09250	CNBP	Cellular nucleic acid-binding protein	8	water deprivation, cold
K16911	DDX21	ATP-dependent RNA helicase DDX21	3	water deprivation, cold
K17679	MSS116	ATP-dependent RNA helicase MSS116, mitochondrial	3	Water deprivation, cold
K06268	PPP3R, CNB	Serine/threonine-protein phosphatase 2B regulatory subunit	5	Water deprivation, cold
K12885	RBMX, HNRNPG	Heterogeneous nuclear ribonucleoprotein G	4	Water deprivation, cold
K03627	MBF1	Putative transcription factor	2	Water deprivation, heat
K03098	APOD	Apolipoprotein D and lipocalin family protein	2	Cold, heat
K17991	PXG	Peroxygenase	2	Water deprivation, cold
K04688	RPS6KB	Ribosomal protein S6 kinase beta	1	Cold, heat

In general, the plant multiple-stress tolerance mechanism consists of a complex network by which several pathways overlap and interact with one another (Timperio et al., 2008). Among the 22 candidate genes, the interaction mechanism of some genes referring to abiotic stress, such as GST, HSP, and EREBP, was comprehensively studied in other species (Dietz et al., 2010; Kumar et al., 2013; Zhu et al., 2014). However, in *C. chago*, even in Lauraceae, the role of these putative genes in plant growth, development, and responses to environmental stresses is unknown. Investigation on these candidate genes related to abiotic stress will further facilitate our understanding of the genetic basis of adaptation and provide information to the growing body of research on the ecological and evolutionary consequences of environment in *C. chago* and other Lauraceae species.

### Identification of SNPs and SSRs

The SSRs and SNPs, representing uncorrelated patterns of diversity and divergence, exhibit the advantage of being highly polymorphic, codominant, highly reproducible, stable, and reliable (DeFaveri et al., 2013). Transcriptome sequencing on multiple individuals is an effective way to identify and validate SNPs and SSRs (Huang et al., 2015). The identification and validation of gene-associated SSRs and SNPs will provide an important tool in understanding the genetic basis of adaptive trait, population genetic structuring, and species relatedness for *C. chago* and other related species in Lauraceae (Chen et al., 2015; Huang et al., 2015; Jardine et al., 2016).

### SSRs

A total of 25,654 potential EST-SSRs and 3,208 sequences containing more than one EST-SSR locus were identified (Table 3). Subsequently, the type and distribution of these EST-SSRs were investigated and calculated. Results showed that the motif type of identified SSRs mainly consisted of mononucleotide (14,914, 58.14%), dinucleotide (6,731, 26.24%), trinucleotide (3,776, 14.72%), tetranucleotide (194, 0.76%), hexanucleotide (20, 0.08%), and pentanucleotide (17, 0.07%; Supplementary Figure S4) repeats. This result was in contrast to those of previous studies on *N*. *sericea* and *L*. *glauca* in Lauraceae (Chen et al., 2015; Zhu et al., 2018). This result may be attributed to the overexpression of untranslated regions (UTRs), in which mononucleotide, dinucleotide, and tetranucleotide repeats mainly occur (Kumpatla and Mukhopadhyay, 2005). Among the identified SSRs, a total of 78 motif sequence types, including 2, 4, 10, 25, 20, and 17 types of monorepeats, direpeats, trirepeats, tetrarepeats, hexarepeats, and pentarepeats, respectively, were identified. The most dominant monorepeat was A/T (14,737, 57.44%). In addition, AG/CT (4,737, 18.46%) and AAG/CTT (1,383, 5.39%) were the most abundant direpeat and trirepeat, respectively (Supplementary Table S15). These results supported the previous observations that A/T, AG/CT, and AAG/CTT repeats are the most abundant SSR motifs in dicots (Chen et al., 2015; Zhu et al., 2018). Moreover, the most common repeat motifs in all EST-SSRs were 6–10 tandem repeats (13,006, 50.70%), followed by 11–15 tandem repeats (7,941, 30.95%) and 1–5 tandem repeats (2,409, 9.39%). Tandem repeats of more than 15 repeats were 2, 298 (8.96%; Table 4).

**Table 3:** Summary statistics of EST-SSRs and ESE-SNPs identified from the transcriptome of *C. chago*.

EST-SSRs	Total number of identified SSRs	25,654
	Total size of examined sequences (bp)	84,676,396
	Number of SSR-containing sequences	20,307
	Number of sequences containing more than one SSR	3,208

EST-SNPs	Number of SNPs	640

	SNP frequency per Kb	0.01
	Transition	396
	A/G	202
	C/T	194
	Transversion	244
	A/C	63
	A/T	74
	C/G	44
	G/T	63

**Table 4:** Distribution of EST-SSRs based on motif types and nucleotide repeat units in *C. chago*.

SSR type	Repeat number (1–5)	Repeat number (6–10)	Repeat number (11–15)	Repeat number (>15)
**Mono-nucleotide**				
A/T	0	4826	7664	2247
C/G	0	35	92	50
**Di-nucleotide**				
AC/GT	0	656	27	0
AG/CT	0	4641	96	0
AT/AT	0	1198	59	1
CG/CG	0	53	0	0
**Tri-nucleotide**				
AAC/GTT	127	86	0	0
AAG/CTT	772	611	0	0
AAT/ATT	211	201	0	0
ACC/GGT	136	92	1	0
ACG/CGT	37	22	0	0
ACT/AGT	23	15	0	0
AGC/CTG	202	139	0	0
AGG/CCT	233	171	0	0
ATC/ATG	385	194	0	0
CCG/CGG	81	37	0	0
**Tetra-nucleotide**				
AAAC/GTTT	12	2	0	0
AAAG/CTTT	35	4	0	0
AAAT/ATTT	48	5	0	0
AACC/GGTT	1	0	0	0
AACG/CGTT	1	0	0	0
AAGC/CTTG	1	0	0	0
AAGG/CCTT	8	1	0	0
AATC/ATTG	7	0	0	0
AATG/ATTC	1	0	0	0
AATT/AATT	2	0	0	0
ACAG/CTGT	1	0	0	0
ACAT/ATGT	10	1	0	0
ACCC/GGGT	1	0	0	0
ACGC/CGTG	2	0	0	0
ACGG/CCGT	0	1	0	0
ACTC/AGTG	1	0	0	0
ACTG/AGTC	1	0	0	0
AGAT/ATCT	18	2	1	0
AGCC/CTGG	1	0	0	0
AGCG/CGCT	2	1	0	0
AGGC/CCTG	2	0	0	0
AGGG/CCCT	13	1	0	0
ATCC/ATGG	4	1	0	0
ATCG/ATCG	1	0	0	0
ATGC/ATGC	1	0	0	0
**Penta-nucleotide**				
AAAAG/CTTTT	0	0	1	0
AAACG/CGTTT	0	1	0	0
AAATC/ATTTG	1	0	0	0
AAGGG/CCCTT	1	0	0	0
AAGTG/ACTTC	1	0	0	0
AATAT/ATATT	2	0	0	0
AATCG/ATTCG	1	0	0	0
AATCT/AGATT	1	0	0	0
ACACG/CGTGT	1	0	0	0
ACAGG/CCTGT	1	0	0	0
ACCCG/CGGGT	1	0	0	0
ACTCC/AGTGG	1	0	0	0
ACTCT/AGAGT	2	0	0	0
AGAGG/CCTCT	1	0	0	0
AGCCC/CTGGG	1	0	0	0
AGGCG/CCTCG	1	0	0	0
CCCCG/CGGGG	1	0	0	0
**Hexa-nucleotide**				
AAAACG/CGTTTT	1	0	0	0
AAACAG/CTGTTT	0	1	0	0
AAAGAG/CTCTTT	0	1	0	0
AAAGAT/ATCTTT	1	0	0	0
AAATGC/ATTTGC	1	0	0	0
AACCCC/GGGGTT	1	0	0	0
AACCTG/AGGTTC	1	0	0	0
AAGGAG/CCTTCT	0	1	0	0
AAGGTG/ACCTTC	0	1	0	0
AAGTGG/ACTTCC	1	0	0	0
AATAGT/ACTATT	0	1	0	0
AATATG/ATATTC	0	1	0	0
AATGAG/ATTCTC	0	1	0	0
ACACAT/ATGTGT	1	0	0	0
ACCATC/ATGGTG	1	0	0	0
ACGAGG/CCTCGT	1	0	0	0
AGATGG/ATCTCC	1	0	0	0
AGCAGG/CCTGCT	0	1	0	0
ATCGCC/ATGGCG	1	0	0	0
ATCGGC/ATGCCG	0	1	0	0
**Total**	2409	13006	7941	2298
**Percentage (%)**	9.39%	50.70%	30.95%	8.96%

According to the putative EST-SSRs, we randomly selected and designed 100 primer pairs to synthesize and evaluate their ability to amplify and assess polymorphism in *C. chago*. Among these EST-SSR primers, 85 primers successfully amplified PCR products. The 15 remaining primer pairs failed to generate PCR amplification or amplified remarkably weak bands at various annealing temperatures. Among the 85 successful primers, 60 primers presented the expected correct size of amplifications, 16 of them were longer or shorter than the expected size, and 9 primer pairs generated significantly multiple bands. Finally, 55 of the 60 validated primers were polymorphic among the five *C. chago* accessions, and the five remaining primers were monomorphic (Supplementary Table S15).

Diversity estimation from microsatellites showed that a total of 329 alleles were obtained from the 55 novel EST-SSR primers. The number of alleles ranged from 2 to 13 with an average of 5.982 per locus. The *H_O_* and *H_E_* values were in the range of 0.00–1.00 (mean, 0.759) and 0.111–0.761 (mean, 0.519), respectively. The PIC values ranged from 0.124 to 0.872, with a mean value of 0.570. Furthermore, 14 loci showed PIC values that were smaller than 0.50, and 41 loci showed PIC values that were larger than 0.50 (Supplementary Table S15). The putative EST-SSR loci in *C. chago* obtained by the novel EST-SSR markers were more highly polymorphic than those in other Lauraceae species.

### SNPs

We obtained a total of 640 putative EST-SNPs with the frequency per kb of 0.01 in *C. chago* (Table 3). Among them, transition SNPs were the most predominant, of which 396 (61.87%) SNPs were identified; these SNPs contained 202 A/G (31.56%) and 194 C/T (30.31%) (Table 3). Additionally, the most common base variations were A/T (74; 11.56%) in transversion SNPs, sequentially followed by G/T (63; 9.84%) and A/C (63; 9.84%) (Table 3). This result was consistent with the conclusion that transition mutations are better tolerated than transversions because their synonymous mutations are generated in protein-coding sequences during natural selection (Mantello et al., 2014).

The estimated locations were obtained for 291 of the total 640 SNPs. The remaining locations were uncertain because they extended over both estimated coding and non-coding regions. Most SNPs (145, 23.00%) occurred frequently in the third codon regions (Supplementary Figure S5), which may be due to selective pressures on SNPs in the coding regions (Li et al., 2013).

## Conclusion

Considerably valuable transcriptome sequencing data and annotation resources were developed for *C. chago* using the RNA-seq technology. A total of 129,097 unigenes with a mean length of 667 bp and a N50 length of 1,062 bp were assembled from 55,525,751 clean reads with 98.29% Q20 bases. Among these unigenes, only 56,887 (44.07%) unigenes were successfully annotated using at least one database; these unigenes contained 40,323 unigenes (31.23%) for Pfam, 40,810 unigenes (31.61%) for Swiss-Prot, 29,660 unigenes (49.47%) for KEGG, 8,701 unigenes (22.97%) for COG databases, 17,166 unigenes (13.30%) for GO databases, and 42,549 unigenes (32.96%) for the Nr database. Furthermore, a total of 116 unigenes with 47 candidate genes and 184 unigenes with 46 candidate genes were identified in terpenoid backbone biosynthesis and FA biosynthesis, respectively. A large number of putative genes related to abiotic stress were also identified in *C. chago*. Among these genes, 22 candidate genes participated in at least one stress response. In addition, 25,654 SSRs and 640 SNPs were identified, respectively. A total of 55 novel EST-SSR primers were also successfully developed for *C. chago*. These assembled transcriptome data and annotation information can be used to establish a valuable information platform for future research on genetic and genomic mechanisms underlying the specific metabolic pathway and germplasm conservation and utilization in *C. chago*.

## Data Accessibility

Raw sequence reads were deposited in the Short Read Archive (SRA) (http://www.ncbi.nlm.nih.gov/sra) under BioProject PRJNA387488 and SRA Accession No. SRP107900 (SRS2220410; SRS2220411; SRS2220412). Assembled data have been deposited in TSA Database with the above BioProject identification number.

## Author Contributions

S-KS, XZ, and Y-HW designed the study. S-KS and Y-HW obtained the funding. XZ, S-KS, and Y-HW performed the fieldwork and seedling propagation. XZ, YZ, and S-KS performed the laboratory work and analyzed the data. XZ and S-KS wrote and revised the manuscript. All authors read and approved the manuscript.

## Conflict of Interest Statement

The authors declare that the research was conducted in the absence of any commercial or financial relationships that could be construed as a potential conflict of interest.

## Footnotes

1 https://github.com/jstjohn/SeqPrep

2 http://trinityrnaseq.sourceforge.net/

3 http://hibberdlab.com/transrate

4 http://weizhongli-lab.org/cd-hit

5 http://deweylab.github.io/RSEM/

6 http://www.ncbi.nlm.nih.gov/COG/

7 http://pgrc.ipk-gatersleben.de/misa/

8 https://wheat.pw.usda.gov/demos/BatchPrimer3/

9 http://samtools.sourceforge.net/

10 http://varscan.sourceforge.net/
