# Supplementary material for: Transcriptome Analysis of Cinnamomum chago: A Revelation of Candidate Genes for Abiotic Stress Response and Terpenoid and Fatty Acid Biosyntheses
Source: Front Genet. 2018 Nov 5;9:505. doi: 10.3389/fgene.2018.00505 (PMC6231050; doi:10.3389/fgene.2018.00505)
Supplement: Supplementary file 5 [file Image_5.PDF]

Supplementary Material

Characterization of the de novo Cinnamomum chago (Lauraceae) transcriptome reveals candidate genes for terpenoid, fatty acid biosyntheses and abiotic stress

Authors: Xue Zhang, Shi-Kang Shen *,

***Address for Correspondence:** Shen Shikang, School of Life Sciences, Yunnan University, No. 2 Green lake North road Kunming, Yunnan, 650091, the People's Republic of China. Telephone:+86-871-65031412; Fax:+86-871-65031412;

E-mail: yunda123456@126.com

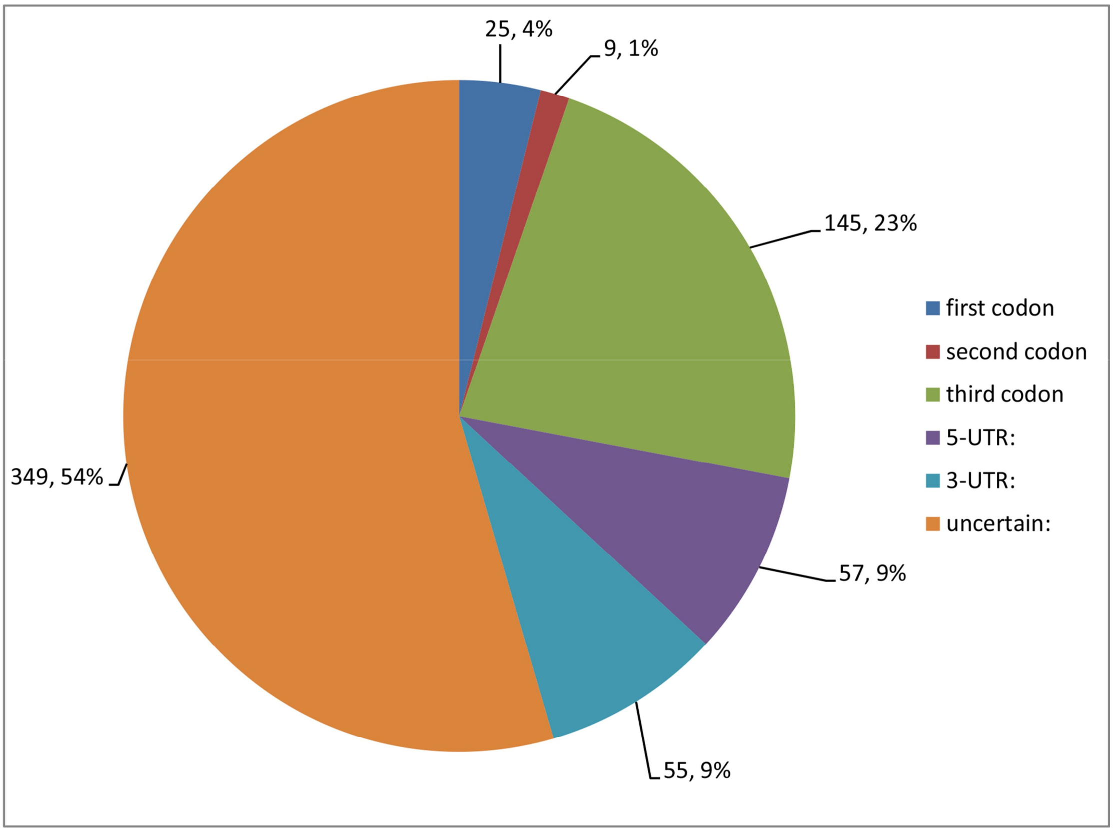

Supplementary Figure 5 Classification of the SNPs position for *C. chago*
