# Supplementary material for: Transcriptome Analysis of Cinnamomum chago: A Revelation of Candidate Genes for Abiotic Stress Response and Terpenoid and Fatty Acid Biosyntheses
Source: Front Genet. 2018 Nov 5;9:505. doi: 10.3389/fgene.2018.00505 (PMC6231050; doi:10.3389/fgene.2018.00505)
Supplement: Supplementary file 6 [file Table_1.DOC]

***Supplementary Material***

**Characterization of the de novo *Cinnamomum chago* (Lauraceae) transcriptome reveals candidate genes for terpenoid, fatty acid biosyntheses and abiotic stress**

**Authors:** Xue Zhang, Shi-Kang Shen *,

***Address for Correspondence:** Shi-Kang Shen, School of Life Sciences, Yunnan University, No. 2 Green lake North road Kunming, Yunnan, 650091, the People’s Republic of China. Telephone:+86-871-65031412; Fax:+86-871-65031412;

**E-mail:** yunda123456@126.com

**Table S1 Raw datas of transcriptome for *C. chago***

| **Sample** | **Ccg1** | **Ccg2** | **Ccg3** |
| --- | --- | --- | --- |
| Raw reads | 57,111,990 | 63,737,166 | 49,825,486 |
| Raw bases | 8,623,910,490 | 9,624,312,066 | 7,523,648,386 |
| Clean reads | 55,800,792 | 62,451,966 | 48,482,870 |
| Clean bases | 8,279,299,923 | 9,271,092,920 | 7,171,160,584 |
| Error rate(%) | 0.01% | 0.01% | 0.01% |
| Q20(%) | 98.34% | 98.38% | 98.11% |
| Q30(%) | 94.98% | 95.08% | 94.4% |
| GC content(%) | 49.11% | 48.00% | 48.59% |
