# Supplementary material for: Transcriptome Analysis of Cinnamomum chago: A Revelation of Candidate Genes for Abiotic Stress Response and Terpenoid and Fatty Acid Biosyntheses
Source: Front Genet. 2018 Nov 5;9:505. doi: 10.3389/fgene.2018.00505 (PMC6231050; doi:10.3389/fgene.2018.00505)
Supplement: Supplementary file 7 [file Table_2.DOC]

***Supplementary Material***

**Characterization of the de novo *Cinnamomum chago* (Lauraceae) transcriptome reveals candidate genes for terpenoid, fatty acid biosyntheses and abiotic stress**

**Authors:** Xue Zhang, Shi-Kang Shen *

***Address for Correspondence:** Shi-Kang Shen, School of Life Sciences, Yunnan University, No. 2 Green lake North road Kunming, Yunnan, 650091, the People’s Republic of China. Telephone:+86-871-65031412; Fax:+86-871-65031412;

**E-mail:** yunda123456@126.com

**Table S2 Top 20 KEGG annotations of *C. chago* transcriptome**

| Pathway | Pathway definition | Number of seqs |
| --- | --- | --- |
| ko00230 | Purine metabolism | 1172 |
| ko02010 | ABC transporters | 921 |
| ko03010 | Ribosome | 668 |
| ko00620 | Pyruvate metabolism | 624 |
| ko00240 | Pyrimidine metabolism | 605 |
| ko00190 | Oxidative phosphorylation | 596 |
| ko00010 | Glycolysis / Gluconeogenesis | 590 |
| ko00630 | Glyoxylate and dicarboxylate metabolism | 561 |
| ko00500 | Starch and sucrose metabolism | 454 |
| ko00520 | Amino sugar and nucleotide sugar metabolism | 429 |
| ko00260 | Glycine, serine and threonine metabolism | 424 |
| ko00270 | Cysteine and methionine metabolism | 418 |
| ko00640 | Propanoate metabolism | 399 |
| ko00970 | Aminoacyl-tRNA biosynthesis | 391 |
| ko04141 | Protein processing in endoplasmic reticulum | 389 |
| ko00280 | Valine, leucine and isoleucine degradation | 371 |
| ko04626 | Plant-pathogen interaction | 361 |
| ko00020 | Citrate cycle (TCA cycle) | 358 |
| ko00250 | Alanine, aspartate and glutamate metabolism | 355 |
| ko00860 | Porphyrin and chlorophyll metabolism | 339 |
