# Supplementary material for: Transcriptome Analysis of Cinnamomum chago: A Revelation of Candidate Genes for Abiotic Stress Response and Terpenoid and Fatty Acid Biosyntheses
Source: Front Genet. 2018 Nov 5;9:505. doi: 10.3389/fgene.2018.00505 (PMC6231050; doi:10.3389/fgene.2018.00505)
Supplement: Supplementary file 8 [file Table_3.DOC]

***Supplementary Material***

**Characterization of the de novo *Cinnamomum chago* (Lauraceae) transcriptome reveals candidate genes for terpenoid, fatty acid biosyntheses and abiotic stress**

**Authors:** Xue Zhang, Shi-Kang Shen *,

***Address for Correspondence:** Shi-Kang Shen, School of Life Sciences, Yunnan University, No. 2 Green lake North road Kunming, Yunnan, 650091, the People’s Republic of China. Telephone:+86-871-65031412; Fax:+86-871-65031412;

**E-mail:** yunda123456@126.com

**Table S3** Candidate genes involved in Terpenoid biosynthesis pathway

| **KO ID** | **KEGG Annotation** | **Gene ID** | **Numbers of unineges** |
| --- | --- | --- | --- |
| **Terpenoid backbone biosynthesis** | | | |
| K00626 | acetyl-CoA C-acetyltransferase | E2.3.1.9, atoB | 7 |
| K01662 | 1-deoxy-D-xylulose-5-phosphate synthase | dxs | 9 |
| K10960 | geranylgeranyl diphosphate | chlP, bchP | 3 |
| K11778 | ditrans,polycis-polyprenyl diphosphate synthase | DHDDS, RER2, SRT1 | 7 |
| K03527 | 4-hydroxy-3-methylbut-2-en-1-yl diphosphate reductase | ispH, lytB | 1 |
| K03526 | (E)-4-hydroxy-3-methylbut-2-enyl-diphosphate synthase | gcpE, ispG | 4 |
| K00099 | 1-deoxy-D-xylulose-5-phosphate reductoisomerase | dxr | 1 |
| K13789 | geranylgeranyl diphosphate synthase, type II | GGPS | 7 |
| K00021 | hydroxymethylglutaryl-CoA reductase (NADPH) | HMGCR | 3 |
| K05356 | all-trans-nonaprenyl-diphosphate synthase | SPS, sds | 4 |
| K00919 | 4-diphosphocytidyl-2-C-methyl-D-erythritol kinase | ispE | 1 |
| K14066 | geranyl diphosphate synthase | GPS | 1 |
| K06013 | STE24 endopeptidase | STE24 | 1 |
| K12506 | 2-C-methyl-D-erythritol 4-phosphate cytidylyltransfera | ispDF | 1 |
| K15892 | farnesol kinase | FOLK | 5 |
| K00991 | 2-C-methyl-D-erythritol 4-phosphate cytidylyltransferase | ispD | 1 |
| K00787 | farnesyl diphosphate synthase | FDPS | 3 |
| K00869 | mevalonate kinase | MVK, mvaK1 | 2 |
| K01770 | 2-C-methyl-D-erythritol 2,4-cyclodiphosphate synthase | ispF | 1 |
| K01823 | isopentenyl-diphosphate Delta-isomerase | idi, IDI | 3 |
| K08658 | prenyl protein peptidase | RCE1, FACE2 | 1 |
| K15889 | prenylcysteine alpha-carboxyl methylesterase | PCME | 1 |
| K01641 | hydroxymethylglutaryl-CoA synthase | E2.3.3.10 | 2 |
| K15891 | NAD+-dependent farnesol dehydrogenase | FLDH | 2 |
| K00587 | protein-S-isoprenylcysteine O-methyltransferase | ICMT, STE14 | 1 |
| K00938 | phosphomevalonate kinase | E2.7.4.2, mvaK2 | 1 |
| K01597 | diphosphomevalonate decarboxylase | MVD, mvaD | 1 |
| K05906 | prenylcysteine oxidase | PCYOX1, FCLY | 1 |
| K05954 | protein farnesyltransferase subunit beta | FNTB | 1 |
| K05955 | protein farnesyltransferase | FNTA | 1 |
| Total |  | 30 | 77 |
| **Sesquiterpenoid and triterpenoid biosynthesis** | | | |
| K15803 | (-)-germacrene D synthase | GERD | 3 |
| K14181 | valencene/7-epi-alpha-selinene synthase | TPS1 | 1 |
| K00511 | squalene monooxygenase | SQLE, ERG1 | 4 |
| K00801 | farnesyl-diphosphate farnesyltransferase | FDFT1 | 1 |
| K15813 | beta-amyrin synthase | LUP4 | 2 |
| K15891 | NAD+-dependent farnesol dehydrogenase | FLDH | 2 |
| Total |  | 6 | 13 |
| **Diterpenoid biosynthesis** | | | |
| K04125 | gibberellin 2-oxidase | E1.14.11.13 | 4 |
| K04120 | ent-copalyl diphosphate synthase | E5.5.1.13 | 3 |
| K17982 | geranyllinalool synthase | TPS04, GES | 5 |
| K04122 | ent-kaurene oxidase | GA3, CYP701 | 3 |
| K04121 | ent-kaurene synthase | E4.2.3.19 | 2 |
| K04124 | gibberellin 3-beta-dioxygenase | E1.14.11.15 | 1 |
| K04123 | ent-kaurenoic acid hydroxylase | KAO | 1 |
| K05282 | gibberellin 20-oxidase | E1.14.11.12 | 1 |
| Total |  | 8 | 20 |
| **Monoterpenoid biosynthesis** | | | |
| K18108 | (-)-alpha-terpineol synthase | E4.2.3.111 | 3 |
| K15086 | (3S)-linalool synthase | TPS14 | 1 |
| K15095 | (+)-neomenthol dehydrogenase | E1.1.1.208 | 2 |
| Total |  | 3 | 6 |
| all together |  | 47 | 116 |
