# Supplementary material for: Transcriptome Analysis of Cinnamomum chago: A Revelation of Candidate Genes for Abiotic Stress Response and Terpenoid and Fatty Acid Biosyntheses
Source: Front Genet. 2018 Nov 5;9:505. doi: 10.3389/fgene.2018.00505 (PMC6231050; doi:10.3389/fgene.2018.00505)
Supplement: Supplementary file 9 [file Table_4.DOC]

***Supplementary Material***

**Characterization of the de novo *Cinnamomum chago* (Lauraceae) transcriptome reveals candidate genes for terpenoid, fatty acid biosyntheses and abiotic stress**

**Authors:** Xue Zhang, Shi-Kang Shen *,

***Address for Correspondence:** Shi-Kang Shen, School of Life Sciences, Yunnan University, No. 2 Green lake North road Kunming, Yunnan, 650091, the People’s Republic of China. Telephone:+86-871-65031412; Fax:+86-871-65031412;

**E-mail:** yunda123456@126.com

**Table S4 The FPKM values of candidate genes involved in Terpenoid biosynthesis pathway**

| **KO ID** | **KEGG annotation** | **Unigene** | **Ccg1** | **Ccg2** | **Ccg3** | **Mean** |
| --- | --- | --- | --- | --- | --- | --- |
| **Terpenoid backbone biosynthesis** | |  |  |  |  |  |
| K00626 | acetyl-CoA C-acetyltransferase | c73958_g1_i1 | 10.57 | 8.34 | 7.44 | 8.78 |
|  |  | c77710_g1_i1 | 10.44 | 9.37 | 18.65 | 12.82 |
|  |  | c93178_g2_i1 | 0.84 | 0.12 | 0.62 | 0.53 |
|  |  | c96279_g1_i1 | 19.62 | 20.55 | 20.28 | 20.15 |
|  |  | c13870_g1_i1 | 0.24 | 0.39 | 0.53 | 0.37 |
|  |  | c51187_g1_i1 | 4.79 | 11.59 | 9.53 | 8.64 |
|  |  | c76172_g1_i1 | 0.75 | 0.06 | 0.34 | 0.38 |
| K01662 | 1-deoxy-D-xylulose-5-phosphate synthase | c91121_g1_i4 | 0.42 | 0.06 | 1.64 | 0.71 |
|  |  | c94588_g1_i1 | 0.42 | 0.55 | 1.32 | 0.76 |
|  |  | c94588_g1_i2 | 1.25 | 0.96 | 0.57 | 0.93 |
|  |  | c94588_g2_i1 | 1.56 | 1.6 | 0.89 | 1.35 |
|  |  | c99206_g1_i1 | 48.71 | 55.26 | 65.46 | 56.48 |
|  |  | c99206_g2_i2 | 73.18 | 93.4 | 104.26 | 90.28 |
|  |  | c99692_g1_i1 | 4.06 | 3.8 | 5.74 | 4.53 |
|  |  | c99692_g2_i1 | 2.57 | 5.72 | 5.41 | 4.57 |
|  |  | c99692_g3_i1 | 2.56 | 4.7 | 3.99 | 3.75 |
| K10960 | geranylgeranyl diphosphate | c102656_g1_i1 | 458.32 | 198.61 | 391.2 | 349.38 |
|  |  | c114241_g1_i1 | 46.99 | 17.47 | 31.93 | 32.13 |
|  |  | c163247_g1_i1 | 0.45 | 0.6 | 0.93 | 0.66 |
| K11778 | ditrans,polycis-polyprenyl diphosphate synthase | c79459_g2_i2 | 3.53 | 4.23 | 5.8 | 4.52 |
|  |  | c87272_g1_i1 | 260.79 | 215.13 | 241.66 | 239.19 |
|  |  | c98771_g2_i1 | 0.68 | 3.46 | 2.56 | 2.23 |
|  |  | c98771_g2_i2 | 2.3 | 2.79 | 1.69 | 2.26 |
|  |  | c98771_g3_i1 | 5.27 | 7.23 | 7.09 | 6.53 |
|  |  | c102940_g3_i2 | 1 | 1.06 | 0.38 | 0.81 |
|  |  | c102940_g3_i4 | 0.17 | 0.35 | 0.58 | 0.37 |
| K03527 | 4-hydroxy-3-methylbut-2-en-1-yl diphosphate reductase | c162685_g1_i1 | 98.37 | 202.8 | 212.66 | 171.2726 |
| K03526 | (E)-4-hydroxy-3-methylbut-2-enyl-diphosphate synthase | c84621_g1_i1 | 15.57 | 19.89 | 17.28 | 17.58 |
|  |  | c99530_g1_i2 | 51.18 | 42.26 | 53.82 | 49.09 |
|  |  | c2470_g1_i1 | 0.71 | 0.35 | 0.47 | 0.51 |
|  |  | c65280_g1_i1 | 2.52 | 1.62 | 1.83 | 1.99 |
| K00099 | 1-deoxy-D-xylulose-5-phosphate reductoisomerase | c114487_g1_i1 | 45.88 | 43.58 | 58.74 | 49.4 |
| K13789 | geranylgeranyl diphosphate synthase, type II | c91555_g1_i1 | 155.16 | 83.58 | 103.68 | 114.14 |
|  |  | c93806_g1_i1 | 0.67 | 0.61 | 6.35 | 2.54 |
|  |  | c93806_g1_i2 | 4.31 | 5.29 | 5.07 | 4.89 |
|  |  | c93806_g2_i1 | 6.61 | 4.28 | 1.77 | 4.22 |
|  |  | c93806_g3_i1 | 6.67 | 2.87 | 6.72 | 45.42 |
|  |  | c95153_g1_i1 | 0.86 | 3.62 | 3.95 | 2.81 |
|  |  | c31007_g1_i1 | 0.27 | 0.69 | 0.33 | 0.43 |
| K00021 | hydroxymethylglutaryl-CoA reductase (NADPH) | c73235_g1_i1 | 24.89 | 11.31 | 18.27 | 18.16 |
|  |  | c75709_g2_i1 | 0.46 | 1.29 | 0.2 | 0.65 |
|  |  | c79354_g1_i2 | 0.94 | 1.94 | 0.7 | 1.19 |
| K05356 | all-trans-nonaprenyl-diphosphate synthase | c97381_g2_i1 | 20.07 | 13.31 | 24.07 | 19.15 |
|  |  | c97381_g2_i2 | 10.55 | 11.46 | 11.57 | 11.119 |
|  |  | c97381_g2_i3 | 4.77 | 4.4 | 4.2 | 4.16 |
|  |  | c119324_g1_i1 | 1.09 | 1.58 | 1.19 | 1.29 |
| K00919 | 4-diphosphocytidyl-2-C-methyl-D-erythritol kinase | c86828_g1_i1 | 39.28 | 22.09 | 30.67 | 30.68 |
| K14066 | geranyl diphosphate synthase | c87669_g1_i1 | 12.01 | 13.07 | 11.46 | 12.18 |
| K06013 | STE24 endopeptidase | c53130_g1_i1 | 43.97 | 32.98 | 38.93 | 38.63 |
| K12506 | 2-C-methyl-D-erythritol 4-phosphate cytidylyltransfera | c77184_g1_i1 | 0.71 | 0.19 | 1.3 | 0.73 |
| K15892 | farnesol kinase | c92449_g1_i1 | 27.84 | 19.37 | 20.41 | 22.54 |
|  |  | c102701_g1_i1 | 1.54 | 1.86 | 1.9 | 1.77 |
|  |  | c102701_g1_i2 | 24.85 | 9.52 | 8.92 | 14.43 |
|  |  | c102701_g1_i3 | 2.94 | 3.93 | 3.33 | 3.4 |
|  |  | c102701_g1_i4 | 6.6 | 5.57 | 7.19 | 6.45 |
| K00991 | 2-C-methyl-D-erythritol 4-phosphate cytidylyltransferase | c129659_g1_i1 | 9.3 | 15.19 | 14.82 | 13.1 |
| K00787 | farnesyl diphosphate synthase | c80989_g2_i1 | 9.3 | 11.42 | 14.64 | 11.79 |
|  |  | c94812_g1_i1 | 9.67 | 14.49 | 3.34 | 9.17 |
|  |  | c94812_g2_i1 | 8 | 7.33 | 5.58 | 6.97 |
| K00869 | mevalonate kinase | c90032_g1_i1 | 2.43 | 0.95 | 0.85 | 1.41 |
|  |  | c90032_g1_i3 | 1.83 | 0.18 | 0.54 | 0.85 |
| K01770 | 2-C-methyl-D-erythritol 2,4-cyclodiphosphate synthase | c137532_g1_i1 | 143.73 | 80.1 | 152.7 | 125.51 |
| K01823 | isopentenyl-diphosphate Delta-isomerase | c91840_g1_i1 | 35.9 | 24.71 | 29.77 | 30.13 |
|  |  | c91840_g2_i1 | 14.46 | 13 | 17.85 | 15.1 |
|  |  | c137779_g1_i1 | 19.23 | 35.45 | 21.06 | 25.25 |
| K08658 | prenyl protein peptidase | c92764_g3_i1 | 2.82 | 4.59 | 3.99 | 3.8 |
| K15889 | prenylcysteine alpha-carboxyl methylesterase | c99522_g1_i1 | 4.74 | 4.45 | 3.91 | 4.37 |
| K01641 | hydroxymethylglutaryl-CoA synthase | c97793_g1_i1 | 34.17 | 24.66 | 25.32 | 28.05 |
|  |  | c97793_g2_i1 | 31.18 | 25.21 | 31.78 | 29.39 |
| K15891 | NAD+-dependent farnesol dehydrogenase | c85177_g1_i1 | 10.59 | 7.05 | 13.35 | 10.33 |
|  |  | c85177_g1_i2 | 1.62 | 2.1 | 0.46 | 1.39 |
| K00587 | protein-S-isoprenylcysteine O-methyltransferase | c65894_g1_i1 | 21.2 | 12.17 | 15.3 | 16.22 |
| K00938 | phosphomevalonate kinase | c101868_g1_i1 | 9.3 | 7.37 | 8.34 | 8.34 |
| K01597 | diphosphomevalonate decarboxylase | c58160_g1_i1 | 9.07 | 9.17 | 6.9 | 8.38 |
| K05906 | prenylcysteine oxidase | c99043_g1_i1 | 19.32 | 11.58 | 16.54 | 15.81 |
| K05954 | protein farnesyltransferase subunit beta | c87685_g1_i1 | 12.37 | 13.87 | 11.12 | 12.45 |
| K05955 | protein farnesyltransferase | c96923_g1_i1 | 11.3 | 9.37 | 12.43 | 11.03 |
| **Sesquiterpenoid and triterpenoid biosynthesis** | |  |  |  |  |  |
| K15803 | (-)-germacrene D synthase | c93107_g1_i3 | 2.12 | 1.03 | 2.09 | 1.75 |
|  |  | c103018_g1_i1 | 0.95 | 8.91 | 0.1 | 3.32 |
|  |  | c103113_g1_i1 | 11.8 | 7 | 1.88 | 6.89 |
| K14181 | valencene/7-epi-alpha-selinene synthase | c103113_g2_i3 | 8.38 | 8.87 | 2.03 | 6.43 |
| K00511 | squalene monooxygenase | c87389_g1_i1 | 2.36 | 0.58 | 1.71 | 1.55 |
|  |  | c87389_g2_i1 | 1.88 | 1.08 | 2.05 | 1.67 |
|  |  | c88756_g2_i1 | 4.99 | 6.26 | 1.36 | 4.2 |
|  |  | c99211_g2_i1 | 23.87 | 14.54 | 17.27 | 18.56 |
| K00801 | farnesyl-diphosphate farnesyltransferase | c151311_g1_i1 | 16.58 | 19.04 | 10.28 | 15.3 |
| K15813 | beta-amyrin synthase | c9113_g1_i1 | 0.9 | 1.31 | 0.98 | 1.06 |
|  |  | c53853_g1_i1 | 34.22 | 11.39 | 19.15 | 21.59 |
| K15891 | NAD+-dependent farnesol dehydrogenase | c85177_g1_i1 | 10.59 | 7.05 | 13.35 | 10.33 |
|  |  | c85177_g1_i2 | 1.62 | 2.1 | 0.46 | 1.39 |
| **Diterpenoid biosynthesis** | |  |  |  |  |  |
| K04125 | gibberellin 2-oxidase | c82387_g2_i1 | 5.16 | 0.61 | 1.15 | 2.31 |
|  |  | c83472_g1_i2 | 0.74 | 0.34 | 2.38 | 1.15 |
|  |  | c100346_g1_i1 | 1.33 | 1.3 | 4.88 | 2.5 |
|  |  | c1179_g1_i1 | 0.76 | 0.16 | 0.96 | 0.63 |
| K04120 | ent-copalyl diphosphate synthase | c101555_g2_i1 | 6.12 | 0.29 | 6.83 | 4.41 |
|  |  | c94452_g1_i2 | 3.63 | 6.06 | 2.04 | 3.91 |
|  |  | c94452_g1_i3 | 3.28 | 9.29 | 4.1 | 5.56 |
| K17982 | geranyllinalool synthase | c102617_g1_i1 | 0.21 | 1.57 | 1.32 | 1.03 |
|  |  | c102617_g1_i2 | 0.33 | 1.06 | 2.4 | 1.26 |
|  |  | c102617_g1_i3 | 5.34 | 8.51 | 10.19 | 8.01 |
|  |  | c102617_g1_i4 | 1.27 | 1.21 | 2.51 | 1.66 |
|  |  | c102617_g1_i5 | 0.23 | 0.48 | 0.13 | 0.28 |
| K04122 | ent-kaurene oxidase | c102312_g1_i1 | 2.22 | 4.32 | 7.32 | 4.62 |
|  |  | c103383_g1_i1 | 5.56 | 9.21 | 10.75 | 8.51 |
|  |  | c103383_g1_i6 | 1.3 | 1.79 | 3.44 | 2.18 |
| K04121 | ent-kaurene synthase | c103523_g1_i1 | 13.03 | 22.17 | 56.02 | 30.41 |
|  |  | c103523_g1_i3 | 4.43 | 15.48 | 7.07 | 8.99 |
| K04124 | gibberellin 3-beta-dioxygenase | c137564_g1_i1 | 44.09 | 2.38 | 18.76 | 21.74 |
| K04123 | ent-kaurenoic acid hydroxylase | c95795_g1_i1 | 0.59 | 1.27 | 1.1 | 0.99 |
| K05282 | gibberellin 20-oxidase | c98782_g1_i1 | 8.03 | 0.99 | 2.66 | 3.89 |
| **Monoterpenoid biosynthesis** | |  |  |  |  |  |
| K18108 | (-)-alpha-terpineol synthase | c101459_g2_i1 | 39.17 | 51.55 | 147.64 | 79.45 |
|  |  | c101459_g2_i2 | 390.24 | 71.83 | 341.44 | 267.84 |
|  |  | c163998_g1_i1 | 39.16 | 64.33 | 209.61 | 104.37 |
| K15086 | (3S)-linalool synthase | c89831_g1_i1 | 0.71 | 2.14 | 0.35 | 1.07 |
| K15095 | (+)-neomenthol dehydrogenase | c97726_g1_i1 | 44.05 | 27.15 | 43.65 | 38.28 |
|  |  | c97726_g1_i2 | 154.52 | 87.29 | 147.28 | 129.7 |
