# Supplementary material for: Transcriptome Analysis of Cinnamomum chago: A Revelation of Candidate Genes for Abiotic Stress Response and Terpenoid and Fatty Acid Biosyntheses
Source: Front Genet. 2018 Nov 5;9:505. doi: 10.3389/fgene.2018.00505 (PMC6231050; doi:10.3389/fgene.2018.00505)
Supplement: Supplementary file 10 [file Table_5.DOC]

***Supplementary Material***

**Characterization of the de novo *Cinnamomum chago* (Lauraceae) transcriptome reveals candidate genes for terpenoid, fatty acid biosyntheses and abiotic stress**

**Authors:** Xue Zhang, Shi-Kang Shen *,

***Address for Correspondence:** Shi-Kang Shen, School of Life Sciences, Yunnan University, No. 2 Green lake North road Kunming, Yunnan, 650091, the People’s Republic of China. Telephone:+86-871-65031412; Fax:+86-871-65031412;

**E-mail:** yunda123456@126.com

**Table S5** Candidate genes involved in Fatty acid biosynthesis pathway

| **KO ID** | **KEGG Annotation** | **Gene ID** | **Numbers of unineges** |
| --- | --- | --- | --- |
| **Fatty acid biosynthesis** | | | |
| K01897 | long-chain acyl-CoA synthetase | ACSL, fadD | 21 |
| K01961 | acetyl-CoA carboxylase, biotin carboxylase subunit | accC | 4 |
| K02160 | acetyl-CoA carboxylase biotin carboxyl carrier protein | accB, bccP | 3 |
| K03921 | acyl-[acyl-carrier-protein] desaturase | DESA1 | 4 |
| K11262 | acetyl-CoA carboxylase / biotin carboxylase 1 | ACACA | 1 |
| K01963 | acetyl-CoA carboxylase carboxyl transferase subunit beta | accD | 4 |
| K09458 | 3-oxoacyl-[acyl-carrier-protein] synthase II | fabF | 6 |
| K00645 | [acyl-carrier-protein] S-malonyltransferase | fabD | 1 |
| K00059 | 3-oxoacyl-[acyl-carrier protein] reductase | fabG | 6 |
| K00648 | 3-oxoacyl-[acyl-carrier-protein] synthase III | fabH | 5 |
| K01962 | acetyl-CoA carboxylase carboxyl transferase subunit alpha | accA | 2 |
| K02372 | 3-hydroxyacyl-[acyl-carrier-protein] dehydratase | fabZ | 1 |
| K10781 | fatty acyl-ACP thioesterase B | FATB | 3 |
| K00208 | enoyl-[acyl-carrier protein] reductase I | fabI | 3 |
| K10782 | fatty acyl-ACP thioesterase A | FATA | 1 |
| **Biosynthesis of unsaturated fatty acids** | | | |
| K10257 | acyl-lipid omega-3 desaturase | FAD8, desB | 4 |
| K00232 | acyl-CoA oxidase | ACOX1, ACOX3 | 6 |
| K03921 | acyl-[acyl-carrier-protein] desaturase | DESA1 | 4 |
| K00059 | 3-oxoacyl-[acyl-carrier protein] reductase | fabG | 6 |
| K10256 | omega-6 fatty acid desaturase / acyl-lipid omega-6 desaturase (Delta-12 desaturase) | FAD2 | 2 |
| K10251 | 17beta-estradiol 17-dehydrogenase / very-long-chain 3-oxoacyl-CoA reductase | HSD17B12, KAR, IFA38 | 4 |
| K07513 | acetyl-CoA acyltransferase 1 | ACAA1 | 2 |
| K10255 | acyl-lipid omega-6 desaturase (Delta-12 desaturase) | FAD6, desA | 1 |
| K10806 | acyl-CoA thioesterase YciA | yciA | 1 |
| K10258 | very-long-chain enoyl-CoA reductase | TER,TSC13, CER10 | 4 |
| K01068 | acyl-coenzyme A thioesterase 1/2/4 | ACOT1_2_4 | 2 |
| K10703 | very-long-chain (3R)-3-hydroxyacyl-CoA dehydratase | PHS1, PAS2 | 4 |
| **Linoleic acid metabolism** | | | |
| K14674 | TAG lipase / steryl ester hydrolase / phospholipase A2 / LPA acyltransferase | TGL4 | 4 |
| K00454 | lipoxygenase | LOX2S | 13 |
| K15718 | linoleate 9S-lipoxygenase | LOX1_5 | 3 |
| K01047 | secretory phospholipase A2 | PLA2G, SPLA2 | 1 |
| **alpha-Linolenic acid metabolism** | | | |
| K14674 | TAG lipase / steryl ester hydrolase / phospholipase A2 / LPA acyltransferase | TGL4 | 4 |
| K10526 | OPC-8:0 CoA ligase 1 | OPCL1 | 4 |
| K10527 | enoyl-CoA hydratase/3-hydroxyacyl-CoA dehydrogenase | MFP2 | 8 |
| K00454 | lipoxygenase | LOX2S | 13 |
| K10528 | hydroperoxide lyase | HPL | 2 |
| K00232 | acyl-CoA oxidase | ACOX1, ACOX3 | 6 |
| K05894 | 12-oxophytodienoic acid reductase | OPR | 5 |
| K01723 | hydroperoxide dehydratase | AOS | 3 |
| K18857 | alcohol dehydrogenase class-P | ADH1 | 3 |
| K00632 | acetyl-CoA acyltransferase | fadA, fadI | 2 |
| K07513 | acetyl-CoA acyltransferase 1 | ACAA1 | 2 |
| K10525 | allene oxide cyclase | AOC | 3 |
| K08241 | jasmonate O-methyltransferase | E2.1.1.141 | 3 |
| K01047 | secretory phospholipase A2 | PLA2G, SPLA2 | 1 |
| K10529 | alpha-dioxygenase | DOX1 | 1 |
