# Supplementary material for: Transcriptome Analysis of Cinnamomum chago: A Revelation of Candidate Genes for Abiotic Stress Response and Terpenoid and Fatty Acid Biosyntheses
Source: Front Genet. 2018 Nov 5;9:505. doi: 10.3389/fgene.2018.00505 (PMC6231050; doi:10.3389/fgene.2018.00505)
Supplement: Supplementary file 11 [file Table_6.DOC]

***Supplementary Material***

**Characterization of the de novo *Cinnamomum chago* (Lauraceae) transcriptome reveals candidate genes for terpenoid, fatty acid biosyntheses and abiotic stress**

**Authors:** Xue Zhang, Shi-Kang Shen *,

***Address for Correspondence:** Shi-Kang Shen, School of Life Sciences, Yunnan University, No. 2 Green lake North road Kunming, Yunnan, 650091, the People’s Republic of China. Telephone:+86-871-65031412; Fax:+86-871-65031412;

**E-mail:** yunda123456@126.com

**Table S6 The FPKM values of candidate genes involved in Fatty acid biosynthesis pathway**

| **KO ID** |  | **Unigene** | **Ccg1** | **Ccg2** | **Ccg3** | **Mean** |
| --- | --- | --- | --- | --- | --- | --- |
| **Fatty acid biosynthesis** | |  |  |  |  |  |
| K01897 | long-chain acyl-CoA synthetase | c82069_g1_i1 | 0.39 | 0.16 | 1.52 | 0.69 |
|  |  | c83099_g1_i1 | 16.83 | 35.64 | 32.65 | 28.37 |
|  |  | c83867_g1_i1 | 1.13 | 0.19 | 0.76 | 0.69 |
|  |  | c84893_g1_i1 | 0.51 | 0.25 | 0.98 | 0.58 |
|  |  | c86346_g2_i1 | 19.83 | 20.99 | 24.12 | 21.65 |
|  |  | c86346_g2_i2 | 5.09 | 3.52 | 2.57 | 3.73 |
|  |  | c87997_g1_i2 | 0.95 | 4.19 | 1.74 | 2.29 |
|  |  | c93761_g3_i1 | 0.95 | 1.71 | 1.33 | 1.33 |
|  |  | c93761_g3_i2 | 4.79 | 6.57 | 2.78 | 4.71 |
|  |  | c95936_g1_i1 | 2.8 | 10.49 | 8.25 | 7.18 |
|  |  | c96861_g4_i1 | 4.69 | 7.64 | 9.57 | 7.3 |
|  |  | c100744_g1_i1 | 0.86 | 3.26 | 0.66 | 1.59 |
|  |  | c100744_g1_i3 | 14.48 | 29.5 | 26.13 | 23.37 |
|  |  | c102547_g1_i6 | 24.18 | 20.73 | 23.79 | 22.9 |
|  |  | c102846_g1_i1 | 26.58 | 46.88 | 54.54 | 42.67 |
|  |  | c102846_g1_i3 | 0.08 | 1.26 | 0.72 | 0.69 |
|  |  | c102846_g1_i4 | 4.98 | 8.12 | 4.08 | 5.73 |
|  |  | c102846_g1_i5 | 2.49 | 3.89 | 2.11 | 2.83 |
|  |  | c102846_g1_i7 | 1.91 | 2.82 | 1.72 | 2.15 |
|  |  | c60667_g1_i1 | 3.98 | 5.33 | 10.82 | 6.71 |
|  |  | c60667_g1_i2 | 16.04 | 8.18 | 11.59 | 11.94 |
| K01961 | acetyl-CoA carboxylase, biotin carboxylase subunit | c97323_g1_i1 | 11.14 | 10.2 | 12.82 | 11.39 |
|  |  | c97323_g2_i1 | 3.23 | 8.97 | 3.54 | 5.25 |
|  |  | c100802_g1_i1 | 10.81 | 12.48 | 10.77 | 11.35 |
|  |  | c100802_g2_i1 | 13.89 | 13 | 30.82 | 19.24 |
| K02160 | acetyl-CoA carboxylase biotin carboxyl carrier protein | c101721_g3_i1 | 2.52 | 2.72 | 4.54 | 3.26 |
|  |  | c10462_g1_i1 | 0.5 | 0.31 | 1.95 | 0.92 |
|  |  | c163319_g1_i1 | 88.82 | 75.25 | 132.46 | 98.84 |
| K03921 | acyl-[acyl-carrier-protein] desaturase | c92029_g1_i1 | 17.36 | 18.9 | 22.63 | 19.63 |
|  |  | c92029_g2_i1 | 9.48 | 9.15 | 17.21 | 11.95 |
|  |  | c93836_g1_i1 | 226.94 | 137.59 | 202.43 | 188.99 |
|  |  | c102428_g2_i1 | 0.65 | 1.08 | 1.2 | 0.98 |
| K11262 | acetyl-CoA carboxylase / biotin carboxylase 1 | c102923_g1_i1 | 4.54 | 4.86 | 5.67 | 5.02 |
| K01963 | acetyl-CoA carboxylase carboxyl transferase subunit beta | c103313_g1_i1 | 2.65 | 3.91 | 2 | 2.85 |
|  |  | c103313_g1_i2 | 0.31 | 0.74 | 0.58 | 0.54 |
|  |  | c103313_g2_i1 | 3.26 | 3.28 | 2.96 | 3.17 |
|  |  | c140476_g1_i1 | 1.01 | 0.38 | 1.66 | 1.02 |
| K09458 | 3-oxoacyl-[acyl-carrier-protein] synthase II | c86000_g2_i2 | 27.01 | 45.09 | 39.46 | 37.19 |
|  |  | c90534_g2_i1 | 4.94 | 4.09 | 4.43 | 4.49 |
|  |  | c100278_g1_i2 | 10.65 | 8.52 | 10.11 | 9.76 |
|  |  | c100278_g1_i3 | 0.65 | 1.76 | 1.02 | 1.14 |
|  |  | c104280_g1_i1 | 56.33 | 32.81 | 51.86 | 47 |
|  |  | c57885_g2_i1 | 1.63 | 0.13 | 0.72 | 0.83 |
| K00645 | [acyl-carrier-protein] S-malonyltransferase | c98085_g1_i1 | 4.77 | 6 | 6.09 | 5.62 |
| K00059 | 3-oxoacyl-[acyl-carrier protein] reductase | c92148_g1_i1 | 4.8 | 3.4 | 7.92 | 5.37 |
|  |  | c93795_g1_i1 | 19.64 | 24.1 | 18.16 | 20.63 |
|  |  | c93795_g1_i2 | 9.53 | 6.01 | 6.84 | 7.46 |
|  |  | c96511_g1_i1 | 12.23 | 4.39 | 9.21 | 8.61 |
|  |  | c96964_g1_i1 | 113.63 | 53.32 | 117.75 | 94.9 |
|  |  | c96964_g1_i2 | 63.35 | 36.23 | 27.17 | 42.25 |
| K00648 | 3-oxoacyl-[acyl-carrier-protein] synthase III | c72169_g1_i1 | 15.77 | 12.95 | 21.2 | 16.64 |
|  |  | c80797_g1_i1 | 8.3 | 6.99 | 7.51 | 7.6 |
|  |  | c90057_g1_i1 | 0.92 | 0.37 | 1.27 | 0.85 |
|  |  | c90948_g1_i2 | 2.58 | 3 | 6.6 | 4.06 |
|  |  | c135596_g1_i1 | 5.64 | 0.81 | 7.36 | 4.6 |
| K01962 | acetyl-CoA carboxylase carboxyl transferase subunit alpha | c82274_g1_i1 | 7.69 | 29.9 | 20.36 | 19.32 |
|  |  | c114149_g1_i1 | 12.85 | 40.34 | 30.74 | 27.98 |
| K02372 | 3-hydroxyacyl-[acyl-carrier-protein] dehydratase | c96141_g3_i1 | 45.74 | 19.77 | 51.41 | 38.97 |
| K10781 | fatty acyl-ACP thioesterase B | c84309_g1_i1 | 0.54 | 1.53 | 0.15 | 0.74 |
|  |  | c85357_g1_i1 | 25.71 | 22.12 | 31.83 | 26.55 |
|  |  | c85357_g1_i2 | 9.16 | 10.04 | 12.02 | 10.41 |
| K00208 | enoyl-[acyl-carrier protein] reductase I | c78521_g1_i1 | 13.82 | 15.69 | 15.07 | 14.86 |
|  |  | c62916_g1_i1 | 1.4 | 2.6 | 6.1 | 3.37 |
|  |  | c63964_g1_i1 | 1.81 | 0.89 | 0.59 | 1.1 |
| K10782 |  | c92050_g4_i1 | 16.69 | 13.88 | 15.33 | 15.3 |
| **Biosynthesis of unsaturated fatty acids** | |  |  |  |  |  |
| K10257 | acyl-lipid omega-3 desaturase | c95001_g1_i1 | 0.56 | 0.32 | 1.45 | 0.78 |
|  |  | c101287_g1_i1 | 8.24 | 2.66 | 4.87 | 5.26 |
|  |  | c101287_g2_i1 | 7.17 | 2.64 | 7.07 | 5.63 |
|  |  | c101520_g1_i1 | 12.47 | 5.66 | 0.75 | 6.29 |
| K00232 | acyl-CoA oxidase | c95526_g1_i1 | 87.78 | 86.54 | 42.81 | 72.38 |
|  |  | c98898_g1_i1 | 26.05 | 26.12 | 28.28 | 26.82 |
|  |  | c98898_g1_i2 | 12.32 | 14.97 | 10.11 | 12.47 |
|  |  | c101348_g1_i2 | 17.54 | 32.33 | 23.74 | 24.54 |
|  |  | c115318_g1_i1 | 36.26 | 75.27 | 64.36 | 58.63 |
|  |  | c154360_g1_i1 | 1.91 | 1 | 0.35 | 1.09 |
| K03921 | acyl-[acyl-carrier-protein] desaturase | c92029_g1_i1 | 17.36 | 18.9 | 22.63 | 19.63 |
|  |  | c92029_g2_i1 | 9.48 | 9.15 | 17.21 | 11.95 |
|  |  | c93836_g1_i1 | 226.94 | 137.59 | 202.43 | 188.99 |
|  |  | c102428_g2_i1 | 0.65 | 1.08 | 1.2 | 0.98 |
| K00059 | 3-oxoacyl-[acyl-carrier protein] reductase | c92148_g1_i1 | 4.8 | 3.4 | 7.92 | 5.37 |
|  |  | c93795_g1_i1 | 19.64 | 24.1 | 18.16 | 20.63 |
|  |  | c93795_g1_i2 | 9.53 | 6.01 | 6.84 | 7.46 |
|  |  | c96511_g1_i1 | 12.23 | 4.39 | 9.21 | 8.61 |
|  |  | c96964_g1_i1 | 113.63 | 53.32 | 117.75 | 94.9 |
|  |  | c96964_g1_i2 | 63.35 | 36.23 | 27.17 | 42.25 |
| K10256 | omega-6 fatty acid desaturase / acyl-lipid omega-6 desaturase (Delta-12 desaturase) | c84497_g1_i1 | 18.75 | 22.12 | 26.66 | 22.51 |
|  |  | c85680_g2_i1 | 9.49 | 1.7 | 10.31 | 7.17 |
| K10251 | 17beta-estradiol 17-dehydrogenase / very-long-chain 3-oxoacyl-CoA reductase | c114059_g1_i1 | 65.16 | 44.55 | 24.88 | 44.86 |
|  |  | c167325_g1_i1 | 18.7 | 18.02 | 20.67 | 19.13 |
| K07513 | acetyl-CoA acyltransferase 1 | c97895_g1_i1 | 244.1 | 169.31 | 187.29 | 200.23 |
|  |  | c37467_g1_i1 | 60.88 | 49.7 | 49.05 | 53.21 |
| K10255 | acyl-lipid omega-6 desaturase (Delta-12 desaturase) | c57829_g1_i1 | 90.17 | 37.46 | 56.78 | 61.47 |
| K10806 | acyl-CoA thioesterase YciA | c74398_g2_i1 | 1.44 | 0.23 | 0.95 | 0.87 |
| K10258 | very-long-chain enoyl-CoA reductase | c93515_g1_i1 | 11.71 | 14.12 | 11.44 | 12.42 |
|  |  | c93515_g2_i1 | 8.89 | 7.79 | 4.64 | 7.11 |
|  |  | c165573_g1_i1 | 16.43 | 12.81 | 15.3 | 14.85 |
|  |  | c66527_g2_i1 | 88.59 | 65.33 | 76.62 | 76.85 |
| K01068 | acyl-coenzyme A thioesterase 1/2/4 | c86330_g1_i1 | 1.52 | 2.06 | 0.92 | 1.5 |
|  |  | c86330_g1_i2 | 6.68 | 4.97 | 5.92 | 5.86 |
| K10703 | very-long-chain (3R)-3-hydroxyacyl-CoA dehydratase | c93038_g1_i1 | 3.87 | 3.85 | 2.11 | 3.28 |
|  |  | c93038_g2_i1 | 4.76 | 2.28 | 0.4 | 2.48 |
|  |  | c98945_g1_i1 | 9.26 | 4.63 | 13.39 | 9.09 |
|  |  | c99110_g1_i1 | 9.26 | 11.39 | 3.52 | 8.06 |
| **Linoleic acid metabolism** | |  |  |  |  |  |
| K14674 | TAG lipase / steryl ester hydrolase / phospholipase A2 / LPA acyltransferase | c71694_g2_i1 | 2.55 | 1.83 | 4.23 | 2.87 |
|  |  | c99192_g1_i1 | 3.04 | 0.88 | 1.98 | 1.97 |
|  |  | c100670_g1_i1 | 14.23 | 12.59 | 14.39 | 13.74 |
|  |  | c65426_g1_i1 | 3.12 | 1.37 | 8.26 | 4.25 |
| K00454 | lipoxygenase | c69947_g1_i1 | 36.13 | 14.92 | 31.74 | 27.6 |
|  |  | c80506_g1_i1 | 4.93 | 8.92 | 13.57 | 9.14 |
|  |  | c80506_g1_i2 | 5.89 | 6.99 | 3.99 | 5.62 |
|  |  | c80506_g1_i3 | 4.72 | 4.15 | 8.46 | 5.78 |
|  |  | c83227_g1_i1 | 19.56 | 21.54 | 32.57 | 24.56 |
|  |  | c83227_g2_i1 | 35.92 | 18.41 | 57.84 | 37.39 |
|  |  | c94019_g1_i1 | 28.66 | 34.56 | 43.3 | 35.51 |
|  |  | c99000_g2_i1 | 200.73 | 261.48 | 144.13 | 202.11 |
|  |  | c100872_g1_i2 | 1.21 | 2.18 | 0.73 | 1.37 |
|  |  | c130155_g1_i1 | 0.37 | 0.37 | 0.25 | 0.33 |
|  |  | c138992_g1_i1 | 0.22 | 0.55 | 1.1 | 0.62 |
|  |  | c23899_g1_i1 | 79.35 | 57.56 | 103.97 | 80.29 |
|  |  | c23899_g2_i1 | 26.83 | 28.57 | 36.69 | 30.7 |
| K15718 | linoleate 9S-lipoxygenase | c92840_g2_i1 | 7.77 | 14.04 | 8.22 | 10.01 |
|  |  | c103270_g1_i1 | 45.29 | 30.78 | 50.34 | 42.14 |
|  |  | c61063_g1_i1 | 1.18 | 27.09 | 1.15 | 9.81 |
| K01047 | secretory phospholipase A2 | c91344_g1_i1 | 1.72 | 9.9 | 1.21 | 4.28 |
| **alpha-Linolenic acid metabolism** | |  |  |  |  |  |
| K14674 | TAG lipase / steryl ester hydrolase / phospholipase A2 / LPA acyltransferase | c71694_g2_i1 | 2.55 | 1.83 | 4.23 | 2.87 |
|  |  | c99192_g1_i1 | 3.04 | 0.88 | 1.98 | 1.97 |
|  |  | c100670_g1_i1 | 14.23 | 12.59 | 14.39 | 13.74 |
|  |  | c65426_g1_i1 | 3.12 | 1.37 | 8.26 | 4.25 |
| K10526 | OPC-8:0 CoA ligase 1 | c100772_g1_i1 | 3.75 | 1.97 | 3.94 | 3.22 |
|  |  | c100772_g1_i2 | 19.77 | 17.25 | 23.54 | 20.19 |
|  |  | c101989_g2_i2 | 9.8 | 4.94 | 2.84 | 5.86 |
|  |  | c61000_g2_i1 | 0.15 | 0.91 | 0.16 | 0.41 |
| K10527 | enoyl-CoA hydratase/3-hydroxyacyl-CoA dehydrogenase | c97453_g1_i1 | 2.58 | 0.25 | 0.67 | 1.17 |
|  |  | c97453_g1_i3 | 4.73 | 4.61 | 5.58 | 4.97 |
|  |  | c97453_g2_i2 | 8.66 | 3.65 | 5.09 | 5.8 |
|  |  | c100838_g1_i1 | 38.93 | 49.16 | 40.4 | 42.83 |
|  |  | c100838_g3_i1 | 0.16 | 0.35 | 0.83 | 0.45 |
|  |  | c100838_g3_i2 | 4.26 | 3.22 | 3.8 | 3.76 |
|  |  | c100838_g3_i2 | 4.26 | 3.22 | 3.8 | 3.76 |
|  |  | c163232_g1_i1 | 39.41 | 95.08 | 49.13 | 61.21 |
| K00454 | enoyl-CoA hydratase/3-hydroxyacyl-CoA dehydrogenase | c69947_g1_i1 | 36.13 | 14.92 | 31.74 | 27.6 |
|  |  | c80506_g1_i1 | 4.93 | 8.92 | 13.57 | 9.14 |
|  |  | c80506_g1_i2 | 5.89 | 6.99 | 3.99 | 5.62 |
|  |  | c80506_g1_i3 | 4.72 | 4.15 | 8.46 | 5.78 |
|  |  | c83227_g1_i1 | 19.56 | 21.54 | 32.57 | 24.56 |
|  |  | c83227_g2_i1 | 35.92 | 18.41 | 57.84 | 37.39 |
|  |  | c94019_g1_i1 | 28.66 | 34.56 | 43.3 | 35.51 |
|  |  | c99000_g2_i1 | 200.73 | 261.48 | 144.13 | 202.11 |
|  |  | c100872_g1_i2 | 1.21 | 2.18 | 0.73 | 1.37 |
|  |  | c130155_g1_i1 | 0.37 | 0.37 | 0.25 | 0.33 |
|  |  | c138992_g1_i1 | 0.22 | 0.55 | 1.1 | 0.62 |
|  |  | c23899_g1_i1 | 79.35 | 57.56 | 103.97 | 80.29 |
|  |  | c23899_g2_i1 | 26.83 | 28.57 | 36.69 | 30.7 |
| K10528 | hydroperoxide lyase | c84560_g1_i1 | 0.29 | 0.37 | 0.99 | 0.55 |
|  |  | c100898_g5_i1 | 54.15 | 42.85 | 64.92 | 53.97 |
| K00232 | acyl-CoA oxidase | c95526_g1_i1 | 87.78 | 86.54 | 42.81 | 72.38 |
|  |  | c98898_g1_i1 | 26.05 | 26.12 | 28.28 | 26.82 |
|  |  | c98898_g1_i2 | 12.32 | 14.97 | 10.11 | 12.47 |
|  |  | c101348_g1_i2 | 17.54 | 32.33 | 23.74 | 24.54 |
|  |  | c115318_g1_i1 | 36.26 | 75.27 | 64.36 | 58.63 |
|  |  | c154360_g1_i1 | 1.91 | 1 | 0.35 | 1.09 |
| K05894 | 12-oxophytodienoic acid reductase | c88642_g1_i1 | 22.46 | 17.1 | 18.57 | 19.38 |
|  |  | c99771_g2_i1 | 33.83 | 18.97 | 29.73 | 27.51 |
|  |  | c101693_g1_i1 | 4.61 | 2.18 | 3.44 | 3.41 |
|  |  | c60227_g1_i1 | 5.12 | 16.92 | 8.71 | 10.25 |
|  |  | c61078_g1_i1 | 8.45 | 8.13 | 18.77 | 11.78 |
| K01723 | hydroperoxide dehydratase | c68649_g1_i1 | 1.64 | 0.54 | 0.75 | 0.98 |
|  |  | c96988_g1_i2 | 0.86 | 1.85 | 0.52 | 1.08 |
|  |  | c103998_g1_i1 | 17.79 | 12.03 | 11.09 | 13.64 |
| K18857 | alcohol dehydrogenase class-P | c90501_g1_i1 | 38.56 | 26.88 | 17.04 | 27.49 |
|  |  | c90501_g1_i2 | 9.25 | 10.32 | 2.89 | 7.49 |
|  |  | c93073_g1_i2 | 1.76 | 0.26 | 1.5 | 1.17 |
| K00632 | acetyl-CoA acyltransferase | c76172_g1_i1 | 0.75 | 0.06 | 0.34 | 0.38 |
|  |  | c84206_g1_i3 | 0.91 | 0.15 | 0 | 0.35 |
| K07513 | acetyl-CoA acyltransferase 1 | c97895_g1_i1 | 244.1 | 169.31 | 187.29 | 200.23 |
|  |  | c37467_g1_i1 | 60.88 | 49.7 | 49.05 | 53.21 |
| K10525 | allene oxide cyclase | c96909_g1_i1 | 84.6 | 62.27 | 88.68 | 78.52 |
|  |  | c96909_g1_i2 | 8.89 | 3.37 | 11.67 | 7.98 |
|  |  | c96909_g2_i1 | 2.47 | 3.86 | 14.67 | 7 |
| K08241 | jasmonate O-methyltransferase | c82939_g1_i1 | 0.76 | 1.78 | 0.75 | 1.1 |
|  |  | c89074_g1_i1 | 1.03 | 3.22 | 1.5 | 1.92 |
|  |  | c89153_g1_i1 | 1.5 | 3.24 | 0.68 | 1.81 |
| K01047 | secretory phospholipase A2 | c91344_g1_i1 | 1.72 | 9.9 | 1.21 | 4.28 |
| K10529 | alpha-dioxygenase | c99966_g3_i4 | 0.22 | 1.01 | 0.28 | 0.5 |
