# Supplementary material for: Transcriptome Analysis of Cinnamomum chago: A Revelation of Candidate Genes for Abiotic Stress Response and Terpenoid and Fatty Acid Biosyntheses
Source: Front Genet. 2018 Nov 5;9:505. doi: 10.3389/fgene.2018.00505 (PMC6231050; doi:10.3389/fgene.2018.00505)
Supplement: Supplementary file 12 [file Table_7.DOC]

***Supplementary Material***

**Characterization of the de novo *Cinnamomum chago* (Lauraceae) transcriptome reveals candidate genes for terpenoid, fatty acid biosyntheses and abiotic stress**

**Authors:** Xue Zhang, Shi-Kang Shen *,

***Address for Correspondence:** Shi-Kang Shen, School of Life Sciences, Yunnan University, No. 2 Green lake North road Kunming, Yunnan, 650091, the People’s Republic of China. Telephone:+86-871-65031412; Fax:+86-871-65031412;

**E-mail:** yunda123456@126.com

**Table S7 Candidate genes related stress to water deprivation in *C. chago*** transcriptome

| **KO ID** | **Gene** | **KEGG Annotation** | **Numbers of unineges** |
| --- | --- | --- | --- |
| K00799 | GST, gst | glutathione S-transferase | 28 |
| K14638 | SLC15A3_4, PHT | solute carrier family 15 (peptide/histidine transporter), member 3/4 | 34 |
| K01535 | E3.6.3.6 | H+-transporting ATPase | 23 |
| K10999 | CESA | cellulose synthase A | 20 |
| K00128 | E1.2.1.3 | aldehyde dehydrogenase (NAD+) | 12 |
| K09286 | EREBP | EREBP-like factor | 32 |
| K01115 | PLD1_2 | phospholipase D1/2 | 17 |
| K01188 | E3.2.1.21 | beta-glucosidase | 18 |
| K08235 | E2.4.1.207 | xyloglucan:xyloglucosyl transferase | 12 |
| K09872 | PIP | aquaporin PIP | 17 |
| K00695 | E2.4.1.13 | sucrose synthase（SUS） | 9 |
| K09487 | HSP90B, TRA1 | heat shock protein 90kDa beta | 12 |
| K16280 | RGLG | E3 ubiquitin-protein ligase RGLG | 13 |
| K14498 | SNRK2 | serine/threonine-protein kinase SRK2 | 12 |
| K00688 | E2.4.1.1, glgP, PYG | glycogen phosphorylase | 5 |
| K00432 | E1.11.1.9 | glutathione peroxidase | 7 |
| K17279 | REEP5_6 | receptor expression-enhancing protein 5/6 | 9 |
| K10523 | SPOP | speckle-type POZ protein | 13 |
| K01177 | E3.2.1.2 | beta-amylase | 8 |
| K17095 | ANXA7_11 | annexin A7/11 | 8 |
| K14803 | PTC2_3 | protein phosphatase PTC2/3 | 10 |
| K09250 | CNBP | cellular nucleic acid-binding protein | 8 |
| K16911 | DDX21 | ATP-dependent RNA helicase DDX21 | 3 |
| K09840 | NCED | 9-cis-epoxycarotenoid dioxygenase | 6 |
| K16277 | DRIP | E3 ubiquitin-protein ligase DRIP | 6 |
| K11254 | H4 | histone H4 | 4 |
| K13422 | MYC2 | transcription factor MYC2 | 8 |
| K00511 | SQLE, ERG1 | squalene monooxygenase | 4 |
| K17679 | MSS116 | ATP-dependent RNA helicase MSS116, mitochondrial | 3 |
| K06268 | PPP3R, CNB | serine/threonine-protein phosphatase 2B regulatory subunit | 5 |
| K12885 | RBMX, HNRNPG | heterogeneous nuclear ribonucleoprotein G | 4 |
| K08232 | E1.6.5.4 | monodehydroascorbate reductase (NADH) | 4 |
| K03627 | MBF1 | putative transcription factor | 4 |
| K11713 | PGTB1 | geranylgeranyl transferase type-1 subunit beta | 3 |
| K17991 | PXG | peroxygenase | 2 |
| K06634 | CCNH | cyclin H | 1 |
| K12118 | CRY1 | cryptochrome 1 | 1 |
| K05955 | FNTA | protein farnesyltransferase/geranylgeranyltransferase type-1 subunit alpha | 1 |
| K05954 | FNTB | protein farnesyltransferase subunit beta | 1 |
| All |  | 40 | 387 |
|  |  |  |  |
