# Supplementary material for: Transcriptome Analysis of Cinnamomum chago: A Revelation of Candidate Genes for Abiotic Stress Response and Terpenoid and Fatty Acid Biosyntheses
Source: Front Genet. 2018 Nov 5;9:505. doi: 10.3389/fgene.2018.00505 (PMC6231050; doi:10.3389/fgene.2018.00505)
Supplement: Supplementary file 13 [file Table_8.DOC]

***Supplementary Material***

**Characterization of the de novo *Cinnamomum chago* (Lauraceae) transcriptome reveals candidate genes for terpenoid, fatty acid biosyntheses and abiotic stress**

**Authors:** Xue Zhang, Shi-Kang Shen *,

***Address for Correspondence:** Shi-Kang Shen, School of Life Sciences, Yunnan University, No. 2 Green lake North road Kunming, Yunnan, 650091, the People’s Republic of China. Telephone:+86-871-65031412; Fax:+86-871-65031412;

**E-mail:** yunda123456@126.com

**Table S8 The FPKM values of candidate genes related stress to water deprivation in *C. chago* transcriptome**

| KO ID | Gene ID | KEGG Annotation | Unigene | Ccg1 | Ccg2 | Ccg3 |
| --- | --- | --- | --- | --- | --- | --- |
| K00799 | GST, gst | glutathione S-transferase | c70826_g1_i1 | 0.52 | 1.25 | 0.29 |
|  |  |  | c71073_g1_i1 | 12.88 | 0.88 | 0.59 |
|  |  |  | c73020_g1_i1 | 72.03 | 97.69 | 72.66 |
|  |  |  | c75769_g1_i1 | 92.03 | 115.08 | 42.07 |
|  |  |  | c75886_g1_i1 | 22.72 | 29.39 | 12.13 |
|  |  |  | c75886_g1_i2 | 4.79 | 8.52 | 15.14 |
|  |  |  | c77125_g1_i1 | 131.03 | 79.81 | 97.83 |
|  |  |  | c84041_g1_i1 | 801.76 | 247.15 | 448.7 |
|  |  |  | c89710_g2_i1 | 5.16 | 4.68 | 4.79 |
|  |  |  | c92981_g1_i1 | 37.8 | 7.23 | 4.58 |
|  |  |  | c92981_g2_i1 | 6.49 | 3.25 | 0.25 |
|  |  |  | c94020_g2_i1 | 15.3 | 0.79 | 2.38 |
|  |  |  | c95620_g1_i1 | 10.7 | 3.96 | 3.88 |
|  |  |  | c99082_g1_i1 | 588.11 | 233.58 | 205.5 |
|  |  |  | c99342_g1_i1 | 1.59 | 3.87 | 5.01 |
|  |  |  | c100118_g1_i1 | 861.29 | 514.4 | 822.88 |
|  |  |  | c100278_g1_i1 | 3.72 | 4.77 | 4.35 |
|  |  |  | c100278_g2_i1 | 14.28 | 11.5 | 5.61 |
|  |  |  | c100487_g3_i1 | 108.45 | 115.79 | 86.12 |
|  |  |  | c101386_g1_i10 | 0.55 | 0.27 | 0.4 |
|  |  |  | c101990_g1_i1 | 62.32 | 65.14 | 105.19 |
|  |  |  | c101990_g1_i2 | 15.98 | 5.59 | 34.64 |
|  |  |  | c102275_g1_i1 | 88.24 | 56.78 | 18.82 |
|  |  |  | c102275_g1_i2 | 350.13 | 271.29 | 76.58 |
|  |  |  | c127769_g1_i1 | 125.51 | 62.71 | 67.6 |
|  |  |  | c39637_g1_i1 | 0.72 | 0.28 | 0.39 |
|  |  |  | c42301_g2_i1 | 3.23 | 2.24 | 17.72 |
|  |  |  | c66827_g1_i1 | 627.17 | 264.65 | 416.11 |
| K14638 | SLC15A3_4, PHT | solute carrier family 15 (peptide/histidine transporter), member 3/4 | c69689_g1_i1 | 1.43 | 1.59 | 0.17 |
|  |  |  | c69689_g2_i1 | 1.45 | 1.35 | 2.09 |
|  |  |  | c70946_g1_i1 | 9.16 | 9.99 | 10.82 |
|  |  |  | c72182_g2_i1 | 0.43 | 1.17 | 0.52 |
|  |  |  | c76363_g2_i1 | 1.33 | 0.77 | 0.61 |
|  |  |  | c79635_g2_i1 | 25.76 | 17.12 | 15.78 |
|  |  |  | c86003_g1_i1 | 5.51 | 5.95 | 2.88 |
|  |  |  | c86830_g1_i1 | 0 | 0.87 | 0.36 |
|  |  |  | c89551_g1_i1 | 0.92 | 0.34 | 2.84 |
|  |  |  | c89985_g1_i1 | 4.33 | 15.29 | 7.49 |
|  |  |  | c95832_g1_i1 | 8.01 | 22.15 | 16.55 |
|  |  |  | c95844_g1_i2 | 4.68 | 2.53 | 5.83 |
|  |  |  | c96793_g1_i1 | 2.07 | 3.92 | 3.38 |
|  |  |  | c96793_g2_i1 | 1.44 | 1.73 | 2.41 |
|  |  |  | c96826_g1_i1 | 23.18 | 25.83 | 30.39 |
|  |  |  | c97297_g1_i1 | 44.32 | 40.24 | 54.38 |
|  |  |  | c97437_g1_i1 | 27.47 | 42.42 | 40.63 |
|  |  |  | c98092_g2_i1 | 12.32 | 4.95 | 1.22 |
|  |  |  | c99184_g3_i1 | 1.76 | 0.61 | 0.63 |
|  |  |  | c100545_g1_i1 | 51.16 | 14.18 | 26.77 |
|  |  |  | c100545_g2_i2 | 7.52 | 4.9 | 5.26 |
|  |  |  | c100954_g3_i2 | 0.35 | 3.32 | 0.19 |
|  |  |  | c101285_g2_i1 | 60.17 | 60.24 | 61.26 |
|  |  |  | c10163_g1_i1 | 6.52 | 0.54 | 0.92 |
|  |  |  | c101711_g2_i1 | 0.64 | 1.18 | 0.35 |
|  |  |  | c102873_g2_i1 | 0.52 | 1.42 | 0.41 |
|  |  |  | c103032_g1_i1 | 2.08 | 3.19 | 1.48 |
|  |  |  | c103032_g2_i1 | 2.23 | 2.29 | 9.67 |
|  |  |  | c103032_g3_i1 | 3.61 | 2.44 | 13.94 |
|  |  |  | c103032_g4_i1 | 4.13 | 3.02 | 3.84 |
|  |  |  | c104540_g1_i1 | 1.54 | 0.98 | 0.95 |
|  |  |  | c106538_g1_i1 | 2.01 | 1.23 | 0.09 |
|  |  |  | c142435_g1_i1 | 5.28 | 3.46 | 1.38 |
|  |  |  | c157211_g1_i1 | 5.06 | 1.41 | 0.33 |
| K01535 | E3.6.3.6 | H+-transporting ATPase | c76833_g1_i1 | 3.31 | 4.6 | 14.55 |
|  |  |  | c76833_g2_i1 | 17.61 | 13.81 | 19.94 |
|  |  |  | c76833_g3_i1 | 11.45 | 9.52 | 15.26 |
|  |  |  | c84162_g1_i1 | 22.99 | 31.17 | 31.01 |
|  |  |  | c84162_g3_i1 | 44.7 | 62.52 | 57.49 |
|  |  |  | c84162_g4_i1 | 25.01 | 37.73 | 42.65 |
|  |  |  | c84261_g1_i1 | 0.33 | 0.4 | 0.41 |
|  |  |  | c84984_g1_i1 | 1.55 | 0.21 | 3.4 |
|  |  |  | c84984_g2_i1 | 1.46 | 0.87 | 0.57 |
|  |  |  | c92106_g1_i1 | 25.2 | 25.03 | 40.72 |
|  |  |  | c94423_g1_i1 | 0.59 | 2.67 | 3.09 |
|  |  |  | c94423_g2_i1 | 2.68 | 1.65 | 1.31 |
|  |  |  | c94606_g1_i1 | 58.2 | 78.22 | 82.27 |
|  |  |  | c98853_g1_i2 | 0.55 | 1.38 | 0.41 |
|  |  |  | c98853_g2_i1 | 0.67 | 0.88 | 0.5 |
|  |  |  | c98853_g4_i2 | 0.54 | 1.01 | 1.05 |
|  |  |  | c98853_g7_i1 | 27.72 | 21.41 | 19.28 |
|  |  |  | c101570_g3_i2 | 0.95 | 2.8 | 0.56 |
|  |  |  | c102202_g1_i1 | 15.32 | 5.12 | 8.28 |
|  |  |  | c105199_g1_i1 | 25.3 | 74.12 | 225.17 |
|  |  |  | c120312_g1_i1 | 38.79 | 49.4 | 54.25 |
|  |  |  | c164405_g1_i1 | 0.86 | 1.22 | 1.36 |
|  |  |  | c63049_g1_i1 | 2.1 | 1.18 | 0.65 |
| K10999 | CESA | cellulose synthase A | c69326_g1_i1 | 2.53 | 3.65 | 3.16 |
|  |  |  | c75970_g1_i1 | 4.55 | 22.24 | 6.5 |
|  |  |  | c83057_g1_i1 | 1.34 | 3.38 | 5.13 |
|  |  |  | c84391_g1_i1 | 1.45 | 0.54 | 1.58 |
|  |  |  | c94968_g1_i1 | 0.2 | 1.35 | 0.7 |
|  |  |  | c95663_g2_i1 | 0.43 | 2.86 | 2.3 |
|  |  |  | c96671_g2_i1 | 0.71 | 26.11 | 0.84 |
|  |  |  | c96769_g1_i1 | 5.02 | 4.49 | 5.59 |
|  |  |  | c99478_g2_i1 | 2.66 | 5.94 | 2.54 |
|  |  |  | c99478_g3_i1 | 9.78 | 15.42 | 4.45 |
|  |  |  | c99478_g7_i1 | 10.21 | 16.6 | 10.43 |
|  |  |  | c99478_g8_i1 | 19.87 | 31.58 | 7.2 |
|  |  |  | c99478_g9_i1 | 1.89 | 7.45 | 8.08 |
|  |  |  | c102108_g3_i1 | 6.04 | 13.82 | 3.55 |
|  |  |  | c102333_g6_i3 | 18.24 | 31.72 | 23.98 |
|  |  |  | c102333_g7_i1 | 27.2 | 42.16 | 36.68 |
|  |  |  | c102333_g8_i1 | 0.37 | 48.93 | 0.08 |
|  |  |  | c102737_g1_i1 | 43.3 | 53.71 | 42.16 |
|  |  |  | c141123_g1_i1 | 1.28 | 1.92 | 0.46 |
|  |  |  | c54865_g1_i1 | 0.4 | 21.96 | 0.11 |
| K00128 | E1.2.1.3 | aldehyde dehydrogenase (NAD+) | c73024_g1_i1 | 79.32 | 36.53 | 36.59 |
|  |  |  | c78346_g1_i1 | 1.34 | 0.85 | 0.47 |
|  |  |  | c83962_g1_i1 | 0.21 | 1.72 | 0.47 |
|  |  |  | c83962_g2_i1 | 0.62 | 2.16 | 0.41 |
|  |  |  | c88887_g1_i1 | 1.19 | 0.61 | 3.46 |
|  |  |  | c93880_g1_i1 | 58.13 | 101.56 | 130.66 |
|  |  |  | c97249_g1_i1 | 1.54 | 6.86 | 0.71 |
|  |  |  | c102456_g1_i1 | 66 | 38.85 | 46.79 |
|  |  |  | c102456_g2_i1 | 108.41 | 122.75 | 150.77 |
|  |  |  | c109362_g1_i1 | 0.72 | 0.29 | 0.79 |
|  |  |  | c137558_g1_i1 | 37.77 | 35.87 | 44.78 |
|  |  |  | c32267_g1_i1 | 0.16 | 1.73 | 1.8 |
| K09286 | EREBP | EREBP-like factor | c68179_g1_i1 | 0.96 | 0.54 | 24.51 |
|  |  |  | c69316_g2_i1 | 66.8 | 52.81 | 106.76 |
|  |  |  | c74214_g1_i1 | 24.94 | 16.02 | 30.34 |
|  |  |  | c74214_g2_i1 | 23.18 | 17.49 | 26.46 |
|  |  |  | c77451_g1_i1 | 43.51 | 44.61 | 51.62 |
|  |  |  | c78299_g2_i1 | 1.58 | 16.8 | 53.3 |
|  |  |  | c81390_g1_i1 | 1.32 | 0.74 | 3.33 |
|  |  |  | c84324_g1_i1 | 3.94 | 12.21 | 12.85 |
|  |  |  | c84367_g1_i1 | 5.56 | 7.41 | 26.24 |
|  |  |  | c84627_g1_i1 | 15.21 | 15.85 | 19.35 |
|  |  |  | c88114_g2_i1 | 6.86 | 11.67 | 9.95 |
|  |  |  | c92630_g1_i1 | 134.13 | 131.51 | 131.33 |
|  |  |  | c92784_g1_i1 | 5.75 | 4.23 | 5.76 |
|  |  |  | c92806_g1_i1 | 11.89 | 8.32 | 61.76 |
|  |  |  | c94758_g1_i1 | 24.05 | 13.41 | 30.88 |
|  |  |  | c94862_g2_i1 | 1.63 | 1.23 | 15.65 |
|  |  |  | c96312_g4_i1 | 61.67 | 62.64 | 167.14 |
|  |  |  | c98828_g1_i1 | 170.75 | 306.68 | 406.98 |
|  |  |  | c98939_g1_i2 | 83.25 | 149.62 | 112.27 |
|  |  |  | c100057_g1_i1 | 268.74 | 367.18 | 566.12 |
|  |  |  | c100057_g1_i2 | 22.31 | 27.11 | 113.21 |
|  |  |  | c100057_g2_i1 | 42.2 | 47.59 | 210.91 |
|  |  |  | c119107_g1_i1 | 3.65 | 0.37 | 0.75 |
|  |  |  | c137498_g1_i1 | 206.65 | 202.16 | 234.35 |
|  |  |  | c137676_g1_i1 | 27.83 | 16.63 | 14.85 |
|  |  |  | c142150_g1_i1 | 1.52 | 15.3 | 1.84 |
|  |  |  | c146383_g1_i1 | 0.53 | 1.02 | 1.38 |
|  |  |  | c150972_g1_i1 | 44.39 | 34.22 | 46.67 |
|  |  |  | c16438_g1_i1 | 34.26 | 30.94 | 45.28 |
|  |  |  | c59312_g1_i1 | 2.43 | 9.57 | 35.03 |
|  |  |  | c59312_g2_i1 | 1.64 | 3.04 | 20.4 |
|  |  |  | c80244_g1_i1 | 118.52 | 96.54 | 139.16 |
| K01115 | PLD1_2 | phospholipase D1/2 | c74421_g1_i1 | 33.5 | 15.5 | 21.02 |
|  |  |  | c74421_g3_i1 | 18.88 | 9.65 | 12.15 |
|  |  |  | c74421_g4_i1 | 39.44 | 15.1 | 32.76 |
|  |  |  | c77873_g1_i1 | 17.11 | 38.24 | 32.14 |
|  |  |  | c79535_g2_i1 | 3.41 | 0.84 | 0.58 |
|  |  |  | c79535_g3_i1 | 0.1 | 1.04 | 0.49 |
|  |  |  | c80516_g1_i1 | 1.17 | 0.74 | 0.9 |
|  |  |  | c85785_g1_i1 | 42.02 | 65.4 | 49.43 |
|  |  |  | c88850_g2_i1 | 1.18 | 2.89 | 1.97 |
|  |  |  | c88850_g3_i1 | 0.26 | 0.48 | 0.15 |
|  |  |  | c88850_g3_i2 | 3.09 | 2.37 | 0.41 |
|  |  |  | c88850_g3_i3 | 0.73 | 2.02 | 2.37 |
|  |  |  | c91594_g1_i1 | 17.42 | 20.03 | 19.12 |
|  |  |  | c102527_g2_i1 | 2.2 | 4.92 | 3.58 |
|  |  |  | c102527_g2_i3 | 0.12 | 1.25 | 0.65 |
|  |  |  | c102527_g2_i4 | 2.36 | 2.12 | 1.82 |
|  |  |  | c102527_g3_i2 | 1.79 | 0.91 | 1.28 |
|  |  |  | c102937_g1_i1 | 35.28 | 11.65 | 10.57 |
| K01188 | E3.2.1.21 | beta-glucosidase | c78728_g3_i1 | 0.14 | 0.78 | 0.47 |
|  |  |  | c87858_g1_i3 | 0.47 | 1.38 | 1.25 |
|  |  |  | c93354_g1_i1 | 12.37 | 8.46 | 10.37 |
|  |  |  | c93742_g1_i1 | 0.18 | 0.47 | 0.71 |
|  |  |  | c97127_g2_i8 | 3.8 | 2.7 | 3.58 |
|  |  |  | c97285_g1_i1 | 0.82 | 0.92 | 0.77 |
|  |  |  | c99799_g1_i1 | 1.96 | 3.05 | 2.04 |
|  |  |  | c99799_g1_i3 | 0.26 | 1.19 | 0.09 |
|  |  |  | c99799_g2_i3 | 0.74 | 1.47 | 1.39 |
|  |  |  | c99799_g2_i4 | 0.31 | 0.95 | 0.72 |
|  |  |  | c100350_g1_i4 | 4.14 | 2.58 | 2.07 |
|  |  |  | c100538_g1_i1 | 30.81 | 23.37 | 91.89 |
|  |  |  | c100546_g1_i2 | 2.38 | 3.54 | 3.94 |
|  |  |  | c100546_g2_i1 | 10.51 | 20.34 | 15.75 |
|  |  |  | c66570_g1_i1 | 0.34 | 1.85 | 1.85 |
|  |  |  | c78728_g2_i1 | 0.66 | 0.87 | 0.31 |
|  |  |  | c95622_g1_i2 | 0.3 | 0.81 | 1.77 |
|  |  |  | c95622_g1_i4 | 1.25 | 0.76 | 0.35 |
| K08235 | E2.4.1.207 | xyloglucan:xyloglucosyl transferase | c81385_g1_i1 | 0.65 | 8.84 | 0.36 |
|  |  |  | c88422_g1_i1 | 0.28 | 1.6 | 0.37 |
|  |  |  | c90497_g1_i1 | 1.25 | 23.37 | 9.7 |
|  |  |  | c97850_g1_i1 | 4.34 | 115.99 | 5.62 |
|  |  |  | c97850_g1_i2 | 5.92 | 69.38 | 3.95 |
|  |  |  | c99314_g2_i1 | 5.3 | 7.83 | 1.09 |
|  |  |  | c101319_g6_i1 | 1.43 | 1.4 | 2.08 |
|  |  |  | c101319_g6_i4 | 1.84 | 3.82 | 1.41 |
|  |  |  | c29268_g1_i1 | 0.72 | 0.26 | 1.6 |
|  |  |  | c95270_g2_i1 | 3.37 | 3.47 | 0.52 |
|  |  |  | c96404_g3_i1 | 2.78 | 1.94 | 3.05 |
|  |  |  | c101530_g1_i3 | 8.63 | 13.82 | 32.72 |
| K09872 | PIP | aquaporin PIP | c88391_g2_i1 | 3.53 | 1.08 | 2.68 |
|  |  |  | c89389_g2_i1 | 438.97 | 367.73 | 385.48 |
|  |  |  | c89389_g2_i2 | 366.05 | 298.67 | 346.33 |
|  |  |  | c100688_g1_i1 | 2.89 | 8.27 | 2.01 |
|  |  |  | c100688_g1_i2 | 3.35 | 8.47 | 1.38 |
|  |  |  | c125607_g1_i1 | 1157.12 | 688.79 | 747.37 |
|  |  |  | c129209_g1_i1 | 0.57 | 0.43 | 0.62 |
|  |  |  | c149103_g1_i1 | 1.16 | 0.43 | 1.26 |
|  |  |  | c80764_g2_i1 | 5.79 | 20.77 | 2.61 |
|  |  |  | c82601_g1_i1 | 8.47 | 10.51 | 3.94 |
|  |  |  | c82601_g2_i1 | 4.3 | 19.49 | 4.48 |
|  |  |  | c86943_g1_i1 | 0.09 | 1.15 | 0.03 |
|  |  |  | c93824_g1_i1 | 78.42 | 132.33 | 107.29 |
|  |  |  | c100564_g2_i1 | 17.87 | 31.51 | 10.33 |
|  |  |  | c100564_g3_i1 | 73.77 | 161.54 | 142.25 |
|  |  |  | c13747_g1_i2 | 9.89 | 17 | 24.36 |
|  |  |  | c46136_g2_i1 | 17.82 | 13.1 | 26.99 |
| K00695 | E2.4.1.13 | sucrose synthase | c80764_g2_i1 | 5.79 | 20.77 | 2.61 |
|  |  |  | c82601_g1_i1 | 8.47 | 10.51 | 3.94 |
|  |  |  | c82601_g2_i1 | 4.3 | 19.49 | 4.48 |
|  |  |  | c86943_g1_i1 | 0.09 | 1.15 | 0.03 |
|  |  |  | c93824_g1_i1 | 78.42 | 132.33 | 107.29 |
|  |  |  | c100564_g2_i1 | 17.87 | 31.51 | 10.33 |
|  |  |  | c100564_g3_i1 | 73.77 | 161.54 | 142.25 |
|  |  |  | c13747_g1_i2 | 9.89 | 17 | 24.36 |
|  |  |  | c46136_g2_i1 | 17.82 | 13.1 | 26.99 |
| K09487 | HSP90B, TRA1 | heat shock protein 90kDa beta | c87175_g1_i1 | 1.74 | 1.12 | 1.01 |
|  |  |  | c87175_g2_i1 | 2.31 | 0.87 | 1.26 |
|  |  |  | c87589_g1_i1 | 20.95 | 46.03 | 63.05 |
|  |  |  | c88380_g1_i1 | 3.08 | 1.99 | 1 |
|  |  |  | c88380_g1_i2 | 1.74 | 0.55 | 0.32 |
|  |  |  | c100349_g1_i1 | 10.08 | 34.57 | 16.99 |
|  |  |  | c100349_g1_i2 | 23.68 | 44.03 | 36.33 |
|  |  |  | c100349_g1_i3 | 12.08 | 41.17 | 20.3 |
|  |  |  | c152123_g1_i1 | 10.01 | 16.45 | 15.53 |
|  |  |  | c153841_g1_i1 | 16.92 | 22.96 | 24.25 |
|  |  |  | c53211_g1_i1 | 23.19 | 17.5 | 21.25 |
|  |  |  | c53211_g2_i1 | 15.74 | 12.6 | 14.83 |
| K16280 | RGLG | E3 ubiquitin-protein ligase RGLG | c85057_g1_i1 | 0.4 | 0.59 | 1.65 |
|  |  |  | c90838_g2_i1 | 3.96 | 6.94 | 5.37 |
|  |  |  | c90838_g2_i2 | 0.53 | 1.25 | 0.17 |
|  |  |  | c92455_g1_i1 | 65.37 | 55.8 | 38.2 |
|  |  |  | c94908_g1_i1 | 8.82 | 8.51 | 8.5 |
|  |  |  | c94908_g2_i1 | 16.95 | 15.63 | 14.06 |
|  |  |  | c94908_g2_i2 | 1.22 | 2.19 | 4.51 |
|  |  |  | c94908_g3_i1 | 12.95 | 12.21 | 20.57 |
|  |  |  | c99790_g1_i1 | 52.43 | 35.4 | 45.87 |
|  |  |  | c100653_g1_i2 | 0.93 | 1.17 | 0.31 |
|  |  |  | c100653_g1_i3 | 0.91 | 1 | 1.12 |
|  |  |  | c100653_g1_i4 | 2.44 | 2.43 | 1.36 |
|  |  |  | c101332_g5_i1 | 2.77 | 1.6 | 2.62 |
|  |  |  | c2070_g1_i1 | 4.96 | 2.11 | 3 |
| K14498 | SNRK2 | serine/threonine-protein kinase SRK2 | c73755_g1_i1 | 18.51 | 38.34 | 20.15 |
|  |  |  | c81151_g1_i1 | 90.9 | 88.56 | 130.81 |
|  |  |  | c84582_g1_i1 | 27.08 | 67.21 | 46.98 |
|  |  |  | c92268_g2_i1 | 41.23 | 57.67 | 49.01 |
|  |  |  | c93020_g1_i1 | 6.33 | 3.34 | 2.64 |
|  |  |  | c93020_g1_i2 | 31.63 | 26.44 | 30.63 |
|  |  |  | c93020_g2_i1 | 45.97 | 46.01 | 28.57 |
|  |  |  | c93020_g3_i1 | 41.02 | 28.21 | 23.8 |
|  |  |  | c95053_g1_i1 | 15.98 | 12.61 | 16.11 |
|  |  |  | c95053_g2_i1 | 31.51 | 42.68 | 57.27 |
|  |  |  | c27346_g1_i1 | 22.03 | 17.51 | 17.27 |
|  |  |  | c27740_g1_i1 | 87.62 | 83.89 | 106.97 |
| K00688 | E2.4.1.1, glgP, PYG | glycogen phosphorylase | c81053_g1_i1 | 4.57 | 27.9 | 18.6 |
|  |  |  | c81053_g1_i2 | 6.83 | 2.98 | 6.16 |
|  |  |  | c84699_g1_i1 | 7.21 | 12.61 | 8.44 |
|  |  |  | c100953_g1_i1 | 1.69 | 6.13 | 2.86 |
|  |  |  | c115234_g1_i1 | 0.89 | 0.36 | 0.24 |
| K00432 | E1.11.1.9 | glutathione peroxidase | c88487_g1_i1 | 11.73 | 10.25 | 3.48 |
|  |  |  | c93501_g1_i1 | 178.08 | 67.9 | 249.33 |
|  |  |  | c94260_g1_i1 | 1.08 | 0.18 | 0.96 |
|  |  |  | c98562_g2_i1 | 303.7 | 113.59 | 158.96 |
|  |  |  | c98562_g3_i1 | 599.37 | 230.18 | 350.16 |
|  |  |  | c130995_g1_i1 | 0.6 | 0.47 | 0.65 |
|  |  |  | c137481_g1_i1 | 24.41 | 20.59 | 21.89 |
| K17279 | REEP5_6 | receptor expression-enhancing protein 5/6 | c81291_g1_i1 | 21.58 | 17.17 | 9.6 |
|  |  |  | c88982_g1_i1 | 12.19 | 4.37 | 5.92 |
|  |  |  | c88982_g2_i1 | 8.63 | 9.38 | 10.4 |
|  |  |  | c94749_g2_i1 | 0.46 | 1.44 | 0.33 |
|  |  |  | c98475_g1_i1 | 1.48 | 1.73 | 1.67 |
|  |  |  | c98475_g1_i2 | 0.78 | 1.73 | 0.3 |
|  |  |  | c151772_g1_i1 | 14.79 | 15.94 | 15.33 |
|  |  |  | c74310_g1_i1 | 5.52 | 5.65 | 17.94 |
|  |  |  | c81796_g1_i1 | 46.25 | 61.04 | 78.38 |
| K10523 | SPOP | speckle-type POZ protein | c72795_g1_i1 | 54.22 | 34.62 | 51.23 |
|  |  |  | c73944_g1_i1 | 6.95 | 6.55 | 8.47 |
|  |  |  | c82146_g1_i1 | 16.18 | 21 | 27.22 |
|  |  |  | c82146_g2_i1 | 3.68 | 9.26 | 7.74 |
|  |  |  | c85290_g1_i2 | 15.55 | 18.02 | 20.26 |
|  |  |  | c85290_g1_i3 | 39.61 | 40.77 | 58.87 |
|  |  |  | c97262_g1_i1 | 16.5 | 33.39 | 34.22 |
|  |  |  | c97262_g1_i2 | 4.56 | 8.96 | 10.08 |
|  |  |  | c97262_g2_i1 | 145.42 | 192.04 | 85.04 |
|  |  |  | c97262_g3_i1 | 19.86 | 31.25 | 35.25 |
|  |  |  | c100961_g3_i1 | 5.52 | 6.84 | 8.62 |
|  |  |  | c154151_g1_i1 | 15.88 | 19.37 | 20.69 |
|  |  |  | c59712_g1_i1 | 22.52 | 19.54 | 26.74 |
| K01177 | E3.2.1.2 | beta-amylase | c80088_g1_i1 | 2.49 | 2.96 | 3.09 |
|  |  |  | c80088_g1_i2 | 16.26 | 5.7 | 18.4 |
|  |  |  | c100002_g1_i1 | 518.14 | 177.32 | 247.35 |
|  |  |  | c101409_g1_i1 | 2.43 | 2.24 | 2.42 |
|  |  |  | c101409_g4_i1 | 15.37 | 8.27 | 11.5 |
|  |  |  | c101409_g5_i1 | 4.9 | 3.48 | 6.5 |
|  |  |  | c102292_g1_i1 | 0.82 | 0.63 | 0.84 |
|  |  |  | c102292_g1_i2 | 3.32 | 3.68 | 3.5 |
| K17095 | ANXA7_11 | annexin A7/11 | c90975_g1_i1 | 34.03 | 32.32 | 61.68 |
|  |  |  | c91774_g1_i1 | 10.54 | 0.88 | 53.85 |
|  |  |  | c91901_g1_i1 | 0.88 | 0.42 | 0.28 |
|  |  |  | c91901_g1_i3 | 0.38 | 0.46 | 0.28 |
|  |  |  | c92775_g1_i1 | 11.48 | 13.76 | 22.35 |
|  |  |  | c92775_g2_i1 | 11.51 | 12.71 | 23.11 |
|  |  |  | c101479_g2_i1 | 5.58 | 13.03 | 14.22 |
|  |  |  | c162686_g1_i1 | 46.76 | 45.12 | 49.66 |
| K14803 | PTC2_3 | protein phosphatase PTC2/3 | c91640_g1_i1 | 41.81 | 25.51 | 44.01 |
|  |  |  | c95982_g1_i1 | 15.02 | 11.98 | 23.56 |
|  |  |  | c95982_g1_i2 | 20.49 | 18.93 | 15.88 |
|  |  |  | c95982_g1_i3 | 1.37 | 4.66 | 6.3 |
|  |  |  | c98031_g1_i1 | 48.77 | 27.15 | 44.25 |
|  |  |  | c99564_g1_i1 | 8.62 | 3.45 | 6.51 |
|  |  |  | c99564_g2_i1 | 0.85 | 0.86 | 1.32 |
|  |  |  | c99564_g2_i2 | 5.47 | 5.28 | 7.8 |
|  |  |  | c99564_g2_i3 | 2.96 | 1.73 | 1.27 |
|  |  |  | c127404_g1_i1 | 7.82 | 5.74 | 8.91 |
| K09250 | CNBP | cellular nucleic acid-binding protein | c92409_g1_i1 | 21.14 | 21.38 | 24.23 |
|  |  |  | c92409_g1_i2 | 4.26 | 5.95 | 2.89 |
|  |  |  | c92409_g2_i1 | 33.41 | 20.1 | 27.38 |
|  |  |  | c92409_g2_i2 | 7.78 | 0.73 | 4.35 |
|  |  |  | c93811_g1_i1 | 18.59 | 11.85 | 11.12 |
|  |  |  | c93811_g1_i3 | 1.68 | 0.85 | 4.61 |
|  |  |  | c127206_g1_i1 | 14.26 | 17.7 | 16.65 |
|  |  |  | c57820_g1_i1 | 147.53 | 92.24 | 157.08 |
| K16911 | DDX21 | ATP-dependent RNA helicase DDX21 | c69620_g1_i1 | 3.24 | 5.04 | 5.48 |
|  |  |  | c102005_g1_i1 | 93.87 | 71.99 | 91.54 |
|  |  |  | c65073_g1_i1 | 3.81 | 11.81 | 11.03 |
| K09840 | NCED | 9-cis-epoxycarotenoid dioxygenase | c68951_g1_i1 | 0.14 | 0.4 | 0.61 |
|  |  |  | c81822_g2_i1 | 1.62 | 1.57 | 0.6 |
|  |  |  | c81822_g3_i1 | 0.74 | 2.06 | 2.86 |
|  |  |  | c91787_g1_i1 | 393.58 | 193.52 | 260.02 |
|  |  |  | c98319_g1_i1 | 2.65 | 0.67 | 0.83 |
|  |  |  | c123586_g1_i1 | 0.9 | 0.69 | 0.49 |
| K16277 | DRIP | E3 ubiquitin-protein ligase DRIP | c93171_g1_i1 | 4.79 | 14.24 | 4.47 |
|  |  |  | c93171_g2_i1 | 1.36 | 3.17 | 3.62 |
|  |  |  | c93171_g3_i1 | 3.58 | 3.99 | 0.21 |
|  |  |  | c93171_g4_i1 | 2.93 | 2.26 | 4.12 |
|  |  |  | c93464_g1_i2 | 0.9 | 2.76 | 2.08 |
|  |  |  | c99131_g1_i1 | 9.32 | 8.24 | 8.94 |
| K11254 | H4 | histone H4 | c98213_g1_i1 | 73.31 | 160.41 | 255.79 |
|  |  |  | c98213_g1_i3 | 31.48 | 24.75 | 27.44 |
|  |  |  | c98213_g1_i4 | 163.64 | 222.42 | 221.7 |
|  |  |  | c59505_g2_i1 | 101.41 | 116.85 | 106.1 |
| K13422 | MYC2 | transcription factor MYC2 | c96739_g1_i1 | 19.66 | 22.67 | 44.58 |
|  |  |  | c96739_g2_i1 | 17.45 | 12.89 | 30.11 |
|  |  |  | c96739_g3_i1 | 8.89 | 9.07 | 17.27 |
|  |  |  | c101691_g3_i1 | 14.99 | 18.02 | 22.29 |
|  |  |  | c101691_g3_i2 | 4.85 | 2.09 | 1.25 |
|  |  |  | c101691_g3_i3 | 34.87 | 23.97 | 49.44 |
|  |  |  | c23238_g1_i1 | 6.09 | 8.13 | 7.64 |
|  |  |  | c23238_g2_i1 | 5.75 | 6.19 | 5.07 |
| K00511 | SQLE, ERG1 | squalene monooxygenase | c87389_g1_i1 | 2.36 | 0.58 | 1.71 |
|  |  |  | c87389_g2_i1 | 1.88 | 1.08 | 2.05 |
|  |  |  | c88756_g2_i1 | 4.99 | 6.26 | 1.36 |
|  |  |  | c99211_g2_i1 | 23.87 | 14.54 | 17.27 |
| K17679 | MSS116 | ATP-dependent RNA helicase MSS116, mitochondrial | c91503_g1_i1 | 5.15 | 1.39 | 1.18 |
|  |  |  | c91503_g2_i1 | 1.41 | 2.61 | 1.19 |
|  |  |  | c98437_g2_i1 | 37.79 | 27.25 | 37.01 |
| K06268 | PPP3R, CNB | serine/threonine-protein phosphatase 2B regulatory subunit | c91877_g1_i1 | 36.64 | 24.17 | 30.21 |
|  |  |  | c92294_g1_i1 | 5.36 | 4.21 | 3.71 |
|  |  |  | c101619_g2_i1 | 2.18 | 6.04 | 3.2 |
|  |  |  | c106524_g1_i1 | 31.22 | 29.94 | 29.97 |
|  |  |  | c162911_g1_i1 | 20.83 | 15.68 | 20.79 |
| K12885 | RBMX, HNRNPG | heterogeneous nuclear ribonucleoprotein G | c83071_g2_i1 | 9.37 | 5.3 | 6.24 |
|  |  |  | c83071_g2_i2 | 58.01 | 33.13 | 41.87 |
|  |  |  | c99984_g1_i1 | 7.36 | 8.17 | 11.06 |
|  |  |  | c99984_g1_i2 | 29.91 | 23.61 | 26.07 |
| K08232 | E1.6.5.4 | monodehydroascorbate reductase (NADH) | c71144_g1_i1 | 18.41 | 10.97 | 17.45 |
|  |  |  | c96213_g1_i1 | 82.22 | 41.14 | 65.46 |
|  |  |  | c96998_g3_i1 | 62.91 | 109.67 | 85.24 |
|  |  |  | c96998_g4_i1 | 123.94 | 81.79 | 97.37 |
| K03627 | MBF1 | putative transcription factor | c89358_g1_i2 | 155.32 | 289.6 | 365 |
|  |  |  | c127959_g1_i1 | 95.56 | 14.66 | 44.58 |
| K11713 | PGTB1 | geranylgeranyl transferase type-1 subunit beta | c100608_g1_i1 | 0.11 | 0.58 | 0.94 |
|  |  |  | c100608_g1_i3 | 2.1 | 3.36 | 1.8 |
|  |  |  | c100608_g1_i4 | 2.41 | 0.71 | 0.18 |
| K17991 | PXG | peroxygenase | c79859_g1_i1 | 0.87 | 5.94 | 5.07 |
|  |  |  | c92771_g1_i1 | 13.08 | 13.67 | 10.65 |
| K06634 | CCNH | cyclin H | c81073_g1_i1 | 2.35 | 5.5 | 4.51 |
| K12118 | CRY1 | cryptochrome 1 | c57741_g1_i1 | 159.04 | 182.76 | 195.8 |
| K05955 | FNTA | protein farnesyltransferase/geranylgeranyltransferase type-1 subunit alpha | c96923_g1_i1 | 11.3 | 9.37 | 12.43 |
| K05954 | FNTB | protein farnesyltransferase subunit beta | c87685_g1_i1 | 12.37 | 13.87 | 11.12 |
