# Supplementary material for: Transcriptome Analysis of Cinnamomum chago: A Revelation of Candidate Genes for Abiotic Stress Response and Terpenoid and Fatty Acid Biosyntheses
Source: Front Genet. 2018 Nov 5;9:505. doi: 10.3389/fgene.2018.00505 (PMC6231050; doi:10.3389/fgene.2018.00505)
Supplement: Supplementary file 14 [file Table_9.DOC]

***Supplementary Material***

**Characterization of the de novo *Cinnamomum chago* (Lauraceae) transcriptome reveals candidate genes for terpenoid, fatty acid biosyntheses and abiotic stress**

**Authors:** Xue Zhang, Shi-Kang Shen *,

***Address for Correspondence:** Shi-Kang Shen, School of Life Sciences, Yunnan University, No. 2 Green lake North road Kunming, Yunnan, 650091, the People’s Republic of China. Telephone:+86-871-65031412; Fax:+86-871-65031412;

**E-mail:** yunda123456@126.com

**Table S9 Candidate genes related to cold stress in *C. chago*** transcriptome

| **KO ID** | **Gene** | **KEGG Annotation** | **Numbers of unineges** |
| --- | --- | --- | --- |
| K00799 | GST, gst | glutathione S-transferase | 28 |
| K03283 | HSPA1_8 | heat shock 70kDa protein 1/8 | 32 |
| K03781 | katE, CAT, catB, srpA | catalase | 19 |
| K13448 | CML | calcium-binding protein CML | 33 |
| K09286 | EREBP | EREBP-like factor | 32 |
| K04077 | groEL, HSPD1 | chaperonin GroEL | 16 |
| K00249 | ACADM, acd | acyl-CoA dehydrogenase | 5 |
| K01115 | PLD1_2 | phospholipase D1/2 | 19 |
| K00600 | glyA, SHMT | glycine hydroxymethyltransferase | 13 |
| K00927 | PGK, pgk | phosphoglycerate kinase | 9 |
| K07375 | TUBB | tubulin beta | 19 |
| K01673 | cynT, can | carbonic anhydrase | 9 |
| K00434 | E1.11.1.11 | L-ascorbate peroxidase | 10 |
| K15397 | KCS | [3-ketoacyl-CoA synthase [EC:2.3.1.199]](http://www.genome.jp/dbget-bin/www_bget?ec:2.3.1.199) | 14 |
| K00695 | E2.4.1.13 | sucrose synthase | 9 |
| K00789 | metK | S-adenosylmethionine synthetase | 5 |
| K09487 | HSP90B, TRA1 | heat shock protein 90kDa beta | 12 |
| K10839 | RAD23, HR23 | UV excision repair protein RAD23 | 10 |
| K00026 | MDH2 | malate dehydrogenase | 13 |
| K04078 | groES, HSPE1 | chaperonin GroES | 10 |
| K11294 | NCL, NSR1 | nucleolin | 11 |
| K05298 | GAPA | glyceraldehyde-3-phosphate dehydrogenase (NADP+) (phosphorylating) | 7 |
| K05592 | deaD, cshA | ATP-dependent RNA helicase DeaD | 1 |
| K17279 | REEP5_6 | receptor expression-enhancing protein 5/6 | 9 |
| K01177 | E3.2.1.2 | beta-amylase | 8 |
| K01602 | rbcS | ribulose-bisphosphate carboxylase small chain | 6 |
| K03841 | FBP, fbp | fructose-1,6-bisphosphatase I | 5 |
| K01835 | pgm | phosphoglucomutase | 7 |
| K00855 | PRK, prkB | phosphoribulokinase | 7 |
| K17095 | ANXA7_11 | annexin A7/11 | 8 |
| K01759 | GLO1, gloA | lactoylglutathione lyase | 6 |
| K04371 | MAPK1_3 | mitogen-activated protein kinase 1/3 | 1 |
| K14803 | PTC2_3 | protein phosphatase PTC2/3 | 10 |
| K09250 | CNBP | cellular nucleic acid-binding protein | 8 |
| K16911 | DDX21 | ATP-dependent RNA helicase DDX21 | 3 |
| K00392 | sir | sulfite reductase (ferredoxin) | 8 |
| K03234 | EEF2 | elongation factor 2 | 2 |
| K02893 | RP-L23Ae, RPL23A | large subunit ribosomal protein L23Ae | 2 |
| K10257 | FAD8, desB | acyl-lipid omega-3 desaturase | 4 |
| K01783 | rpe, RPE | ribulose-phosphate 3-epimerase | 3 |
| K03386 | E1.11.1.15, PRDX, ahpC | peroxiredoxin (alkyl hydroperoxide reductase subunit C) | 3 |
| K08486 | STX1B_2_3 | syntaxin 1B/2/3 | 3 |
| K18980 | EO | cinnamoyl-CoA reductase | 3 |
| K12855 | PRPF6, PRP6 | pre-mRNA-processing factor 6 | 5 |
| K02988 | RP-S5, MRPS5, rpsE | small subunit ribosomal protein S5 | 2 |
| K17679 | MSS116 | ATP-dependent RNA helicase MSS116, mitochondrial | 3 |
| K06268 | PPP3R, CNB | serine/threonine-protein phosphatase 2B regulatory subunit | 5 |
| K12885 | RBMX, HNRNPG | heterogeneous nuclear ribonucleoprotein G | 4 |
| K02888 | RP-L21, MRPL21, rplU | large subunit ribosomal protein L21 | 2 |
| K02898 | RP-L26e, RPL26 | large subunit ribosomal protein L26e | 4 |
| K02941 | RP-LP0, RPLP0 | large subunit ribosomal protein LP0 | 2 |
| K03098 | APOD | apolipoprotein D and lipocalin family protein | 2 |
| K15633 | gpmI | 2,3-bisphosphoglycerate-independent phosphoglycerate mutase | 1 |
| K03146 | THI4, THI1 | thiamine thiazole synthase | 4 |
| K02150 | ATPeV1E, ATP6E | V-type H+-transporting ATPase subunit E | 3 |
| K09753 | CCR | cinnamoyl-CoA reductase | 1 |
| K17991 | PXG | peroxygenase | 2 |
| K02870 | RP-L12e, RPL12 | large subunit ribosomal protein L12e | 2 |
| K08762 | DBI, ACBP | diazepam-binding inhibitor (GABA receptor modulator, acyl-CoA-binding protein) | 1 |
| K04124 | E1.14.11.15 | gibberellin 3-beta-dioxygenase | 1 |
| K08244 | E2.7.9.4 | alpha-glucan, water dikinase | 2 |
| K12121 | PHYB | phytochrome B | 1 |
| K10047 | VTC4 | inositol-phosphate phosphatase / L-galactose 1-phosphate phosphatase | 1 |
| K12124 | GI | GIGANTEA | 1 |
| K02516 | PRMT5, HSL7 | type II protein arginine methyltransferase | 1 |
| K04688 | RPS6KB | ribosomal protein S6 kinase beta | 1 |
| K12667 | SWP1, RPN2 | oligosaccharyltransferase complex subunit delta (ribophorin II) | 1 |
| K01583 | E4.1.1.19 | arginine decarboxylase | 1 |
| K11462 | EED | polycomb protein EED | 1 |
| K15135 | MED18 | mediator of RNA polymerase II transcription subunit 18 | 1 |
| K02727 | PSMA3 | 20S proteasome subunit alpha 7 | 1 |
| All |  | 72 | 498 |
