# Supplementary material for: Transcriptome Analysis of Cinnamomum chago: A Revelation of Candidate Genes for Abiotic Stress Response and Terpenoid and Fatty Acid Biosyntheses
Source: Front Genet. 2018 Nov 5;9:505. doi: 10.3389/fgene.2018.00505 (PMC6231050; doi:10.3389/fgene.2018.00505)
Supplement: Supplementary file 15 [file Table_10.DOC]

***Supplementary Material***

**Characterization of the de novo *Cinnamomum chago* (Lauraceae) transcriptome reveals candidate genes for terpenoid, fatty acid biosyntheses and abiotic stress**

**Authors:** Xue Zhang, Shi-Kang Shen *,

***Address for Correspondence:** Shi-Kang Shen, School of Life Sciences, Yunnan University, No. 2 Green lake North road Kunming, Yunnan, 650091, the People’s Republic of China. Telephone:+86-871-65031412; Fax:+86-871-65031412;

**E-mail:** yunda123456@126.com

**Table S10 The FPKM values of candidate genes related to cold stress in *C. chago* transcriptome**

| KO ID | Gene ID | KEGG Annotation | Unigene | Ccg1 | Ccg2 |
| --- | --- | --- | --- | --- | --- |
| K00799 | GST, gst | glutathione S-transferase | c70826_g1_i1 | 0.52 | 1.25 |
|  |  |  | c71073_g1_i1 | 12.88 | 0.88 |
|  |  |  | c73020_g1_i1 | 72.03 | 97.69 |
|  |  |  | c75769_g1_i1 | 92.03 | 115.08 |
|  |  |  | c75886_g1_i1 | 22.72 | 29.39 |
|  |  |  | c75886_g1_i2 | 4.79 | 8.52 |
|  |  |  | c77125_g1_i1 | 131.03 | 79.81 |
|  |  |  | c84041_g1_i1 | 801.76 | 247.15 |
|  |  |  | c89710_g2_i1 | 5.16 | 4.68 |
|  |  |  | c92981_g1_i1 | 37.8 | 7.23 |
|  |  |  | c92981_g2_i1 | 6.49 | 3.25 |
|  |  |  | c94020_g2_i1 | 15.3 | 0.79 |
|  |  |  | c95620_g1_i1 | 10.7 | 3.96 |
|  |  |  | c99082_g1_i1 | 588.11 | 233.58 |
|  |  |  | c99342_g1_i1 | 1.59 | 3.87 |
|  |  |  | c100118_g1_i1 | 861.29 | 514.4 |
|  |  |  | c100278_g1_i1 | 3.72 | 4.77 |
|  |  |  | c100278_g2_i1 | 14.28 | 11.5 |
|  |  |  | c100487_g3_i1 | 108.45 | 115.79 |
|  |  |  | c101386_g1_i10 | 0.55 | 0.27 |
|  |  |  | c101990_g1_i1 | 62.32 | 65.14 |
|  |  |  | c101990_g1_i2 | 15.98 | 5.59 |
|  |  |  | c102275_g1_i1 | 88.24 | 56.78 |
|  |  |  | c102275_g1_i2 | 350.13 | 271.29 |
|  |  |  | c127769_g1_i1 | 125.51 | 62.71 |
|  |  |  | c39637_g1_i1 | 0.72 | 0.28 |
|  |  |  | c42301_g2_i1 | 3.23 | 2.24 |
|  |  |  | c66827_g1_i1 | 627.17 | 264.65 |
| K03283 | HSPA1_8 | heat shock 70kDa protein 1/8 | c73857_g1_i1 | 2.58 | 2.2 |
|  |  |  | c76923_g1_i1 | 4.66 | 7.2 |
|  |  |  | c76923_g2_i1 | 3.85 | 5.95 |
|  |  |  | c78399_g1_i1 | 13.06 | 22.1 |
|  |  |  | c83768_g1_i1 | 110.14 | 27.59 |
|  |  |  | c86493_g1_i1 | 118.35 | 24.4 |
|  |  |  | c89061_g1_i1 | 0.44 | 7.95 |
|  |  |  | c89061_g2_i1 | 0.65 | 5.59 |
|  |  |  | c90232_g1_i1 | 15.36 | 4.08 |
|  |  |  | c90512_g1_i3 | 4.52 | 4.96 |
|  |  |  | c91709_g1_i1 | 0.38 | 1.81 |
|  |  |  | c91709_g1_i2 | 3.3 | 2.38 |
|  |  |  | c91709_g1_i2 | 3.3 | 2.38 |
|  |  |  | c91709_g3_i1 | 10.86 | 4.51 |
|  |  |  | c92905_g1_i1 | 3.22 | 3.56 |
|  |  |  | c94933_g1_i1 | 5.97 | 12.07 |
|  |  |  | c95039_g1_i1 | 5.55 | 2.74 |
|  |  |  | c95569_g1_i1 | 55.44 | 46.47 |
|  |  |  | c96579_g1_i3 | 4.09 | 11.95 |
|  |  |  | c96579_g2_i1 | 1.59 | 15.17 |
|  |  |  | c100436_g1_i1 | 2.01 | 6.94 |
|  |  |  | c101374_g2_i1 | 148.45 | 120.74 |
|  |  |  | c102006_g1_i1 | 27.68 | 42.75 |
|  |  |  | c102006_g2_i1 | 437.49 | 214.07 |
|  |  |  | c102006_g2_i2 | 170.72 | 96.57 |
|  |  |  | c102006_g3_i1 | 37.43 | 36.82 |
|  |  |  | c102006_g3_i2 | 215.85 | 132.22 |
|  |  |  | c102006_g3_i3 | 26.35 | 37.81 |
|  |  |  | c127697_g1_i1 | 1.39 | 0.22 |
|  |  |  | c139597_g1_i1 | 97.29 | 17.38 |
|  |  |  | c166823_g1_i1 | 96.19 | 48.58 |
|  |  |  | c18497_g1_i1 | 61.83 | 90.02 |
| K03781 | katE, CAT, catB, srpA | catalase | c75996_g1_i1 | 0.48 | 0.47 |
|  |  |  | c77804_g1_i1 | 0.83 | 0.17 |
|  |  |  | c79793_g1_i1 | 20.79 | 22.17 |
|  |  |  | c79793_g3_i1 | 12.1 | 18.08 |
|  |  |  | c94027_g1_i1 | 1006.53 | 401.2 |
|  |  |  | c94027_g2_i1 | 420.98 | 693.93 |
|  |  |  | c100576_g2_i3 | 6.44 | 3.91 |
|  |  |  | c101144_g1_i5 | 0.09 | 1.09 |
|  |  |  | c101144_g1_i7 | 0.38 | 1.2 |
|  |  |  | c101386_g1_i1 | 3.38 | 1.75 |
|  |  |  | c101386_g1_i11 | 3.9 | 5.61 |
|  |  |  | c101386_g1_i2 | 1.61 | 14.3 |
|  |  |  | c101386_g1_i8 | 2.09 | 5.35 |
|  |  |  | c106445_g1_i1 | 0.53 | 0.63 |
|  |  |  | c118984_g1_i1 | 1.97 | 4.17 |
|  |  |  | c159383_g1_i1 | 1.03 | 2.23 |
|  |  |  | c164774_g1_i1 | 1.15 | 0.83 |
|  |  |  | c64598_g1_i1 | 0.34 | 0.56 |
|  |  |  | c66779_g1_i1 | 0.16 | 0.54 |
| K13448 | CML | calcium-binding protein CML | c69769_g1_i1 | 1.48 | 1.72 |
|  |  |  | c72868_g1_i1 | 11.59 | 7.27 |
|  |  |  | c74070_g1_i2 | 1.61 | 2.13 |
|  |  |  | c75983_g1_i1 | 4.94 | 4.34 |
|  |  |  | c76630_g1_i1 | 12.46 | 13.6 |
|  |  |  | c78431_g1_i1 | 0.64 | 0.99 |
|  |  |  | c84735_g1_i1 | 6.2 | 20.89 |
|  |  |  | c85289_g2_i1 | 7.22 | 5.58 |
|  |  |  | c85289_g2_i2 | 17.16 | 12.33 |
|  |  |  | c85865_g1_i1 | 206.31 | 165.49 |
|  |  |  | c88908_g1_i1 | 28.58 | 35.81 |
|  |  |  | c94476_g1_i1 | 24.02 | 12.04 |
|  |  |  | c94476_g2_i1 | 21.85 | 14.35 |
|  |  |  | c95343_g1_i1 | 3.91 | 3.83 |
|  |  |  | c98478_g1_i1 | 0.46 | 0.3 |
|  |  |  | c98478_g1_i2 | 0.79 | 2.05 |
|  |  |  | c98478_g1_i3 | 5.11 | 10.28 |
|  |  |  | c98801_g1_i2 | 10.48 | 9.86 |
|  |  |  | c98801_g1_i3 | 3.99 | 2.43 |
|  |  |  | c101976_g2_i1 | 7.61 | 3.45 |
|  |  |  | c101976_g2_i2 | 0.67 | 0.29 |
|  |  |  | c114035_g1_i1 | 79.42 | 69.55 |
|  |  |  | c115494_g1_i1 | 6.91 | 7.19 |
|  |  |  | c121284_g1_i1 | 0.74 | 1.36 |
|  |  |  | c150902_g1_i1 | 29.04 | 43.23 |
|  |  |  | c164220_g1_i1 | 107.3 | 105.9 |
|  |  |  | c165177_g1_i1 | 100.72 | 42.28 |
|  |  |  | c1946_g1_i1 | 2.54 | 10.16 |
|  |  |  | c2467_g1_i1 | 2.56 | 0.25 |
|  |  |  | c24929_g1_i1 | 3.47 | 3.92 |
|  |  |  | c58075_g1_i1 | 3.08 | 0.83 |
|  |  |  | c61200_g1_i1 | 1.69 | 0.47 |
|  |  |  | c64361_g3_i1 | 57.02 | 54.3 |
| K09286 | EREBP | EREBP-like factor | c68179_g1_i1 | 0.96 | 0.54 |
|  |  |  | c69316_g2_i1 | 66.8 | 52.81 |
|  |  |  | c74214_g1_i1 | 24.94 | 16.02 |
|  |  |  | c74214_g2_i1 | 23.18 | 17.49 |
|  |  |  | c77451_g1_i1 | 43.51 | 44.61 |
|  |  |  | c78299_g2_i1 | 1.58 | 16.8 |
|  |  |  | c81390_g1_i1 | 1.32 | 0.74 |
|  |  |  | c84324_g1_i1 | 3.94 | 12.21 |
|  |  |  | c84367_g1_i1 | 5.56 | 7.41 |
|  |  |  | c84627_g1_i1 | 15.21 | 15.85 |
|  |  |  | c88114_g2_i1 | 6.86 | 11.67 |
|  |  |  | c92630_g1_i1 | 134.13 | 131.51 |
|  |  |  | c92784_g1_i1 | 5.75 | 4.23 |
|  |  |  | c92806_g1_i1 | 11.89 | 8.32 |
|  |  |  | c94758_g1_i1 | 24.05 | 13.41 |
|  |  |  | c94862_g2_i1 | 1.63 | 1.23 |
|  |  |  | c96312_g4_i1 | 61.67 | 62.64 |
|  |  |  | c98828_g1_i1 | 170.75 | 306.68 |
|  |  |  | c98939_g1_i2 | 83.25 | 149.62 |
|  |  |  | c100057_g1_i1 | 268.74 | 367.18 |
|  |  |  | c100057_g1_i2 | 22.31 | 27.11 |
|  |  |  | c100057_g2_i1 | 42.2 | 47.59 |
|  |  |  | c119107_g1_i1 | 3.65 | 0.37 |
|  |  |  | c137498_g1_i1 | 206.65 | 202.16 |
|  |  |  | c137676_g1_i1 | 27.83 | 16.63 |
|  |  |  | c142150_g1_i1 | 1.52 | 15.3 |
|  |  |  | c146383_g1_i1 | 0.53 | 1.02 |
|  |  |  | c150972_g1_i1 | 44.39 | 34.22 |
|  |  |  | c16438_g1_i1 | 34.26 | 30.94 |
|  |  |  | c59312_g1_i1 | 2.43 | 9.57 |
|  |  |  | c59312_g2_i1 | 1.64 | 3.04 |
|  |  |  | c80244_g1_i1 | 118.52 | 96.54 |
| K04077 | groEL, HSPD1 | chaperonin GroEL | c93571_g1_i1 | 15.68 | 7.36 |
|  |  |  | c93571_g2_i1 | 13.07 | 7.53 |
|  |  |  | c94339_g1_i1 | 0.78 | 1.03 |
|  |  |  | c94339_g2_i1 | 2.29 | 1.46 |
|  |  |  | c94339_g3_i1 | 0.15 | 1.25 |
|  |  |  | c94339_g5_i2 | 1.08 | 0.89 |
|  |  |  | c94339_g5_i3 | 0.51 | 0.24 |
|  |  |  | c101013_g1_i1 | 132.45 | 54.43 |
|  |  |  | c101013_g1_i2 | 107.2 | 83.57 |
|  |  |  | c101013_g1_i3 | 74.33 | 53.93 |
|  |  |  | c101013_g1_i4 | 92.13 | 74.4 |
|  |  |  | c102770_g4_i1 | 0.55 | 0.96 |
|  |  |  | c127266_g1_i1 | 0.49 | 1.03 |
|  |  |  | c162882_g1_i1 | 6.24 | 5.25 |
|  |  |  | c38676_g1_i1 | 0.9 | 1.39 |
|  |  |  | c39081_g1_i1 | 4.4 | 1.54 |
| K00249 | ACADM, acd | acyl-CoA dehydrogenase | c99047_g1_i1 | 1.29 | 0.07 |
|  |  |  | c99895_g1_i1 | 12.43 | 15 |
|  |  |  | c99047_g1_i1 | 1.29 | 0.07 |
|  |  |  | c99895_g1_i1 | 12.43 | 15 |
|  |  |  | c37530_g1_i2 | 1.21 | 0.08 |
| K01115 | PLD1_2 | phospholipase D1/2 | c74421_g1_i1 | 33.5 | 15.5 |
|  |  |  | c74421_g3_i1 | 18.88 | 9.65 |
|  |  |  | c74421_g4_i1 | 39.44 | 15.1 |
|  |  |  | c77873_g1_i1 | 17.11 | 38.24 |
|  |  |  | c79535_g2_i1 | 3.41 | 0.84 |
|  |  |  | c79535_g3_i1 | 0.1 | 1.04 |
|  |  |  | c80516_g1_i1 | 1.17 | 0.74 |
|  |  |  | c85785_g1_i1 | 42.02 | 65.4 |
|  |  |  | c88850_g2_i1 | 1.18 | 2.89 |
|  |  |  | c88850_g3_i1 | 0.26 | 0.48 |
|  |  |  | c88850_g3_i2 | 3.09 | 2.37 |
|  |  |  | c88850_g3_i3 | 0.73 | 2.02 |
|  |  |  | c91594_g1_i1 | 17.42 | 20.03 |
|  |  |  | c102527_g2_i1 | 2.2 | 4.92 |
|  |  |  | c102527_g2_i3 | 0.12 | 1.25 |
|  |  |  | c102527_g2_i4 | 2.36 | 2.12 |
|  |  |  | c102527_g3_i2 | 1.79 | 0.91 |
|  |  |  | c102937_g1_i1 | 35.28 | 11.65 |
|  |  |  | c18179_g1_i2 | 0.85 | 0.3 |
| K00600 | glyA, SHMT | glycine hydroxymethyltransferase | c70179_g1_i1 | 11.31 | 14.45 |
|  |  |  | c70179_g2_i1 | 15.31 | 12.44 |
|  |  |  | c86210_g1_i1 | 1.93 | 0.74 |
|  |  |  | c95603_g1_i1 | 25.33 | 22.64 |
|  |  |  | c98979_g1_i1 | 2.89 | 1.52 |
|  |  |  | c99035_g2_i1 | 244.14 | 189.21 |
|  |  |  | c101417_g1_i1 | 61.85 | 61.64 |
|  |  |  | c102050_g1_i1 | 16.22 | 17.44 |
|  |  |  | c114375_g1_i1 | 0.54 | 0.41 |
|  |  |  | c125547_g1_i1 | 1.24 | 0.44 |
|  |  |  | c125832_g1_i1 | 0.76 | 1.82 |
|  |  |  | c153160_g1_i1 | 20.63 | 6.93 |
|  |  |  | c31704_g1_i1 | 4.46 | 3.07 |
| K00927 | PGK, pgk | phosphoglycerate kinase | c84792_g1_i1 | 0.55 | 0.89 |
|  |  |  | c97652_g1_i1 | 118.67 | 52.78 |
|  |  |  | c101030_g1_i1 | 5.86 | 2.07 |
|  |  |  | c101030_g1_i2 | 0.96 | 0.5 |
|  |  |  | c101349_g1_i1 | 0.07 | 1.23 |
|  |  |  | c101349_g1_i2 | 1.94 | 0.68 |
|  |  |  | c150764_g1_i1 | 673.56 | 233.62 |
|  |  |  | c41265_g1_i1 | 2.41 | 0.93 |
|  |  |  | c51491_g1_i1 | 0.6 | 0.8 |
| K07375 | TUBB | tubulin beta | c70694_g1_i1 | 2.77 | 8.74 |
|  |  |  | c70694_g3_i1 | 3.59 | 16.05 |
|  |  |  | c97387_g1_i1 | 6.71 | 7.82 |
|  |  |  | c100226_g4_i1 | 6.59 | 5.77 |
|  |  |  | c100226_g5_i10 | 0.37 | 5.18 |
|  |  |  | c100226_g5_i5 | 8.05 | 5.57 |
|  |  |  | c101655_g1_i1 | 11.03 | 19.17 |
|  |  |  | c101655_g1_i2 | 59.54 | 37.32 |
|  |  |  | c101655_g1_i3 | 25.97 | 16.25 |
|  |  |  | c101655_g1_i4 | 2.46 | 5.44 |
|  |  |  | c101655_g1_i5 | 6.39 | 9.44 |
|  |  |  | c101655_g2_i1 | 5.06 | 6.94 |
|  |  |  | c101655_g2_i2 | 1.16 | 1.01 |
|  |  |  | c101655_g3_i1 | 51.97 | 50.12 |
|  |  |  | c101655_g4_i1 | 2.76 | 3.25 |
|  |  |  | c101655_g4_i2 | 58.83 | 51.68 |
|  |  |  | c101655_g5_i1 | 9.08 | 55.53 |
|  |  |  | c101655_g6_i1 | 83.13 | 59.28 |
|  |  |  | c103175_g1_i1 | 1.13 | 2.5 |
| K01673 | cynT, can | carbonic anhydrase | c71575_g1_i1 | 1.93 | 0.74 |
|  |  |  | c77642_g1_i2 | 2.01 | 0.54 |
|  |  |  | c90492_g1_i1 | 1737.25 | 1628.76 |
|  |  |  | c90492_g1_i2 | 84.44 | 140.45 |
|  |  |  | c99666_g1_i1 | 9.09 | 5.72 |
|  |  |  | c100484_g1_i1 | 1.6 | 1.76 |
|  |  |  | c144833_g1_i1 | 1.08 | 1.55 |
|  |  |  | c15442_g1_i1 | 186.44 | 283.04 |
|  |  |  | c24163_g2_i1 | 0.49 | 0.41 |
| K00434 | E1.11.1.11 | L-ascorbate peroxidase | c88260_g1_i2 | 24.82 | 6.26 |
|  |  |  | c93660_g1_i1 | 24.66 | 21.03 |
|  |  |  | c93660_g1_i2 | 59.7 | 28.18 |
|  |  |  | c96754_g1_i1 | 220.62 | 182.76 |
|  |  |  | c102397_g3_i2 | 38.4 | 15.49 |
|  |  |  | c102397_g3_i3 | 0.54 | 0.88 |
|  |  |  | c103478_g2_i4 | 9.18 | 20.24 |
|  |  |  | c104426_g1_i1 | 504.88 | 182.31 |
|  |  |  | c114933_g1_i1 | 0.19 | 0.4 |
|  |  |  | c17319_g1_i1 | 54.17 | 40.73 |
| K15397 | KCS | [3-ketoacyl-CoA synthase [EC:2.3.1.199]](http://www.genome.jp/dbget-bin/www_bget?ec:2.3.1.199) | c79659_g1_i1 | 0.44 | 6.65 |
|  |  |  | c79953_g1_i1 | 1.24 | 0.68 |
|  |  |  | c90393_g1_i1 | 6.15 | 33.33 |
|  |  |  | c90930_g2_i1 | 0.57 | 0.89 |
|  |  |  | c101233_g1_i1 | 2.53 | 14.48 |
|  |  |  | c102285_g1_i2 | 1.21 | 1.42 |
|  |  |  | c102285_g3_i1 | 7.95 | 6.25 |
|  |  |  | c103027_g3_i1 | 2.5 | 2.18 |
|  |  |  | c163716_g1_i1 | 24.32 | 19.62 |
|  |  |  | c4808_g1_i1 | 0.06 | 1.77 |
|  |  |  | c51178_g1_i1 | 104.65 | 33.85 |
|  |  |  | c59480_g1_i1 | 23.77 | 86.57 |
|  |  |  | c59562_g1_i1 | 3.23 | 23.11 |
|  |  |  | c59562_g2_i1 | 4.62 | 27.97 |
| K00695 | E2.4.1.13 | sucrose synthase | c80764_g2_i1 | 5.79 | 20.77 |
|  |  |  | c82601_g1_i1 | 8.47 | 10.51 |
|  |  |  | c82601_g2_i1 | 4.3 | 19.49 |
|  |  |  | c86943_g1_i1 | 0.09 | 1.15 |
|  |  |  | c93824_g1_i1 | 78.42 | 132.33 |
|  |  |  | c100564_g2_i1 | 17.87 | 31.51 |
|  |  |  | c100564_g3_i1 | 73.77 | 161.54 |
|  |  |  | c13747_g1_i2 | 9.89 | 17 |
|  |  |  | c46136_g2_i1 | 17.82 | 13.1 |
| K00789 | metK | S-adenosylmethionine synthetase | c95071_g2_i1 | 0.42 | 0.07 |
|  |  |  | c100762_g5_i1 | 15.8 | 18.98 |
|  |  |  | c100762_g6_i1 | 204.87 | 143.84 |
|  |  |  | c111147_g1_i1 | 0.47 | 0.36 |
|  |  |  | c151719_g1_i1 | 19.57 | 9.11 |
| K09487 | HSP90B, TRA1 | heat shock protein 90kDa beta | c87175_g1_i1 | 1.74 | 1.12 |
|  |  |  | c87175_g2_i1 | 2.31 | 0.87 |
|  |  |  | c87589_g1_i1 | 20.95 | 46.03 |
|  |  |  | c88380_g1_i1 | 3.08 | 1.99 |
|  |  |  | c88380_g1_i2 | 1.74 | 0.55 |
|  |  |  | c100349_g1_i1 | 10.08 | 34.57 |
|  |  |  | c100349_g1_i2 | 23.68 | 44.03 |
|  |  |  | c100349_g1_i3 | 12.08 | 41.17 |
|  |  |  | c152123_g1_i1 | 10.01 | 16.45 |
|  |  |  | c153841_g1_i1 | 16.92 | 22.96 |
|  |  |  | c53211_g1_i1 | 23.19 | 17.5 |
|  |  |  | c53211_g2_i1 | 15.74 | 12.6 |
| K10839 | RAD23, HR23 | UV excision repair protein RAD23 | c80772_g1_i1 | 42.45 | 40.61 |
|  |  |  | c90659_g1_i3 | 0.73 | 0.48 |
|  |  |  | c93424_g1_i1 | 34.52 | 39.67 |
|  |  |  | c94671_g1_i1 | 0.13 | 49.4 |
|  |  |  | c94671_g1_i2 | 87.15 | 36.63 |
|  |  |  | c100331_g5_i1 | 5.86 | 5.85 |
|  |  |  | c100331_g5_i2 | 1.25 | 2.82 |
|  |  |  | c100331_g5_i3 | 3.25 | 3.03 |
|  |  |  | c100331_g6_i1 | 14.77 | 19.48 |
|  |  |  | c101132_g1_i5 | 3.44 | 4.3 |
| K00026 | MDH2 | malate dehydrogenase | c68904_g1_i1 | 32.92 | 2.25 |
|  |  |  | c85125_g1_i1 | 59.64 | 8.15 |
|  |  |  | c85125_g2_i1 | 116.64 | 30.29 |
|  |  |  | c88925_g2_i1 | 8.06 | 4.8 |
|  |  |  | c92641_g1_i1 | 8.96 | 1.34 |
|  |  |  | c92678_g1_i1 | 202.2 | 91.64 |
|  |  |  | c92678_g1_i2 | 58.22 | 4.88 |
|  |  |  | c100705_g1_i1 | 4.53 | 1.36 |
|  |  |  | c100705_g1_i2 | 6.06 | 2.92 |
|  |  |  | c100705_g1_i3 | 51.83 | 29.2 |
|  |  |  | c102967_g7_i4 | 0.15 | 0.58 |
|  |  |  | c131751_g1_i1 | 0.71 | 0.55 |
|  |  |  | c16655_g1_i1 | 0.91 | 0.67 |
| K04078 | groES, HSPE1 | chaperonin GroES | c69833_g1_i1 | 10.08 | 5.57 |
|  |  |  | c79318_g1_i1 | 0.31 | 0.64 |
|  |  |  | c79318_g2_i1 | 2.92 | 2.03 |
|  |  |  | c80514_g1_i1 | 365.37 | 131.48 |
|  |  |  | c80514_g1_i2 | 81.3 | 40.02 |
|  |  |  | c81878_g1_i1 | 221.27 | 98.26 |
|  |  |  | c92688_g1_i1 | 11.85 | 4.88 |
|  |  |  | c96127_g1_i1 | 228.63 | 69.05 |
|  |  |  | c96127_g2_i1 | 95.2 | 52.58 |
|  |  |  | c15076_g1_i1 | 33.87 | 16.49 |
| K11294 | NCL, NSR1 | nucleolin | c82960_g1_i1 | 807.15 | 326.85 |
|  |  |  | c87045_g1_i1 | 757.18 | 244.17 |
|  |  |  | c91379_g1_i1 | 191.8 | 77.75 |
|  |  |  | c100586_g2_i2 | 13.23 | 13.33 |
|  |  |  | c100586_g2_i3 | 2.88 | 2.31 |
|  |  |  | c114349_g1_i1 | 290.65 | 129.77 |
|  |  |  | c13864_g1_i1 | 52.44 | 98.53 |
|  |  |  | c151891_g1_i1 | 0.34 | 0.28 |
|  |  |  | c156550_g1_i1 | 0.36 | 0.56 |
|  |  |  | c156556_g1_i1 | 0.95 | 1.39 |
|  |  |  | c61729_g1_i1 | 6.79 | 8.44 |
| K05298 | GAPA | glyceraldehyde-3-phosphate dehydrogenase (NADP+) (phosphorylating) | c74489_g1_i1 | 0.44 | 0.67 |
|  |  |  | c92553_g1_i1 | 1398.03 | 773.19 |
|  |  |  | c100124_g1_i1 | 245.34 | 120.56 |
|  |  |  | c129596_g1_i1 | 9.32 | 6.28 |
|  |  |  | c173230_g1_i1 | 6.34 | 4.32 |
|  |  |  | c49037_g1_i1 | 1.06 | 1.21 |
|  |  |  | c49037_g2_i1 | 0.54 | 1.14 |
| K05592 | deaD, cshA | ATP-dependent RNA helicase DeaD | c115795_g1_i1 | 0.47 | 0.62 |
| K17279 | REEP5_6 | receptor expression-enhancing protein 5/6 | c81291_g1_i1 | 21.58 | 17.17 |
|  |  |  | c88982_g1_i1 | 12.19 | 4.37 |
|  |  |  | c88982_g2_i1 | 8.63 | 9.38 |
|  |  |  | c94749_g2_i1 | 0.46 | 1.44 |
|  |  |  | c98475_g1_i1 | 1.48 | 1.73 |
|  |  |  | c98475_g1_i2 | 0.78 | 1.73 |
|  |  |  | c151772_g1_i1 | 14.79 | 15.94 |
|  |  |  | c74310_g1_i1 | 5.52 | 5.65 |
|  |  |  | c81796_g1_i1 | 46.25 | 61.04 |
| K01177 | E3.2.1.2 | beta-amylase | c80088_g1_i1 | 2.49 | 2.96 |
|  |  |  | c80088_g1_i2 | 16.26 | 5.7 |
|  |  |  | c100002_g1_i1 | 518.14 | 177.32 |
|  |  |  | c101409_g1_i1 | 2.43 | 2.24 |
|  |  |  | c101409_g4_i1 | 15.37 | 8.27 |
|  |  |  | c101409_g5_i1 | 4.9 | 3.48 |
|  |  |  | c102292_g1_i1 | 0.82 | 0.63 |
|  |  |  | c102292_g1_i2 | 3.32 | 3.68 |
| K01602 | rbcS | ribulose-bisphosphate carboxylase small chain | c79996_g1_i1 | 13.47 | 16.7 |
|  |  |  | c118804_g1_i1 | 11.6 | 11.89 |
|  |  |  | c137036_g1_i1 | 11.12 | 7.75 |
|  |  |  | c154640_g1_i1 | 0.29 | 0.9 |
|  |  |  | c49308_g1_i1 | 9.14 | 3.5 |
|  |  |  | c62606_g2_i1 | 17777.74 | 6063.6 |
| K03841 | FBP, fbp | fructose-1,6-bisphosphatase I | c89324_g1_i1 | 111.8 | 41.28 |
|  |  |  | c95269_g1_i1 | 33.01 | 17.7 |
|  |  |  | c129944_g1_i1 | 1.04 | 0.4 |
|  |  |  | c131720_g1_i1 | 0.76 | 0.29 |
|  |  |  | c137696_g1_i1 | 187.03 | 156.4 |
| K01835 | pgm | phosphoglucomutase | c79313_g1_i1 | 1.69 | 1.85 |
|  |  |  | c85653_g1_i2 | 4.47 | 8.84 |
|  |  |  | c85653_g2_i1 | 1.14 | 7.59 |
|  |  |  | c85653_g3_i1 | 4.64 | 11.03 |
|  |  |  | c89888_g1_i1 | 28.23 | 19.86 |
|  |  |  | c97122_g1_i1 | 31.3 | 17.51 |
|  |  |  | c103450_g3_i1 | 9.73 | 1.49 |
| K00855 | PRK, prkB | phosphoribulokinase | c92987_g1_i1 | 3.45 | 0.96 |
|  |  |  | c92987_g2_i1 | 1.51 | 0.56 |
|  |  |  | c92987_g3_i1 | 2.21 | 0.26 |
|  |  |  | c99580_g1_i1 | 350.43 | 254.78 |
|  |  |  | c99580_g1_i2 | 177.99 | 177.58 |
|  |  |  | c109024_g1_i1 | 166.44 | 182.08 |
|  |  |  | c52723_g1_i1 | 1.01 | 0.61 |
| K17095 | ANXA7_11 | annexin A7/11 | c90975_g1_i1 | 34.03 | 32.32 |
|  |  |  | c91774_g1_i1 | 10.54 | 0.88 |
|  |  |  | c91901_g1_i1 | 0.88 | 0.42 |
|  |  |  | c91901_g1_i3 | 0.38 | 0.46 |
|  |  |  | c92775_g1_i1 | 11.48 | 13.76 |
|  |  |  | c92775_g2_i1 | 11.51 | 12.71 |
|  |  |  | c101479_g2_i1 | 5.58 | 13.03 |
|  |  |  | c162686_g1_i1 | 46.76 | 45.12 |
| K01759 | GLO1, gloA | lactoylglutathione lyase | c83935_g1_i1 | 0.64 | 0.43 |
|  |  |  | c86017_g1_i1 | 111.77 | 98.25 |
|  |  |  | c89630_g2_i1 | 41.03 | 31.31 |
|  |  |  | c89630_g3_i1 | 42.56 | 26.62 |
|  |  |  | c94548_g1_i1 | 111.04 | 71.21 |
|  |  |  | c96655_g1_i1 | 476.64 | 311.48 |
| K04371 | MAPK1_3 | mitogen-activated protein kinase 1/3 | c87746_g1_i1 | 24.36 | 25.95 |
| K14803 | PTC2_3 | protein phosphatase PTC2/3 | c91640_g1_i1 | 41.81 | 25.51 |
|  |  |  | c95982_g1_i1 | 15.02 | 11.98 |
|  |  |  | c95982_g1_i2 | 20.49 | 18.93 |
|  |  |  | c95982_g1_i3 | 1.37 | 4.66 |
|  |  |  | c98031_g1_i1 | 48.77 | 27.15 |
|  |  |  | c99564_g1_i1 | 8.62 | 3.45 |
|  |  |  | c99564_g2_i1 | 0.85 | 0.86 |
|  |  |  | c99564_g2_i2 | 5.47 | 5.28 |
|  |  |  | c99564_g2_i3 | 2.96 | 1.73 |
|  |  |  | c127404_g1_i1 | 7.82 | 5.74 |
| K09250 | CNBP | cellular nucleic acid-binding protein | c92409_g1_i1 | 21.14 | 21.38 |
|  |  |  | c92409_g1_i2 | 4.26 | 5.95 |
|  |  |  | c92409_g2_i1 | 33.41 | 20.1 |
|  |  |  | c92409_g2_i2 | 7.78 | 0.73 |
|  |  |  | c93811_g1_i1 | 18.59 | 11.85 |
|  |  |  | c93811_g1_i3 | 1.68 | 0.85 |
|  |  |  | c127206_g1_i1 | 14.26 | 17.7 |
|  |  |  | c57820_g1_i1 | 147.53 | 92.24 |
| K16911 | DDX21 | ATP-dependent RNA helicase DDX21 | c69620_g1_i1 | 3.24 | 5.04 |
|  |  |  | c102005_g1_i1 | 93.87 | 71.99 |
|  |  |  | c65073_g1_i1 | 3.81 | 11.81 |
| K00392 | sir | sulfite reductase (ferredoxin) | c98529_g1_i1 | 15.88 | 25.42 |
|  |  |  | c98529_g2_i1 | 18.21 | 10.61 |
|  |  |  | c98529_g3_i1 | 18.91 | 12.42 |
|  |  |  | c101061_g1_i1 | 22.31 | 17.61 |
|  |  |  | c101061_g1_i2 | 25.22 | 18.31 |
|  |  |  | c122810_g1_i1 | 5.98 | 8.33 |
|  |  |  | c141733_g1_i1 | 0.52 | 2.07 |
|  |  |  | c168981_g1_i1 | 0.69 | 0.74 |
| K03234 | EEF2 | elongation factor 2 | c85219_g1_i2 | 330.38 | 316.91 |
|  |  |  | c85219_g2_i1 | 214.43 | 99.25 |
| K02893 | RP-L23Ae, RPL23A | large subunit ribosomal protein L23Ae | c95384_g1_i1 | 165.85 | 117.61 |
|  |  |  | c164557_g1_i1 | 105.46 | 78.98 |
| K10257 | FAD8, desB | acyl-lipid omega-3 desaturase | c95001_g1_i1 | 0.56 | 0.32 |
|  |  |  | c101287_g1_i1 | 8.24 | 2.66 |
|  |  |  | c101287_g2_i1 | 7.17 | 2.64 |
|  |  |  | c101520_g1_i1 | 12.47 | 5.66 |
| K01783 | rpe, RPE | ribulose-phosphate 3-epimerase | c86572_g1_i1 | 19.96 | 16.87 |
|  |  |  | c86572_g1_i2 | 1.13 | 1.69 |
|  |  |  | c87018_g1_i1 | 308.22 | 224.5 |
| K03386 | E1.11.1.15, PRDX, ahpC | peroxiredoxin (alkyl hydroperoxide reductase subunit C) | c87416_g1_i1 | 273.92 | 111.1 |
|  |  |  | c108829_g1_i1 | 5.17 | 5.42 |
|  |  |  | c137992_g1_i1 | 162.89 | 83.74 |
| K08486 | STX1B_2_3 | syntaxin 1B/2/3 | c85312_g2_i1 | 3.4 | 0.2 |
|  |  |  | c100539_g1_i1 | 51.13 | 39.89 |
|  |  |  | c106805_g1_i1 | 24.79 | 32.62 |
| K18980 | EO | cinnamoyl-CoA reductase | c77805_g1_i1 | 32.36 | 71.16 |
|  |  |  | c895_g2_i1 | 0.65 | 0.09 |
|  |  |  | c53555_g1_i1 | 492.98 | 282.11 |
| K12855 | PRPF6, PRP6 | pre-mRNA-processing factor 6 | c76244_g1_i1 | 6 | 4.96 |
|  |  |  | c92988_g1_i1 | 3.56 | 3.06 |
|  |  |  | c92988_g1_i1 | 3.56 | 3.06 |
|  |  |  | c102874_g2_i2 | 23.8 | 19.76 |
|  |  |  | c102874_g2_i3 | 3.9 | 4.38 |
| K02988 | RP-S5, MRPS5, rpsE | small subunit ribosomal protein S5 | c80317_g1_i1 | 3.56 | 8.63 |
|  |  |  | c125577_g1_i1 | 247.8 | 128.22 |
| K17679 | MSS116 | ATP-dependent RNA helicase MSS116, mitochondrial | c91503_g1_i1 | 5.15 | 1.39 |
|  |  |  | c91503_g2_i1 | 1.41 | 2.61 |
|  |  |  | c98437_g2_i1 | 37.79 | 27.25 |
| K06268 | PPP3R, CNB | serine/threonine-protein phosphatase 2B regulatory subunit | c91877_g1_i1 | 36.64 | 24.17 |
|  |  |  | c92294_g1_i1 | 5.36 | 4.21 |
|  |  |  | c101619_g2_i1 | 2.18 | 6.04 |
|  |  |  | c106524_g1_i1 | 31.22 | 29.94 |
|  |  |  | c162911_g1_i1 | 20.83 | 15.68 |
| K12885 | RBMX, HNRNPG | heterogeneous nuclear ribonucleoprotein G | c83071_g2_i1 | 9.37 | 5.3 |
|  |  |  | c83071_g2_i2 | 58.01 | 33.13 |
|  |  |  | c99984_g1_i1 | 7.36 | 8.17 |
|  |  |  | c99984_g1_i2 | 29.91 | 23.61 |
| K02888 | RP-L21, MRPL21, rplU | large subunit ribosomal protein L21 | c76052_g1_i1 | 5.74 | 7.1 |
|  |  |  | c38126_g1_i1 | 115.7 | 68.61 |
| K02898 | RP-L26e, RPL26 | large subunit ribosomal protein L26e | c87237_g1_i1 | 236.54 | 207.52 |
|  |  |  | c87237_g2_i1 | 189.94 | 152.63 |
|  |  |  | c101088_g1_i1 | 1.96 | 2.9 |
|  |  |  | c101088_g1_i2 | 46.57 | 22.62 |
| K02941 | RP-LP0, RPLP0 | large subunit ribosomal protein LP0 | c77386_g1_i1 | 0.54 | 0.61 |
|  |  |  | c92543_g1_i1 | 542.43 | 262.82 |
| K03098 | APOD | apolipoprotein D and lipocalin family protein | c78256_g2_i1 | 183.73 | 182.23 |
|  |  |  | c117946_g1_i1 | 0.15 | 0.76 |
| K15633 | gpmI | 2,3-bisphosphoglycerate-independent phosphoglycerate mutase | c96327_g1_i1 | 161.06 | 85.36 |
| K03146 | THI4, THI1 | thiamine thiazole synthase | c81250_g1_i1 | 2532.34 | 1256 |
|  |  |  | c94727_g1_i1 | 0.99 | 2.09 |
|  |  |  | c94727_g2_i1 | 4.52 | 2.41 |
|  |  |  | c53329_g1_i1 | 0.59 | 0.44 |
| K02150 | ATPeV1E, ATP6E | V-type H+-transporting ATPase subunit E | c95452_g1_i1 | 18.93 | 26.05 |
|  |  |  | c102929_g5_i1 | 14.44 | 9.28 |
|  |  |  | c102929_g7_i1 | 3.09 | 4.46 |
| K09753 | CCR | cinnamoyl-CoA reductase | c87275_g1_i1 | 13.53 | 12.68 |
| K17991 | PXG | peroxygenase | c79859_g1_i1 | 0.87 | 5.94 |
|  |  |  | c92771_g1_i1 | 13.08 | 13.67 |
| K02870 | RP-L12e, RPL12 | large subunit ribosomal protein L12e | c87220_g1_i1 | 538.85 | 197.03 |
|  |  |  | c151665_g1_i1 | 0.41 | 1.25 |
| K08762 | DBI, ACBP | diazepam-binding inhibitor (GABA receptor modulator, acyl-CoA-binding protein) | c881_g1_i1 | 72.68 | 119.75 |
| K04124 | E1.14.11.15 | gibberellin 3-beta-dioxygenase | c137564_g1_i1 | 44.09 | 2.38 |
| K08244 | E2.7.9.4 | alpha-glucan, water dikinase | c93137_g1_i1 | 2.09 | 4.42 |
|  |  |  | c98691_g1_i1 | 5.68 | 10.75 |
| K12121 | PHYB | phytochrome B | c99802_g1_i1 | 7.62 | 15.22 |
| K10047 | VTC4 | inositol-phosphate phosphatase / L-galactose 1-phosphate phosphatase | c86135_g2_i1 | 9.32 | 5.4 |
|  |  |  | c87563_g1_i1 | 22.02 | 25.6 |
| K12124 | GI | GIGANTEA | c162928_g1_i1 | 25.48 | 43.27 |
| K02516 | PRMT5, HSL7 | type II protein arginine methyltransferase | c100100_g1_i2 | 4.49 | 11.26 |
| K04688 | RPS6KB | ribosomal protein S6 kinase beta | c85591_g1_i1 | 44.68 | 64.19 |
| K12667 | SWP1, RPN2 | oligosaccharyltransferase complex subunit delta (ribophorin II) | c97543_g1_i1 | 82.12 | 57.81 |
| K01583 | E4.1.1.19 | arginine decarboxylase | c61427_g2_i1 | 26.94 | 30.26 |
| K11462 | EED | polycomb protein EED | c60184_g1_i1 | 13.01 | 12.54 |
| K15135 | MED18 | mediator of RNA polymerase II transcription subunit 18 | c82680_g1_i1 | 15.21 | 11.67 |
| K02727 | PSMA3 | 20S proteasome subunit alpha 7 | c139070_g1_i1 | 29.14 | 28.46 |
