# Supplementary material for: Transcriptome Analysis of Cinnamomum chago: A Revelation of Candidate Genes for Abiotic Stress Response and Terpenoid and Fatty Acid Biosyntheses
Source: Front Genet. 2018 Nov 5;9:505. doi: 10.3389/fgene.2018.00505 (PMC6231050; doi:10.3389/fgene.2018.00505)
Supplement: Supplementary file 16 [file Table_11.DOC]

***Supplementary Material***

**Characterization of the de novo *Cinnamomum chago* (Lauraceae) transcriptome reveals candidate genes for terpenoid, fatty acid biosyntheses and abiotic stress**

**Authors:** Xue Zhang, Shi-Kang Shen *,

***Address for Correspondence:** Shi-Kang Shen, School of Life Sciences, Yunnan University, No. 2 Green lake North road Kunming, Yunnan, 650091, the People’s Republic of China. Telephone:+86-871-65031412; Fax:+86-871-65031412;

**E-mail:** yunda123456@126.com

**Table S11 Candidate genes related to heat stress in *C. chago*** transcriptome

| **KO ID** | **Gene** | **KEGG Annotation** | **Numbers of unineges** |
| --- | --- | --- | --- |
| K03283 | HSPA1_8 | heat shock 70kDa protein 1/8 | 32 |
| K13993 | HSP20 | HSP20 family protein | 29 |
| K13448 | CML | calcium-binding protein CML | 33 |
| K09286 | EREBP | EREBP-like factor | 32 |
| K03695 | clpB | ATP-dependent Clp protease ATP-binding subunit ClpB | 9 |
| K09419 | HSFF | heat shock transcription factor, other eukaryote | 20 |
| K04077 | groEL, HSPD1 | chaperonin GroEL | 16 |
| K04043 | dnaK | molecular chaperone DnaK | 7 |
| K04079 | htpG, HSP90A | molecular chaperone HtpG | 3 |
| K00134 | GAPDH, gapA | glyceraldehyde 3-phosphate dehydrogenase | 8 |
| K09503 | DNAJA2 | DnaJ homolog subfamily A member 2 | 13 |
| K09487 | HSP90B, TRA1 | heat shock protein 90kDa beta | 12 |
| K09489 | HSPA4 | heat shock 70kDa protein 4 | 6 |
| K14190 | VTC2_5 | GDP-L-galactose phosphorylase | 8 |
| K01919 | gshA | glutamate--cysteine ligase | 3 |
| K01858 | INO1, ISYNA1 | myo-inositol-1-phosphate synthase | 4 |
| K03089 | SIG3.3.1, rpoH | RNA polymerase sigma-32 factor | 1 |
| K17095 | ANXA7_11 | annexin A7/11 | 8 |
| K03687 | GRPE | molecular chaperone GrpE | 8 |
| K04460 | PPP5C | myo-inositol-1-phosphate synthase | 10 |
| K00286 | proC | pyrroline-5-carboxylate reductase | 1 |
| K09571 | FKBP4_5 | FK506-binding protein 4/5 | 7 |
| K02912 | RP-L32e, RPL32 | large subunit ribosomal protein L32e | 4 |
| K03686 | dnaJ | molecular chaperone DnaJ | 11 |
| K00966 | GMPP | mannose-1-phosphate guanylyltransferase | 4 |
| K14508 | NPR1 | regulatory protein NPR1 | 5 |
| K03098 | APOD | apolipoprotein D and lipocalin family protein | 2 |
| K10844 | ERCC2, XPD | DNA excision repair protein ERCC-2 | 3 |
| K09562 | HSPBP1 | hsp70-interacting protein | 3 |
| K03627 | MBF1 | putative transcription factor | 2 |
| K03875 | SKP2, FBXL1 | F-box and leucine-rich repeat protein 1 (S-phase kinase-associated protein 2) | 5 |
| K12160 | SUMO, SMT3 | small ubiquitin-related modifier | 3 |
| K03062 | PSMC1, RPT2 | 26S proteasome regulatory subunit T2 | 2 |
| K03029 | PSMD4, RPN10 | 26S proteasome regulatory subunit N10 | 1 |
| K04688 | RPS6KB | ribosomal protein S6 kinase beta | 1 |
| K10365 | CAPZB | capping protein (actin filament) muscle Z-line, beta | 1 |
| All |  | 46 | 317 |
