# Supplementary material for: Transcriptome Analysis of Cinnamomum chago: A Revelation of Candidate Genes for Abiotic Stress Response and Terpenoid and Fatty Acid Biosyntheses
Source: Front Genet. 2018 Nov 5;9:505. doi: 10.3389/fgene.2018.00505 (PMC6231050; doi:10.3389/fgene.2018.00505)
Supplement: Supplementary file 17 [file Table_12.DOC]

***Supplementary Material***

**Characterization of the de novo *Cinnamomum chago* (Lauraceae) transcriptome reveals candidate genes for terpenoid, fatty acid biosyntheses and abiotic stress**

**Authors:** Xue Zhang, Shi-Kang Shen *,

***Address for Correspondence:** Shi-Kang Shen, School of Life Sciences, Yunnan University, No. 2 Green lake North road Kunming, Yunnan, 650091, the People’s Republic of China. Telephone:+86-871-65031412; Fax:+86-871-65031412;

**E-mail:** yunda123456@126.com

**Table S12 The FPKM values of candidate genes related to heat stress in *C. chago*** transcriptome

| KO ID | Gene ID | KEGG Annotation | Unigene | Ccg1 | Ccg2 | Ccg3 |
| --- | --- | --- | --- | --- | --- | --- |
| K03283 | HSPA1_8 | heat shock 70kDa protein 1/8 | c73857_g1_i1 | 2.58 | 2.2 | 2.58 |
|  |  |  | c76923_g1_i1 | 4.66 | 7.2 | 9.08 |
|  |  |  | c76923_g2_i1 | 3.85 | 5.95 | 5.79 |
|  |  |  | c78399_g1_i1 | 13.06 | 22.1 | 16.93 |
|  |  |  | c83768_g1_i1 | 110.14 | 27.59 | 92.01 |
|  |  |  | c86493_g1_i1 | 118.35 | 24.4 | 63.4 |
|  |  |  | c89061_g1_i1 | 0.44 | 7.95 | 1.56 |
|  |  |  | c89061_g2_i1 | 0.65 | 5.59 | 3.31 |
|  |  |  | c90232_g1_i1 | 15.36 | 4.08 | 11.88 |
|  |  |  | c90512_g1_i3 | 4.52 | 4.96 | 8.21 |
|  |  |  | c91709_g1_i1 | 0.38 | 1.81 | 1.58 |
|  |  |  | c91709_g1_i2 | 3.3 | 2.38 | 2.21 |
|  |  |  | c91709_g1_i2 | 3.3 | 2.38 | 2.21 |
|  |  |  | c91709_g3_i1 | 10.86 | 4.51 | 4.14 |
|  |  |  | c92905_g1_i1 | 3.22 | 3.56 | 3.13 |
|  |  |  | c94933_g1_i1 | 5.97 | 12.07 | 8.72 |
|  |  |  | c95039_g1_i1 | 5.55 | 2.74 | 4.44 |
|  |  |  | c95569_g1_i1 | 55.44 | 46.47 | 67.29 |
|  |  |  | c96579_g1_i3 | 4.09 | 11.95 | 11.39 |
|  |  |  | c96579_g2_i1 | 1.59 | 15.17 | 17.49 |
|  |  |  | c100436_g1_i1 | 2.01 | 6.94 | 3.32 |
|  |  |  | c101374_g2_i1 | 148.45 | 120.74 | 174.97 |
|  |  |  | c102006_g1_i1 | 27.68 | 42.75 | 51.51 |
|  |  |  | c102006_g2_i1 | 437.49 | 214.07 | 399.43 |
|  |  |  | c102006_g2_i2 | 170.72 | 96.57 | 164.84 |
|  |  |  | c102006_g3_i1 | 37.43 | 36.82 | 73.19 |
|  |  |  | c102006_g3_i2 | 215.85 | 132.22 | 215.12 |
|  |  |  | c102006_g3_i3 | 26.35 | 37.81 | 25.13 |
|  |  |  | c127697_g1_i1 | 1.39 | 0.22 | 1.53 |
|  |  |  | c139597_g1_i1 | 97.29 | 17.38 | 77.26 |
|  |  |  | c166823_g1_i1 | 96.19 | 48.58 | 48.46 |
|  |  |  | c18497_g1_i1 | 61.83 | 90.02 | 173.81 |
| K13993 | HSP20 | HSP20 family protein | c69767_g1_i1 | 13.15 | 1.09 | 0.82 |
|  |  |  | c82398_g1_i1 | 71.27 | 14.06 | 53.43 |
|  |  |  | c84578_g1_i1 | 1.05 | 0.37 | 0.29 |
|  |  |  | c85162_g1_i1 | 150.7 | 36.48 | 63.86 |
|  |  |  | c91935_g1_i1 | 3.15 | 4.83 | 7.16 |
|  |  |  | c92602_g1_i1 | 0.29 | 3.02 | 50.62 |
|  |  |  | c92645_g1_i1 | 1.03 | 1.27 | 0.38 |
|  |  |  | c92761_g1_i1 | 24.85 | 2.35 | 66.02 |
|  |  |  | c94094_g1_i1 | 0.2 | 1.81 | 1.71 |
|  |  |  | c94367_g1_i1 | 20.92 | 3.72 | 19.17 |
|  |  |  | c94491_g1_i1 | 6.3 | 5.91 | 6.84 |
|  |  |  | c94491_g2_i1 | 1.04 | 1.08 | 1.13 |
|  |  |  | c97037_g1_i2 | 419.56 | 49.18 | 58.23 |
|  |  |  | c99069_g1_i1 | 701.94 | 174.94 | 281.59 |
|  |  |  | c99213_g1_i1 | 2.45 | 3.6 | 5.33 |
|  |  |  | c99481_g1_i1 | 224.25 | 121.24 | 449.44 |
|  |  |  | c100153_g1_i1 | 60.85 | 31.57 | 69.19 |
|  |  |  | c100153_g1_i1 | 60.85 | 31.57 | 69.19 |
|  |  |  | c103968_g1_i1 | 365.22 | 107.95 | 218.94 |
|  |  |  | c104527_g1_i1 | 319.19 | 122.43 | 287.58 |
|  |  |  | c112813_g1_i1 | 3.87 | 0.92 | 1.4 |
|  |  |  | c116564_g1_i1 | 37.31 | 48.97 | 59.8 |
|  |  |  | c119264_g1_i1 | 1.38 | 2.82 | 1.51 |
|  |  |  | c138837_g1_i1 | 150.13 | 3.39 | 39.94 |
|  |  |  | c141789_g1_i1 | 1.77 | 3.25 | 2.3 |
|  |  |  | c1547_g1_i1 | 19.22 | 3.44 | 16.12 |
|  |  |  | c15859_g1_i1 | 17.36 | 2.4 | 21.99 |
|  |  |  | c162663_g1_i1 | 393.94 | 20.68 | 326.98 |
|  |  |  | c21788_g1_i1 | 12.66 | 2.62 | 0.99 |
| K13448 | CML | calcium-binding protein CML | c69769_g1_i1 | 1.48 | 1.72 | 0.81 |
|  |  |  | c72868_g1_i1 | 11.59 | 7.27 | 16.03 |
|  |  |  | c74070_g1_i2 | 1.61 | 2.13 | 0.55 |
|  |  |  | c75983_g1_i1 | 4.94 | 4.34 | 5.88 |
|  |  |  | c76630_g1_i1 | 12.46 | 13.6 | 12.78 |
|  |  |  | c78431_g1_i1 | 0.64 | 0.99 | 0.48 |
|  |  |  | c84735_g1_i1 | 6.2 | 20.89 | 3.37 |
|  |  |  | c85289_g2_i1 | 7.22 | 5.58 | 4.86 |
|  |  |  | c85289_g2_i2 | 17.16 | 12.33 | 12.04 |
|  |  |  | c85865_g1_i1 | 206.31 | 165.49 | 308.71 |
|  |  |  | c88908_g1_i1 | 28.58 | 35.81 | 20.81 |
|  |  |  | c94476_g1_i1 | 24.02 | 12.04 | 17.83 |
|  |  |  | c94476_g2_i1 | 21.85 | 14.35 | 21.08 |
|  |  |  | c95343_g1_i1 | 3.91 | 3.83 | 4.76 |
|  |  |  | c98478_g1_i1 | 0.46 | 0.3 | 3.09 |
|  |  |  | c98478_g1_i2 | 0.79 | 2.05 | 1.32 |
|  |  |  | c98478_g1_i3 | 5.11 | 10.28 | 6.79 |
|  |  |  | c98801_g1_i2 | 10.48 | 9.86 | 8.02 |
|  |  |  | c98801_g1_i3 | 3.99 | 2.43 | 2.78 |
|  |  |  | c101976_g2_i1 | 7.61 | 3.45 | 4.4 |
|  |  |  | c101976_g2_i2 | 0.67 | 0.29 | 2.64 |
|  |  |  | c114035_g1_i1 | 79.42 | 69.55 | 66.03 |
|  |  |  | c115494_g1_i1 | 6.91 | 7.19 | 8.37 |
|  |  |  | c121284_g1_i1 | 0.74 | 1.36 | 1.22 |
|  |  |  | c150902_g1_i1 | 29.04 | 43.23 | 34.9 |
|  |  |  | c164220_g1_i1 | 107.3 | 105.9 | 204.84 |
|  |  |  | c165177_g1_i1 | 100.72 | 42.28 | 89.14 |
|  |  |  | c1946_g1_i1 | 2.54 | 10.16 | 5.54 |
|  |  |  | c2467_g1_i1 | 2.56 | 0.25 | 1.75 |
|  |  |  | c24929_g1_i1 | 3.47 | 3.92 | 15.34 |
|  |  |  | c58075_g1_i1 | 3.08 | 0.83 | 5.87 |
|  |  |  | c61200_g1_i1 | 1.69 | 0.47 | 2.41 |
|  |  |  | c64361_g3_i1 | 57.02 | 54.3 | 63.48 |
| K09286 | EREBP | EREBP-like factor | c68179_g1_i1 | 0.96 | 0.54 | 24.51 |
|  |  |  | c69316_g2_i1 | 66.8 | 52.81 | 106.76 |
|  |  |  | c74214_g1_i1 | 24.94 | 16.02 | 30.34 |
|  |  |  | c74214_g2_i1 | 23.18 | 17.49 | 26.46 |
|  |  |  | c77451_g1_i1 | 43.51 | 44.61 | 51.62 |
|  |  |  | c78299_g2_i1 | 1.58 | 16.8 | 53.3 |
|  |  |  | c81390_g1_i1 | 1.32 | 0.74 | 3.33 |
|  |  |  | c84324_g1_i1 | 3.94 | 12.21 | 12.85 |
|  |  |  | c84367_g1_i1 | 5.56 | 7.41 | 26.24 |
|  |  |  | c84627_g1_i1 | 15.21 | 15.85 | 19.35 |
|  |  |  | c88114_g2_i1 | 6.86 | 11.67 | 9.95 |
|  |  |  | c92630_g1_i1 | 134.13 | 131.51 | 131.33 |
|  |  |  | c92784_g1_i1 | 5.75 | 4.23 | 5.76 |
|  |  |  | c92806_g1_i1 | 11.89 | 8.32 | 61.76 |
|  |  |  | c94758_g1_i1 | 24.05 | 13.41 | 30.88 |
|  |  |  | c94862_g2_i1 | 1.63 | 1.23 | 15.65 |
|  |  |  | c96312_g4_i1 | 61.67 | 62.64 | 167.14 |
|  |  |  | c98828_g1_i1 | 170.75 | 306.68 | 406.98 |
|  |  |  | c98939_g1_i2 | 83.25 | 149.62 | 112.27 |
|  |  |  | c100057_g1_i1 | 268.74 | 367.18 | 566.12 |
|  |  |  | c100057_g1_i2 | 22.31 | 27.11 | 113.21 |
|  |  |  | c100057_g2_i1 | 42.2 | 47.59 | 210.91 |
|  |  |  | c119107_g1_i1 | 3.65 | 0.37 | 0.75 |
|  |  |  | c137498_g1_i1 | 206.65 | 202.16 | 234.35 |
|  |  |  | c137676_g1_i1 | 27.83 | 16.63 | 14.85 |
|  |  |  | c142150_g1_i1 | 1.52 | 15.3 | 1.84 |
|  |  |  | c146383_g1_i1 | 0.53 | 1.02 | 1.38 |
|  |  |  | c150972_g1_i1 | 44.39 | 34.22 | 46.67 |
|  |  |  | c16438_g1_i1 | 34.26 | 30.94 | 45.28 |
|  |  |  | c59312_g1_i1 | 2.43 | 9.57 | 35.03 |
|  |  |  | c59312_g2_i1 | 1.64 | 3.04 | 20.4 |
|  |  |  | c80244_g1_i1 | 118.52 | 96.54 | 139.16 |
| K03695 | clpB | ATP-dependent Clp protease ATP-binding subunit ClpB | c89727_g1_i1 | 1.03 | 0.43 | 0.46 |
|  |  |  | c93865_g1_i1 | 101.05 | 26.48 | 74.46 |
|  |  |  | c101436_g1_i2 | 0.8 | 2.32 | 2.45 |
|  |  |  | c101436_g2_i1 | 10.9 | 9.84 | 14.76 |
|  |  |  | c101436_g3_i1 | 1.04 | 2.67 | 2.49 |
|  |  |  | c101721_g4_i1 | 0.33 | 0.86 | 1.15 |
|  |  |  | c103465_g1_i11 | 8.06 | 1.56 | 7.84 |
|  |  |  | c137713_g1_i1 | 2.69 | 3.92 | 3.94 |
|  |  |  | c163325_g1_i1 | 1.54 | 2.52 | 2.7 |
| K09419 | HSFF | heat shock transcription factor, other eukaryote | c100884_g1_i2 | 6.09 | 15.24 | 17.72 |
|  |  |  | c73555_g1_i1 | 195.88 | 93.12 | 183.51 |
|  |  |  | c81050_g1_i1 | 5.61 | 2.98 | 6.42 |
|  |  |  | c81050_g2_i1 | 5.75 | 6.24 | 2.97 |
|  |  |  | c87396_g1_i1 | 0.22 | 1 | 0.25 |
|  |  |  | c91931_g1_i1 | 14.32 | 17 | 22.73 |
|  |  |  | c91931_g2_i1 | 5.88 | 8.11 | 14.9 |
|  |  |  | c92941_g1_i1 | 4.21 | 4.62 | 7.36 |
|  |  |  | c93254_g1_i1 | 29.87 | 18.26 | 28.38 |
|  |  |  | c93254_g2_i1 | 32.99 | 12.06 | 25.45 |
|  |  |  | c93327_g1_i1 | 0.54 | 1.5 | 1.47 |
|  |  |  | c93327_g2_i1 | 2.3 | 3.6 | 2.09 |
|  |  |  | c95282_g1_i1 | 2.64 | 3.15 | 3.22 |
|  |  |  | c95846_g1_i1 | 43.34 | 115.73 | 71.02 |
|  |  |  | c97118_g1_i2 | 0.84 | 3.59 | 3.86 |
|  |  |  | c100884_g3_i1 | 9.09 | 13.46 | 11.37 |
|  |  |  | c102357_g1_i1 | 0.87 | 1.64 | 1.11 |
|  |  |  | c14030_g1_i1 | 6.96 | 12.22 | 6.17 |
|  |  |  | c61553_g1_i1 | 33.08 | 46.47 | 62.84 |
|  |  |  | c64052_g1_i1 | 5.72 | 6.53 | 7.87 |
| K04077 | groEL, HSPD1 | chaperonin GroEL | c93571_g1_i1 | 15.68 | 7.36 | 14.69 |
|  |  |  | c93571_g2_i1 | 13.07 | 7.53 | 11.95 |
|  |  |  | c94339_g1_i1 | 0.78 | 1.03 | 1.56 |
|  |  |  | c94339_g2_i1 | 2.29 | 1.46 | 1.58 |
|  |  |  | c94339_g3_i1 | 0.15 | 1.25 | 0.49 |
|  |  |  | c94339_g5_i2 | 1.08 | 0.89 | 0.39 |
|  |  |  | c94339_g5_i3 | 0.51 | 0.24 | 0.46 |
|  |  |  | c101013_g1_i1 | 132.45 | 54.43 | 55.03 |
|  |  |  | c101013_g1_i2 | 107.2 | 83.57 | 99.37 |
|  |  |  | c101013_g1_i3 | 74.33 | 53.93 | 65.3 |
|  |  |  | c101013_g1_i4 | 92.13 | 74.4 | 104.06 |
|  |  |  | c102770_g4_i1 | 0.55 | 0.96 | 1.08 |
|  |  |  | c127266_g1_i1 | 0.49 | 1.03 | 1 |
|  |  |  | c162882_g1_i1 | 6.24 | 5.25 | 7.38 |
|  |  |  | c38676_g1_i1 | 0.9 | 1.39 | 1.84 |
|  |  |  | c39081_g1_i1 | 4.4 | 1.54 | 4.81 |
| K04043 | dnaK | molecular chaperone DnaK | c7546_g1_i1 | 2.05 | 0.39 | 0.56 |
|  |  |  | c102663_g20_i1 | 9.76 | 1.51 | 9.62 |
|  |  |  | c122192_g1_i1 | 1.83 | 0.46 | 0.66 |
|  |  |  | c125865_g1_i1 | 3.19 | 6.67 | 6.48 |
|  |  |  | c126818_g1_i1 | 0.77 | 0.78 | 1.27 |
|  |  |  | c163593_g1_i1 | 0.84 | 1.24 | 1.86 |
|  |  |  | c59992_g1_i1 | 1.42 | 1.8 | 2.56 |
| K04079 | htpG, HSP90A | molecular chaperone HtpG | c91514_g1_i1 | 68.8 | 23.84 | 72.6 |
|  |  |  | c99154_g1_i1 | 98.26 | 24.61 | 15.05 |
|  |  |  | c102663_g16_i1 | 9.51 | 1.45 | 9.38 |
| K00134 | GAPDH, gapA | glyceraldehyde 3-phosphate dehydrogenase | c87176_g1_i1 | 316.05 | 201.83 | 269.1 |
|  |  |  | c87176_g3_i1 | 289.27 | 193.86 | 216.54 |
|  |  |  | c91529_g1_i1 | 7.04 | 0.18 | 0.84 |
|  |  |  | c92588_g1_i1 | 1612.38 | 1281.37 | 1435.03 |
|  |  |  | c94616_g1_i1 | 2.54 | 4.31 | 0.88 |
|  |  |  | c94616_g2_i1 | 2.23 | 0.86 | 0.53 |
|  |  |  | c101520_g3_i1 | 0.11 | 3.01 | 0.47 |
|  |  |  | c21127_g1_i1 | 0.98 | 1.2 | 1.93 |
| K09503 | DNAJA2 | DnaJ homolog subfamily A member 2 | c77686_g1_i1 | 74.74 | 50.14 | 108.3 |
|  |  |  | c82261_g1_i1 | 2.57 | 4.65 | 4.07 |
|  |  |  | c85305_g1_i1 | 199.15 | 124.37 | 193.84 |
|  |  |  | c87266_g1_i1 | 635.28 | 490.59 | 601.2 |
|  |  |  | c89865_g1_i1 | 1.56 | 2.51 | 5.16 |
|  |  |  | c91470_g2_i1 | 76.8 | 61.66 | 112.75 |
|  |  |  | c96550_g1_i1 | 55.89 | 42.59 | 54.51 |
|  |  |  | c96550_g1_i2 | 394.81 | 676.7 | 516.48 |
|  |  |  | c96550_g1_i3 | 58.91 | 42.3 | 69.52 |
|  |  |  | c109743_g1_i1 | 0.32 | 0.99 | 2.08 |
|  |  |  | c155318_g1_i1 | 1.71 | 0.69 | 2.12 |
|  |  |  | c163069_g1_i1 | 20.68 | 37.93 | 50.95 |
|  |  |  | c26051_g1_i1 | 0.72 | 0.29 | 0.6 |
| K09487 | HSP90B, TRA1 | heat shock protein 90kDa beta | c87175_g1_i1 | 1.74 | 1.12 | 1.01 |
|  |  |  | c87175_g2_i1 | 2.31 | 0.87 | 1.26 |
|  |  |  | c87589_g1_i1 | 20.95 | 46.03 | 63.05 |
|  |  |  | c88380_g1_i1 | 3.08 | 1.99 | 1 |
|  |  |  | c88380_g1_i2 | 1.74 | 0.55 | 0.32 |
|  |  |  | c100349_g1_i1 | 10.08 | 34.57 | 16.99 |
|  |  |  | c100349_g1_i2 | 23.68 | 44.03 | 36.33 |
|  |  |  | c100349_g1_i3 | 12.08 | 41.17 | 20.3 |
|  |  |  | c152123_g1_i1 | 10.01 | 16.45 | 15.53 |
|  |  |  | c153841_g1_i1 | 16.92 | 22.96 | 24.25 |
|  |  |  | c53211_g1_i1 | 23.19 | 17.5 | 21.25 |
|  |  |  | c53211_g2_i1 | 15.74 | 12.6 | 14.83 |
| K09489 | HSPA4 | heat shock 70kDa protein 4 | c73056_g1_i1 | 0.88 | 1.16 | 1.75 |
|  |  |  | c85773_g1_i2 | 10.28 | 5.08 | 4.09 |
|  |  |  | c92496_g1_i3 | 42.5 | 41.66 | 59.86 |
|  |  |  | c92496_g2_i1 | 11.61 | 12.64 | 20.79 |
|  |  |  | c94872_g2_i1 | 5.39 | 12.3 | 12.78 |
|  |  |  | c104117_g1_i1 | 1.09 | 2.83 | 0.96 |
| K14190 | VTC2_5 | GDP-L-galactose phosphorylase | c89843_g1_i2 | 14.77 | 17.87 | 16.83 |
|  |  |  | c89843_g1_i3 | 15.51 | 8.02 | 6.92 |
|  |  |  | c94395_g1_i1 | 4.89 | 5.17 | 4.49 |
|  |  |  | c95589_g1_i2 | 170.92 | 106.69 | 124.71 |
|  |  |  | c95589_g1_i3 | 203.7 | 20.23 | 356.09 |
|  |  |  | c95589_g1_i4 | 215.32 | 156.38 | 318.47 |
|  |  |  | c61739_g1_i1 | 386.58 | 324.39 | 507.04 |
|  |  |  | c62967_g1_i1 | 316.27 | 268.04 | 585.07 |
| K01919 | gshA | glutamate--cysteine ligase | c858_g1_i1 | 12.94 | 16.8 | 20.97 |
|  |  |  | c18411_g1_i1 | 1.34 | 0.08 | 1.2 |
|  |  |  | c34684_g1_i1 | 0.9 | 0.47 | 1.64 |
| K01858 | INO1, ISYNA1 | myo-inositol-1-phosphate synthase | c94165_g1_i1 | 51.01 | 28.06 | 25.31 |
|  |  |  | c94165_g1_i2 | 25.35 | 25 | 35.65 |
|  |  |  | c66316_g1_i1 | 11.84 | 33.33 | 49.51 |
|  |  |  | c66316_g2_i1 | 35.08 | 28.53 | 48.33 |
| K03089 | SIG3.3.1, rpoH | RNA polymerase sigma-32 factor | c50041_g1_i1 | 2.45 | 4.8 | 7.1 |
| K17095 | ANXA7_11 | annexin A7/11 | c90975_g1_i1 | 34.03 | 32.32 | 61.68 |
|  |  |  | c91774_g1_i1 | 10.54 | 0.88 | 53.85 |
|  |  |  | c91901_g1_i1 | 0.88 | 0.42 | 0.28 |
|  |  |  | c91901_g1_i3 | 0.38 | 0.46 | 0.28 |
|  |  |  | c92775_g1_i1 | 11.48 | 13.76 | 22.35 |
|  |  |  | c92775_g2_i1 | 11.51 | 12.71 | 23.11 |
|  |  |  | c101479_g2_i1 | 5.58 | 13.03 | 14.22 |
|  |  |  | c162686_g1_i1 | 46.76 | 45.12 | 49.66 |
| K03687 | GRPE | molecular chaperone GrpE | c81972_g2_i1 | 4.01 | 1.49 | 7.27 |
|  |  |  | c99220_g1_i1 | 16.5 | 11.15 | 19.89 |
|  |  |  | c99220_g1_i3 | 12.76 | 8.71 | 13.74 |
|  |  |  | c99220_g1_i4 | 2.24 | 4.59 | 4.44 |
|  |  |  | c102517_g5_i1 | 9.03 | 26.89 | 27.97 |
|  |  |  | c102517_g6_i1 | 45 | 37.24 | 68.12 |
|  |  |  | c60150_g1_i1 | 14.45 | 16.46 | 24.85 |
|  |  |  | c60150_g1_i2 | 8.14 | 3.45 | 6.19 |
| K04460 | PPP5C | myo-inositol-1-phosphate synthase | c74501_g1_i1 | 1.3 | 0.35 | 0.72 |
|  |  |  | c86557_g1_i1 | 0.21 | 1.16 | 0.71 |
|  |  |  | c90343_g1_i3 | 0.45 | 0.92 | 0.33 |
|  |  |  | c91700_g1_i1 | 1.32 | 2.25 | 1.04 |
|  |  |  | c91700_g1_i2 | 9.16 | 12.14 | 12.28 |
|  |  |  | c97615_g2_i1 | 5.39 | 5.2 | 3.57 |
|  |  |  | c102608_g4_i1 | 0.65 | 1.43 | 0.39 |
|  |  |  | c102906_g2_i5 | 0.16 | 0.24 | 0.25 |
|  |  |  | c103107_g1_i1 | 1.34 | 4.05 | 1.67 |
|  |  |  | c103107_g1_i3 | 0.51 | 2 | 3.27 |
| K00286 | proC | pyrroline-5-carboxylate reductase | c96948_g1_i1 | 18.99 | 13.17 | 13.11 |
| K09571 | FKBP4_5 | FK506-binding protein 4/5 | c99266_g1_i1 | 1.51 | 1.14 | 2.53 |
|  |  |  | c99266_g1_i3 | 0.53 | 1.65 | 1.55 |
|  |  |  | c100949_g3_i1 | 3.65 | 1.04 | 2.29 |
|  |  |  | c101018_g4_i1 | 15.2 | 20.61 | 32.61 |
|  |  |  | c102756_g13_i1 | 11.13 | 13.03 | 10.98 |
|  |  |  | c31776_g1_i1 | 10.45 | 8.46 | 5.81 |
|  |  |  | c62468_g1_i1 | 9.11 | 29.82 | 28.49 |
| K02912 | RP-L32e, RPL32 | large subunit ribosomal protein L32e | c82849_g1_i1 | 263.49 | 107.17 | 196.1 |
|  |  |  | c91279_g2_i1 | 19.55 | 17.31 | 13.79 |
|  |  |  | c91279_g3_i1 | 107.9 | 63.69 | 66.17 |
|  |  |  | c36367_g2_i1 | 485.01 | 194.07 | 286.19 |
| K03686 | dnaJ | molecular chaperone DnaJ | c90889_g1_i1 | 3.12 | 7.37 | 6.59 |
|  |  |  | c90889_g2_i1 | 2.05 | 1.49 | 3.35 |
|  |  |  | c98925_g1_i1 | 6.19 | 5.29 | 4.65 |
|  |  |  | c98925_g2_i1 | 27.84 | 41.45 | 38.89 |
|  |  |  | c99758_g1_i1 | 3.42 | 2.25 | 2.64 |
|  |  |  | c100483_g1_i1 | 0.61 | 2.16 | 0.73 |
|  |  |  | c100483_g2_i1 | 8.73 | 18.2 | 20.77 |
|  |  |  | c100483_g3_i1 | 4.05 | 4.41 | 4.26 |
|  |  |  | c101425_g2_i1 | 1.94 | 1.56 | 1.57 |
|  |  |  | c18077_g2_i1 | 0.45 | 0.31 | 2.64 |
|  |  |  | c58508_g1_i1 | 13.18 | 11.1 | 14.89 |
| K00966 | GMPP | mannose-1-phosphate guanylyltransferase | c81753_g1_i1 | 0.5 | 1.26 | 0.32 |
|  |  |  | c93640_g4_i1 | 79.28 | 64.93 | 79.75 |
|  |  |  | c94026_g1_i1 | 49.57 | 40.92 | 43.44 |
|  |  |  | c63918_g1_i1 | 0.43 | 1.72 | 0.14 |
| K14508 | NPR1 | regulatory protein NPR1 | c82236_g1_i1 | 2.16 | 0.23 | 1.51 |
|  |  |  | c82236_g1_i2 | 0.66 | 1.34 | 1.51 |
|  |  |  | c83924_g1_i1 | 24.87 | 11.2 | 19.63 |
|  |  |  | c83924_g2_i1 | 7.07 | 8.09 | 10.82 |
|  |  |  | c90138_g1_i1 | 4.37 | 4.42 | 4.67 |
| K03098 | APOD | apolipoprotein D and lipocalin family protein | c78256_g2_i1 | 183.73 | 182.23 | 216.15 |
|  |  |  | c117946_g1_i1 | 0.15 | 0.76 | 1.19 |
| K10844 | ERCC2, XPD | DNA excision repair protein ERCC-2 | c97769_g1_i1 | 3.2 | 2.83 | 0.57 |
|  |  |  | c97769_g2_i1 | 2.88 | 2.56 | 3.13 |
|  |  |  | c97769_g2_i2 | 0.64 | 1.31 | 0.98 |
| K09562 | HSPBP1 | hsp70-interacting protein | c96163_g1_i1 | 59.3 | 25.03 | 44.42 |
|  |  |  | c100788_g1_i1 | 15.32 | 6.8 | 16.53 |
|  |  |  | c100788_g1_i2 | 2.72 | 5.32 | 4.13 |
| K03627 | MBF1 | putative transcription factor | c89358_g1_i2 | 155.32 | 289.6 | 365 |
|  |  |  | c127959_g1_i1 | 95.56 | 14.66 | 44.58 |
| K03875 | SKP2, FBXL1 | F-box and leucine-rich repeat protein 1 (S-phase kinase-associated protein 2) | c100565_g1_i1 | 3.84 | 7.31 | 3.76 |
|  |  |  | c100565_g1_i2 | 0.99 | 0.84 | 0.39 |
|  |  |  | c100565_g1_i3 | 22.81 | 26.51 | 23.78 |
|  |  |  | c100565_g2_i1 | 27.54 | 21.2 | 21.55 |
|  |  |  | c100565_g2_i2 | 13.62 | 6.9 | 4.17 |
| K12160 | SUMO, SMT3 | small ubiquitin-related modifier | c93111_g1_i1 | 22 | 22.53 | 29.77 |
|  |  |  | c93111_g3_i1 | 99.31 | 136.72 | 112.44 |
|  |  |  | c1824_g1_i1 | 284.31 | 348.55 | 204.91 |
| K03062 | PSMC1, RPT2 | 26S proteasome regulatory subunit T2 | c85437_g1_i1 | 166.3 | 168.73 | 212.4 |
|  |  |  | c96945_g1_i1 | 164.69 | 238.77 | 265.75 |
| K03029 | PSMD4, RPN10 | 26S proteasome regulatory subunit N10 | c97238_g1_i1 | 30.27 | 36.63 | 41.6 |
| K04688 | RPS6KB | ribosomal protein S6 kinase beta | c85591_g1_i1 | 44.68 | 64.19 | 62.27 |
| K10365 | CAPZB | capping protein (actin filament) muscle Z-line, beta | c99486_g12_i1 | 4 | 3.23 | 6.63 |
