# Supplementary material for: Transcriptome Analysis of Cinnamomum chago: A Revelation of Candidate Genes for Abiotic Stress Response and Terpenoid and Fatty Acid Biosyntheses
Source: Front Genet. 2018 Nov 5;9:505. doi: 10.3389/fgene.2018.00505 (PMC6231050; doi:10.3389/fgene.2018.00505)
Supplement: Supplementary file 18 [file Table_13.DOC]

***Supplementary Material***

**Characterization of the de novo *Cinnamomum chago* (Lauraceae) transcriptome reveals candidate genes for terpenoid, fatty acid biosyntheses and abiotic stress**

**Authors:** Xue Zhang, Shi-Kang Shen *,

***Address for Correspondence:** Shi-Kang Shen, School of Life Sciences, Yunnan University, No. 2 Green lake North road Kunming, Yunnan, 650091, the People’s Republic of China. Telephone:+86-871-65031412; Fax:+86-871-65031412;

**E-mail:** yunda123456@126.com

**Table S13 Candidate genes related to other abiotic stress in *C. chago*** transcriptome

| **KO ID** | **Gene** | **KEGG Annotation** | **Numbers of unineges** |
| --- | --- | --- | --- |
| **Response to herbicide** | | | |
| K02703 | psbA | photosystem II P680 reaction center D1 protein | 2 |
| All |  | 1 | 2 |
| **Response to nitrosative stress** | | | |
| K05916 | hmp, YHB1 | nitric oxide dioxygenase | 1 |
| K13035 | NIT4 | beta-cyano-L-alanine hydratase/nitrilase | 1 |
| All |  | 2 | 2 |
| **Regulation of response to red or far red light** | | | |
| K06689 | UBE2D_E, UBC4, UBC5 | ubiquitin-conjugating enzyme E2 D | 8 |
| K10609 | CUL4 | cullin 4 | 4 |
| K16241 | HY5 | transcription factor HY5 | 3 |
| K09613 | COPS5, CSN5 | COP9 signalosome complex subunit 5 | 3 |
| All |  | 4 | 18 |
| **Response to pH** | | | |
| K14638 | SLC15A3_4, PHT | solute carrier family 15 (peptide/histidine transporter), member 3/4 | 34 |
| K12761 | SNF1 | carbon catabolite-derepressing protein kinase | 3 |
| All |  | 2 | 37 |
| **Response to anoxia** | | | |
| K01568 | E4.1.1.1, pdc | pyruvate decarboxylase | 3 |
| All |  | 1 | 3 |
