# Supplementary material for: Transcriptome Analysis of Cinnamomum chago: A Revelation of Candidate Genes for Abiotic Stress Response and Terpenoid and Fatty Acid Biosyntheses
Source: Front Genet. 2018 Nov 5;9:505. doi: 10.3389/fgene.2018.00505 (PMC6231050; doi:10.3389/fgene.2018.00505)
Supplement: Supplementary file 19 [file Table_14.DOC]

***Supplementary Material***

**Characterization of the de novo *Cinnamomum chago* (Lauraceae) transcriptome reveals candidate genes for terpenoid, fatty acid biosyntheses and abiotic stress**

**Authors:** Xue Zhang, Shi-Kang Shen *,

***Address for Correspondence:** Shi-Kang Shen, School of Life Sciences, Yunnan University, No. 2 Green lake North road Kunming, Yunnan, 650091, the People’s Republic of China. Telephone:+86-871-65031412; Fax:+86-871-65031412;

**E-mail:** yunda123456@126.com

**Table S14 The FPKM values of candidate genes related to other abiotic stress in *C. chago* transcriptome**

| KO ID | Gene ID | | KEGG Annotation | Unigene | Ccg1 | | Ccg2 | Ccg3 | Mean |
| --- | --- | --- | --- | --- | --- | --- | --- | --- | --- |
| **Response to herbicide** | | | | | | | | | |
| K02703 | | psbA | photosystem II P680 reaction center D1 protein 1 | c97751_g1_i1 | | 315.75 | 387.93 | 440.46 | 381.38 |
|  | |  |  | c101434_g1_i1 | | 3.9 | 4.7 | 6.17 | 4.92 |
| **Response to nitrosative stress** | | | |  | |  |  |  |  |
| K05916 | | hmp, YHB1 | nitric oxide dioxygenase | c41281_g1_i1 | | 1.57 | 0.21 | 1.44 | 1.07 |
| K13035 | | NIT4 | beta-cyano-L-alanine hydratase/nitrilase 2 | c103066_g1_i1 | | 109.94 | 74.49 | 86.39 | 90.27 |
| **Regulation of response to red or far red light** | | | |  | |  |  |  |  |
| K06689 | | UBE2D_E, UBC4, UBC5 | ubiquitin-conjugating enzyme E2 D | c73377_g1_i2 | | 169.6 | 159.14 | 145.26 | 158 |
|  | |  |  | c96146_g1_i1 | | 24.17 | 30.59 | 25.33 | 26.7 |
|  | |  |  | c96146_g1_i2 | | 217.14 | 167.87 | 196.96 | 193.99 |
|  | |  |  | c96146_g2_i1 | | 135.32 | 115.21 | 171.76 | 140.76 |
|  | |  |  | c97128_g2_i3 | | 6.91 | 6.69 | 1.76 | 5.12 |
|  | |  |  | c116455_g1_i1 | | 2.71 | 3.79 | 2.97 | 3.16 |
|  | |  |  | c137817_g1_i1 | | 276.98 | 181.97 | 178.17 | 212.37 |
|  | |  |  | c20646_g1_i1 | | 247.88 | 329.57 | 369.79 | 315.75 |
| K10609 | | CUL4 | cullin 4 | c69070_g1_i1 | | 4.26 | 11.89 | 8.15 | 8.1 |
|  | |  |  | c69070_g2_i1 | | 10.69 | 11.16 | 7.11 | 9.65 |
|  | |  |  | c98553_g1_i1 | | 7.11 | 10.2 | 11.28 | 9.53 |
|  | |  |  | c98553_g1_i2 | | 35.08 | 46 | 57.02 | 46.03 |
| K16241 | | HY5 | transcription factor HY5 | c78112_g1_i2 | | 3.34 | 0.81 | 0.93 | 1.69 |
|  | |  |  | c87870_g1_i2 | | 1.06 | 2.7 | 3.07 | 2.28 |
|  | |  |  | c1550_g1_i1 | | 15.71 | 9.27 | 18.82 | 14.6 |
| K09613 | | COPS5, CSN5 | COP9 signalosome complex subunit 5 | c101294_g1_i1 | | 57.36 | 52.35 | 75.28 | 61.66 |
|  | |  |  | c1447_g1_i1 | | 18.8 | 9.23 | 12.63 | 13.55 |
|  | |  |  | c64736_g1_i1 | | 11.3 | 6.05 | 10.33 | 9.23 |
| **Response to pH** | | |  |  | |  |  |  |  |
| K14638 | | SLC15A3_4, PHT | solute carrier family 15 (peptide/histidine transporter), member 3/4 | c69689_g1_i1 | | 1.43 | 1.59 | 0.17 | 1.06 |
|  | |  |  | c69689_g2_i1 | | 1.45 | 1.35 | 2.09 | 1.63 |
|  | |  |  | c70946_g1_i1 | | 9.16 | 9.99 | 10.82 | 9.99 |
|  | |  |  | c72182_g2_i1 | | 0.43 | 1.17 | 0.52 | 0.71 |
|  | |  |  | c76363_g2_i1 | | 1.33 | 0.77 | 0.61 | 0.9 |
|  | |  |  | c79635_g2_i1 | | 25.76 | 17.12 | 15.78 | 19.55 |
|  | |  |  | c86003_g1_i1 | | 5.51 | 5.95 | 2.88 | 4.78 |
|  | |  |  | c86830_g1_i1 | | 0 | 0.87 | 0.36 | 0.41 |
|  | |  |  | c89551_g1_i1 | | 0.92 | 0.34 | 2.84 | 1.37 |
|  | |  |  | c89985_g1_i1 | | 4.33 | 15.29 | 7.49 | 9.04 |
|  | |  |  | c95832_g1_i1 | | 8.01 | 22.15 | 16.55 | 15.57 |
|  | |  |  | c95844_g1_i2 | | 4.68 | 2.53 | 5.83 | 4.35 |
|  | |  |  | c96793_g1_i1 | | 2.07 | 3.92 | 3.38 | 3.12 |
|  | |  |  | c96793_g2_i1 | | 1.44 | 1.73 | 2.41 | 1.86 |
|  | |  |  | c96826_g1_i1 | | 23.18 | 25.83 | 30.39 | 26.47 |
|  | |  |  | c97297_g1_i1 | | 44.32 | 40.24 | 54.38 | 46.31 |
|  | |  |  | c97437_g1_i1 | | 27.47 | 42.42 | 40.63 | 36.84 |
|  | |  |  | c98092_g2_i1 | | 12.32 | 4.95 | 1.22 | 6.16 |
|  | |  |  | c99184_g3_i1 | | 1.76 | 0.61 | 0.63 | 1 |
|  | |  |  | c100545_g1_i1 | | 51.16 | 14.18 | 26.77 | 30.7 |
|  | |  |  | c100545_g2_i2 | | 7.52 | 4.9 | 5.26 | 5.9 |
|  | |  |  | c100954_g3_i2 | | 0.35 | 3.32 | 0.19 | 1.29 |
|  | |  |  | c101285_g2_i1 | | 60.17 | 60.24 | 61.26 | 60.56 |
|  | |  |  | c10163_g1_i1 | | 6.52 | 0.54 | 0.92 | 2.66 |
|  | |  |  | c101711_g2_i1 | | 0.64 | 1.18 | 0.35 | 0.72 |
|  | |  |  | c102873_g2_i1 | | 0.52 | 1.42 | 0.41 | 0.78 |
|  | |  |  | c103032_g1_i1 | | 2.08 | 3.19 | 1.48 | 2.25 |
|  | |  |  | c103032_g2_i1 | | 2.23 | 2.29 | 9.67 | 4.73 |
|  | |  |  | c103032_g3_i1 | | 3.61 | 2.44 | 13.94 | 6.66 |
|  | |  |  | c103032_g4_i1 | | 4.13 | 3.02 | 3.84 | 3.66 |
|  | |  |  | c104540_g1_i1 | | 1.54 | 0.98 | 0.95 | 1.16 |
|  | |  |  | c106538_g1_i1 | | 2.01 | 1.23 | 0.09 | 1.11 |
|  | |  |  | c142435_g1_i1 | | 5.28 | 3.46 | 1.38 | 3.37 |
|  | |  |  | c157211_g1_i1 | | 5.06 | 1.41 | 0.33 | 2.27 |
| K12761 | | SNF1 | carbon catabolite-derepressing protein kinase | c90605_g1_i1 | | 1.84 | 7.14 | 3.64 | 4.21 |
|  | |  |  | c95690_g1_i1 | | 77.05 | 38.46 | 92.3 | 69.27 |
|  | |  |  | c62518_g1_i1 | | 1.89 | 10.85 | 3.59 | 5.44 |
| **Response to anoxia** | | |  |  | |  |  |  |  |
| K01568 | | E4.1.1.1, pdc | pyruvate decarboxylase | c91792_g1_i1 | | 93.01 | 57.36 | 58.12 | 69.5 |
|  | |  |  | c91792_g2_i1 | | 81.86 | 44.33 | 37.28 | 54.49 |
|  | |  |  | c59262_g2_i1 | | 37.86 | 24.1 | 35.53 | 32.5 |
