# Supplementary material for: Transcriptome Analysis of Cinnamomum chago: A Revelation of Candidate Genes for Abiotic Stress Response and Terpenoid and Fatty Acid Biosyntheses
Source: Front Genet. 2018 Nov 5;9:505. doi: 10.3389/fgene.2018.00505 (PMC6231050; doi:10.3389/fgene.2018.00505)
Supplement: Supplementary file 20 [file Table_15.DOC]

***Supplementary Material***

**Characterization of the de novo *Cinnamomum chago* (Lauraceae) transcriptome reveals candidate genes for terpenoid, fatty acid biosyntheses and abiotic stress**

**Authors:** Xue Zhang, Shi-Kang Shen *,

***Address for Correspondence:** Shi-Kang Shen, School of Life Sciences, Yunnan University, No. 2 Green lake North road Kunming, Yunnan, 650091, the People’s Republic of China. Telephone:+86-871-65031412; Fax:+86-871-65031412;

**E-mail:** yunda123456@126.com

**Table S16 Characterization of polymorphic SSRs in the *C. chago* transcriptome by novel markers**

| **Locus** | **Repeat motif** | **Size** | **Primer sequence (5’-3’)** | **Tm**  **(℃)** | ***Na*** | ***Ho*** | ***He*** | ***PIC*** | **HWE** |
| --- | --- | --- | --- | --- | --- | --- | --- | --- | --- |
| **C1** | (AACC)5 | 270 | CATGTTCAAAGCCATTGTGG | 55 | 12 | 1.000 | 0.733 | 0.872 | 0.141ns |
|  |  |  | ATAAATCAAGCCCAATCCCC |  |  |  |  |  |  |
| **C3** | (GA)8 | 207 | CGGCTGCTATGGAAGAGAGT | 53 | 6 | 0.333 | 0.267 | 0.310 | 1.00ns |
|  |  |  | TTGGATTGACTGCAGTTTGC |  |  |  |  |  |  |
| **C11** | (AG)8 | 260 | GCAATTCCGATACTGCATCA | 55 | 10 | 0.933 | 0.692 | 0.826 | 0.654ns |
|  |  |  | AGCCGAAGAAACCCTACACA |  |  |  |  |  |  |
| **C13** | (AG)6 | 220 | GGGTTTGGGAGCAGATGTAA | 54 | 4 | 0.533 | 0.511 | 0.510 | 0.968ns |
|  |  |  | CCCCACAAATTCCTTCCTTT |  |  |  |  |  |  |
| **C14** | (TG)9 | 270 | TTTCTCCGATTTTACCAAACAGA | 54 | 6 | 1.000 | 0.658 | 0.656 | 0.227ns |
|  |  |  | CTGGGACTTGGATGAGGAGA |  |  |  |  |  |  |
| **C15** | (AGA)5 | 192 | TTGTCGATTCTTTCCAACCC | 53 | 4 | 1.000 | 0.544 | 0.470 | 0.0296* |
|  |  |  | TTGTTGCACGAAGAACCAAC |  |  |  |  |  |  |
| **C16** | (CAT)5 | 280 | GAAGCAACACCAGCAAAACA | 54 | 6 | 0.933 | 0.578 | 0.649 | 0.055ns |
|  |  |  | CAGCAATTCCAGCAAAAGACT |  |  |  |  |  |  |
| **C17** | (TTC)5 | 216 | GGAACCCACCAAAATCCTTT | 54 | 7 | 0.933 | 0.694 | 0.715 | 0.636ns |
|  |  |  | AGCATATCAGGCGACGTTTC |  |  |  |  |  |  |
| **C18** | (CA)6 | 226 | AACATCGAGAAGAGCTCGGA | 58 | 10 | 0.867 | 0.625 | 0.712 | 0.543ns |
|  |  |  | GTGCTCGATCCCTCACTCAT |  |  |  |  |  |  |
| **C19** | (CAG)5 | 200 | ACCCTAGCCCTCAAATGACC | 58 | 6 | 0.267 | 0.256 | 0.310 | 0.020* |
|  |  |  | TTCCCCTTGGAGATCCTCTT |  |  |  |  |  |  |
| **C20** | (CAT)5 | 227 | AATTGCCGACCCAGTAAACA | 54 | 3 | 0.333 | 0.236 | 0.240 | 0.943ns |
|  |  |  | TTCCAGCCATTGCTTTCTCT |  |  |  |  |  |  |
| **C21** | (CGG)5 | 263 | CTCTTCCTCCTCCTCGTCCT | 58 | 5 | 0.867 | 0.578 | 0.578 | 0.109ns |
|  |  |  | TGGGTAAGAAGAAGTCAGCTATCA |  |  |  |  |  |  |
| **C23** | (GA)9 | 204 | CATGTCCACAAAAACCATGC | 53 | 7 | 0.667 | 0.536 | 0.605 | 0.418ns |
|  |  |  | GCCCGGTATATTGGCAGTTA |  |  |  |  |  |  |
| **C27** | (TC)6 | 238 | CCAAAACAAAAACAGAAATCCC | 52 | 3 | 0.133 | 0.111 | 0.131 | 0.994ns |
|  |  |  | ATTTCATCATCTTCGCCACC |  |  |  |  |  |  |
| **C30** | (AG)8 | 214 | AGAAGACGGGAAACAGACCA | 54 | 4 | 0.500 | 0.442 | 0.521 | 0.015* |
|  |  |  | TTTGAAGCCTTCCCCATTTA |  |  |  |  |  |  |
| **C32** | (CATA)5 | 243 | GGGGGATATGAAGGCAATTT | 53 | 6 | 0.933 | 0.578 | 0.625 | 0.248ns |
|  |  |  | ACCCCCTTTTGCACTAACAA |  |  |  |  |  |  |
| **C34** | (GA)8 | 241 | CAGGAGAAAGAGTGGCGGTA | 56 | 3 | 0.733 | 0.400 | 0.450 | 0.229ns |
|  |  |  | CCGAAATCGTCTGCAATTTT |  |  |  |  |  |  |
| **C36** | (AC)7 | 280 | GGGTTTTGCAAATGCTCAAT | 53 | 5 | 0.933 | 0.600 | 0.607 | 0.002** |
|  |  |  | TCAGCGTGCACAATCTGTAAG |  |  |  |  |  |  |
| **C37** | (GGA)5 | 222 | GAGGAAGTGTACGGGGTTGA | 58 | 7 | 0.900 | 0.631 | 0.722 | 0.004** |
|  |  |  | TCCATCTCTCCTTCTTCCGA |  |  |  |  |  |  |
| **C39** | (AG)8 | 191 | GGGAACGATTTTGGGAAGAT | 55 | 4 | 0.833 | 0.508 | 0.616 | 0.326ns |
|  |  |  | TTTCGATTCCCAACTCCAAG |  |  |  |  |  |  |
| **C41** | (ATC)6 | 241 | TTCTTCATTTTGACCAGGGG | 56 | 8 | 0.800 | 0.531 | 0.653 | 0.173ns |
|  |  |  | TCAGGTGCTATGCTGCATTC |  |  |  |  |  |  |
| **C42** | (AAT)5 | 252 | CGCCCCTTCACACAATCTAT | 56 | 7 | 0.400 | 0.367 | 0.459 | 0.004** |
|  |  |  | ATGAATCAATCGCCAGCTTC |  |  |  |  |  |  |
| **C43** | (AG)7 | 203 | AAAGGGAAGAAAAAGCGAGC | 55 | 7 | 0.933 | 0.672 | 0.716 | 0.288ns |
|  |  |  | AAAACAACACAAAAAGCCCG |  |  |  |  |  |  |
| **C44** | (AGC)5 | 226 | TCTCCTTCCAAACCATCGAC | 53 | 9 | 0.700 | 0.531 | 0.675 | 0.543ns |
|  |  |  | GGAGATATCAGGTGGGAGCA |  |  |  |  |  |  |
| **C45** | (GAG)5 | 188 | TAACGGAGGTGTTGGAGGAC | 58 | 6 | 1.000 | 0.669 | 0.762 | 0.211ns |
|  |  |  | AGCTGCAAAAGCAGTTCGTT |  |  |  |  |  |  |
| **C47** | (TCA)5 | 256 | CATCTGACATGGCGTCACTT | 58 | 6 | 0.767 | 0.531 | 0.569 | 0.865ns |
|  |  |  | GGCTTCTCAGATGGTCAAGC |  |  |  |  |  |  |
| **C49** | (TC)8 | 273 | AGCTTCTACCAAGCGCAAAG | 56 | 11 | 0.933 | 0.717 | 0.861 | 0.105ns |
|  |  |  | GAGAGAGTTACACATAAAATTCCCG |  |  |  |  |  |  |
| **C50** | (CA)6 | 234 | GGAACAAAGTGGCCCAAATA | 56 | 6 | 1.000 | 0.669 | 0.706 | 0.171ns |
|  |  |  | ATGGCATATGTGGTGCTTCA |  |  |  |  |  |  |
| **C51** | (GAT)5 | 193 | CAACCCCAACAAAGCTAGGA | 53 | 5 | 1.000 | 0.581 | 0.582 | 0.137ns |
|  |  |  | AACCCCAAATGAGTCAGCAG |  |  |  |  |  |  |
| **C53** | (TGA)5 | 251 | TGCTTTCATGGCGATTGTTA | 57 | 3 | 0.133 | 0.111 | 0.131 | 0.994ns |
|  |  |  | AGCAGCTCTGATCACCCACT |  |  |  |  |  |  |
| **C54** | (GAG)5 | 225 | GCGGTTGTGGAGAATGATTT | 57 | 6 | 1.000 | 0.647 | 0.650 | 0.000*** |
|  |  |  | CCCAGAGTCCACATCGTCTT |  |  |  |  |  |  |
| **C56** | (AT)6 | 230 | GCTGCTTGAGTCTCATTTGC | 55 | 4 | 0.700 | 0.508 | 0.540 | 0.104ns |
|  |  |  | TGCTGCAAATATTATTATTGGGTT |  |  |  |  |  |  |
| **C60** | (AT)6 | 215 | CGCCTTTGATGATTCACGTA | 56 | 6 | 1.000 | 0.614 | 0.615 | 0.636ns |
|  |  |  | CCTGAAACGTGTAGAACGCA |  |  |  |  |  |  |
| **C63** | (CTG)5 | 226 | CGTGGGACTAGGTTGCTGTT | 59 | 7 | 1.000 | 0.633 | 0.662 | 0.719ns |
|  |  |  | ACACAGAGAGATGGATGCCC |  |  |  |  |  |  |
| **C64** | (TG)8 | 265 | TCTAAGGCAGTTTCGGGATG | 56 | 5 | 1.000 | 0.592 | 0.550 | 0.000*** |
|  |  |  | TGGGAGAGAAATCCTTGGTG |  |  |  |  |  |  |
| **C65** | (TG)6 | 246 | ATGTGATTTTGGTTTTGGCA | 53 | 3 | 0.933 | 0.558 | 0.535 | 0.018* |
|  |  |  | CATTGACCACAGTGAGCCTG |  |  |  |  |  |  |
| **C67** | (TC)7 | 199 | TCCCCATACTCGCACTCTTC | 56 | 4 | 0.267 | 0.233 | 0.298 | 0.034* |
|  |  |  | CTTTCAAGGATCCAATCCGA |  |  |  |  |  |  |
| **C68** | (AG)7 | 257 | GGTGTGAATTGGAGGAAGGA | 57 | 6 | 1.000 | 0.622 | 0.645 | 0.535ns |
|  |  |  | ATGGCCACATACACAGACGA |  |  |  |  |  |  |
| **C69** | (TC)7 | 213 | CTGATATTGTGACCGGAGCA | 53 | 6 | 1.000 | 0.672 | 0.693 | 0.482ns |
|  |  |  | CACCGATCTTTTACCCCAAA |  |  |  |  |  |  |
| **C71** | (AGA)5 | 270 | GAAGAAATGCGAAACCCAAA | 55 | 7 | 0.800 | 0.511 | 0.605 | 0.994ns |
|  |  |  | ATTCCCAAAACACAAAACCG |  |  |  |  |  |  |
| **C72** | (TC)6 | 258 | ACGACACAGGAACAATGCTG | 57 | 6 | 0.600 | 0.403 | 0.448 | 1.000ns |
|  |  |  | ATCCTATGTTTCCCCACACG |  |  |  |  |  |  |
| **C73** | (GA)7 | 279 | CACTTGCAGTCGAAGCAAAA | 56 | 3 | 0.633 | 0.475 | 0.501 | 0.170ns |
|  |  |  | GTGGTGACGGTCTTTTCGAT |  |  |  |  |  |  |
| **C74** | (CTT)5 | 279 | CACTTGCAGTCGAAGCAAAA | 56 | 5 | 1.000 | 0.611 | 0.640 | 0.319ns |
|  |  |  | GTGGTGACGGTCTTTTCGAT |  |  |  |  |  |  |
| **C75** | (AG)6 | 240 | GTTGGGAAAGGAAGGGGTTA | 58 | 8 | 0.933 | 0.669 | 0.738 | 0.057ns |
|  |  |  | GCACGGAGAGAAAGCAGAGA |  |  |  |  |  |  |
| **C77** | (CAG)5 | 240 | CCATCCAAGCAACCAACTCT | 58 | 8 | 0.800 | 0.522 | 0.685 | 1.000ns |
|  |  |  | ACAACATTGCTAAGGCCACC |  |  |  |  |  |  |
| **C80** | (ATC)5 | 204 | TTGCAAATCATTCAAGGGACT | 56 | 6 | 0.900 | 0.564 | 0.555 | 0.710ns |
|  |  |  | CCCCAACATAGTTGAGACACG |  |  |  |  |  |  |
| **C82** | (CTT)5 | 228 | GAACTATTGGCGAAAGCTGC | 57 | 2 | 0.133 | 0.111 | 0.124 | 0.773ns |
|  |  |  | AAATCCAGAATCCCAGACCC |  |  |  |  |  |  |
| **C84** | (CCT)6 | 252 | ATTTCGCCTCAAGGTGTGAC | 57 | 3 | 0.000 | 0.222 | 0.343 | 0.000*** |
|  |  |  | TTAATCCGTCGGGAAGAGTG |  |  |  |  |  |  |
| **C85** | (TC)8 | 221 | TTCTGGGGGTAGCATGTAGG | 58 | 4 | 1.000 | 0.600 | 0.621 | 0.210ns |
|  |  |  | GGAAGTGGATTTCACCTGGA |  |  |  |  |  |  |
| **C87** | (GA)9 | 226 | TTGGCCATGTATGCTGTTGT | 56 | 13 | 1.000 | 0.761 | 0.883 | 0.034* |
|  |  |  | GAAATTGCAGCCAAACCATC |  |  |  |  |  |  |
| **C88** | (ATC)5 | 201 | TGCATATTGTCAAGACCCCA | 56 | 4 | 0.667 | 0.422 | 0.521 | 0.353ns |
|  |  |  | CATGCGCTGGTCTTAATCCT |  |  |  |  |  |  |
| **C90** | (TC)6 | 277 | TCCCCTTCTGTAACTAGGACACAT | 58 | 8 | 0.867 | 0.569 | 0.631 | 0.000*** |
|  |  |  | AGCTGAACGAGGAGCAAAAA |  |  |  |  |  |  |
| **C91** | (CGG)5 | 262 | GAAGGGAAGGAGCGAGTTCT | 57 | 6 | 0.500 | 0.331 | 0.402 | 1.000ns |
|  |  |  | AAACCTCCTCGCAAAATCCT |  |  |  |  |  |  |
| **C94** | (AG)8 | 245 | AGAATTCTGGGTTGCCAGTG | 58 | 10 | 0.800 | 0.636 | 0.779 | 0.160ns |
|  |  |  | TCCACCAATCCTCTCTCTGC |  |  |  |  |  |  |
| **C95** | (AG)8 | 268 | GACCGATGCATTGTGTCTTG | 58 | 3 | 0.933 | 0.514 | 0.421 | 0.015* |
|  |  |  | ACCCATTAGACCCACCCTTC |  |  |  |  |  |  |
| **Mean** |  |  |  |  | 5.982 | 0.759 | 0.519 | 0.570 |  |
| **Total** |  |  |  |  | 329 |  |  |  |  |

**Note:** **Tm,** Optimized annealing temperature; ***Na***, No. of Alleles; ***Ho***, observed heterozygosity; ***H_E_***, Expected Heterozygosity; ***PIC***, Polymorphism information content; ***HWE***, Hardy-Weinberg equilibrium.
